# NH_3_‐Guided Low‐Temperature Nanostructural Refinement Boosts Visible‐Light‐Driven H_2_O_2_ Synthesis in Ionic Carbon Nitrides

**DOI:** 10.1002/adma.202510585

**Published:** 2025-11-16

**Authors:** Jaya Bharti, Jokotadeola Odutola, Zahra Hajiahmadi, Karlo Nolkemper, Zhihong Tian, Haijian Tong, Vitaliy Shvalagin, Thomas D. Kühne, Tero‐Petri Ruoko, Christian Mark Pelicano

**Affiliations:** ^1^ Department of Colloid Chemistry Max Planck Institute of Colloids and Interfaces 14476 Potsdam Germany; ^2^ Chemistry and Advanced Materials, Faculty of Engineering and Natural Sciences Tampere University Tampere 33101 Finland; ^3^ CASUS‐Center for Advanced Systems Understanding Helmholtz‐Zentrum, Dresden‐Rossendorf e.V. (HZDR) Untermarkt 20 D‐02826 Görlitz Germany; ^4^ Engineering Research Center for Nanomaterials Henan University Kaifeng 475004 P. R. China

**Keywords:** defect engineering, ionic carbon nitrides, low‐temperature synthesis, nanostructural engineering, photocatalytic H_2_O_2_ generation

## Abstract

Solar‐driven oxygen reduction on ionic carbon nitride frameworks presents a compelling strategy for sustainable hydrogen peroxide (H_2_O_2_) production. Herein, a nanostructural engineering strategy is presented to tailor the morphology and defect chemistry of potassium poly(heptazine imide) (KPHI), enabling extended solar coverage and enhance photocatalytic performance. By incorporating NH_4_Cl into a molten KCl/LiCl eutectic medium, simultaneous nanoscale fragmentation of KPHI crystals and controlled introduction of cyano (–C≡N) defects are achieved. These molecular modifications induce n → π* electronic transitions, facilitate efficient charge separation, and accelerate oxygen reduction reaction kinetics. The optimal catalyst reaches an apparent quantum yield (AQY) of 49% at 410 nm and 5% at 525 nm without the need for cocatalysts, among the highest values reported for metal‐free photocatalyst systems. Transient absorption spectroscopy confirms preferential photoexcited electron localization at –C≡N sites, highlighting their key role in enhancing the charge carrier dynamics. Crucially, autogenous NH_3_ pressure is harnessed from NH_4_Cl decomposition to unlock a low‐temperature (500 °C) KPHI variant that delivers analogous performance to its counterpart produced at 600 °C, offering a more sustainable synthetic route. This study elucidates the structure‐activity relationship in ionic carbon nitrides and provides a generalizable approach for controlling their morphology and defect characteristics.

## Graphical abstract

A nanostructural refinement strategy is reported for KPHI photocatalysts using NH_4_Cl in a molten salt environment to induce coherent domain fragmentation and controlled cyano defect formation. This dual modification promotes n→π* transitions, enhances charge separation, and accelerates ORR kinetics, yielding exceptional AQY values of 49% (410 nm) and 5% (525 nm) without cocatalysts.

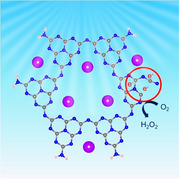

## 1 Introduction

With the exhaustion of natural resources and the rising energy demand, the extensive implementation of renewable energy sources has become essential.^[^ ^1^ ^]^ Hydrogen peroxide (H_2_O_2_) is a crucial chemical with widespread applications in industries such as food processing, paper bleaching, chemical synthesis, environmental protection, and sterilization of medical equipment.^[^ ^2^ ^]^ Global demand for H_2_O_2_ has been steadily increasing, reaching $3.5 billion last year and is projected to grow to $5 billion by the end of the decade.^[^ ^3^ ^]^ To date, 95% of the global H_2_O_2_ market demand comes from the complex anthraquinone oxidation process, which relies on large quantities of organic solvents and costly precious metal catalysts. From a carbon‐neutral perspective, exploring alternative production routes is highly desirable. In this context, H_2_O_2_ evolution via solar‐driven 2e^−^ oxygen reduction reaction (ORR) has emerged as one of the most promising solutions. This approach harnesses photogenerated charge carriers to facilitate redox reactions within an aqueous medium.^[^ ^4^, ^5^, ^6^, ^7^, ^8^ ^]^

Ionic carbon nitrides, such as metal poly(heptazine imides) (*M*PHI), have emerged as highly efficient photocatalysts for diverse reactions including H_2_ evolution,^[^ ^9^ ^]^ H_2_O oxidation,^[^ ^10^, ^11^ ^]^ H_2_O_2_ production,^[^ ^12^ ^]^ and organic transformations.^[^ ^13^ ^]^ The synthesis of *M*PHIs is primarily conducted in molten salt media, where factors such as the reaction temperature, the eutectic mixture composition, and the choice of N‐rich monomers dictate the condensation pathway and resultant *M*PHI structure. These catalysts have achieved high apparent quantum yields for their photocatalytic reactions in the UV range by fine‐tuning their critical photophysical pathways and the resultant chemical processes. Given these advancements, extending the absorption range of *M*PHI catalysts into the visible region is vital for boosting solar‐conversion efficiency and facilitating their transition to practical applications.

The π→ π* and n→ π* electronic transitions are mainly responsible for visible‐light absorption of carbon nitrides. Considering the n→ π* electronic transition, the lone pair electrons on sp^2^‐hybridized N atoms reside at a higher energy level than the π‐orbitals, which means they cannot be excited efficiently owing to their orthogonality to the π‐conjugated plane.^[^ ^14^ ^]^ Thus, to activate the n→π* electronic transition, restructuring the planar arrangement of heptazine units into a corrugated configuration is crucial.^[^ ^15^ ^]^ Substantial research efforts have focused in activating this transition with strategies including heteroatom doping,^[^ ^16^, ^17^, ^18^ ^]^ vacancy engineering,^[^ ^17^ ^]^ and molecular modification.^[^ ^19^ ^]^ Heteroatom doping disrupts the symmetry of heptazine units and introduces localized sites for charge carrier trapping but is often insufficient to fully enable the n→π* transition.^[^ ^20^ ^]^ Alternatively, introducing evenly distributed vacancies is a more effective strategy to enhance this transition and broaden the optical absorption profile. However, both synthetic strategies inherently increase the probability of charge carrier recombination, which adversely effects the photocatalytic performance.^[^ ^20^ ^]^ To mitigate this outcome, structural distortions can be induced using thermal treatment,^[^ ^21^ ^]^ infrared‐lattice vibrational coupling,^[^ ^22^ ^]^ exfoliation,^[^ ^23^ ^]^ or surface functionalization. For example, it has been reported that –C≡N and –NH_2_ groups can augment the photocatalytic activity of carbon nitrides.^[^ ^24^, ^25^ ^]^ Despite the reported success of these modification schemes, the impact of concurrent nanocrystal fragmentation and defect engineering on the photocatalytic performance of ionic carbon nitrides has yet to be reported.

In this work, we introduce a nanostructural refinement approach that enables precise control over the morphology and defect landscape in potassium PHI (KPHI). Through the incorporation of NH_4_Cl during KPHI synthesis, we induce targeted fragmentation of KPHI nanocrystals and promote the formation of –C≡N defects, which enhance n→ π* electronic transitions, improve separation efficiency, and ORR kinetics. Without any added cocatalyst, the optimized catalyst achieves an apparent quantum yield (AQY) of 49% at 410 nm and 5% at 525 nm, using glycerin as sacrificial reagent, outperforming the majority of previously reported carbon nitride‐based photocatalysts. Moreover, by leveraging the in‐situ generation of NH_3_ from NH_4_Cl decomposition, we enabled the synthesis of a KPHI material at a reduced temperature of 500 °C, which achieves comparable photocatalytic performance to the sample prepared at 600 °C, thus offering a more energy‐efficient and sustainable fabrication method.

## 2 Results and Discussion

### 2.1 Morphological and Structural Characterization

**Figure 1:**
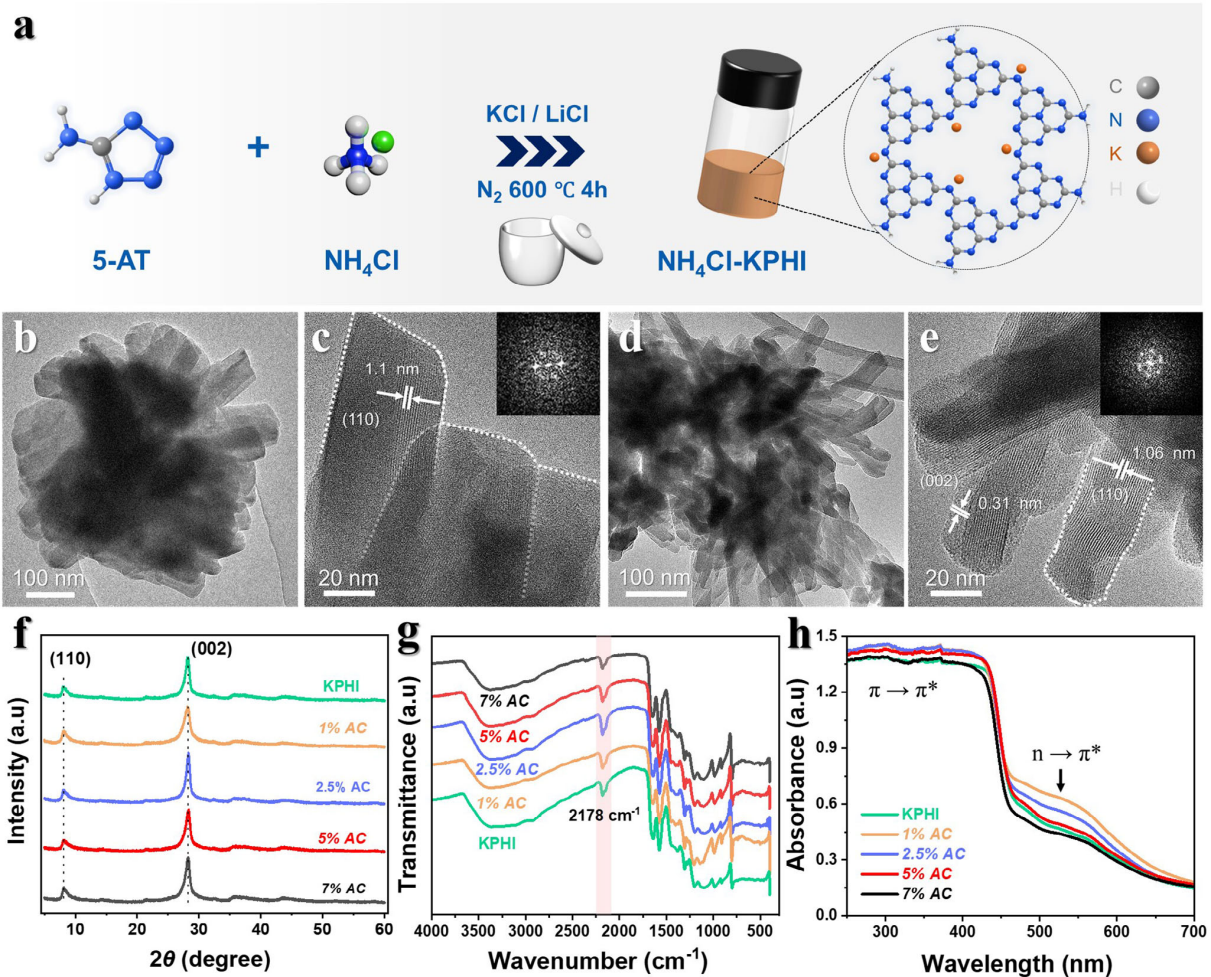
a) Schematic of the synthesis process for x%AC b, c) TEM images of KPHI and d, e) 2.5%AC. f) XRD patterns, g) FTIR spectra, and h) UV–vis absorbance spectra of KPHI and NH_4_Cl‐modified KPHI samples.

A schematic diagram depicting the modification of potassium poly(heptazine imide) (KPHI) with ammonium chloride (NH_4_Cl) is presented in **Figure** **1**a. This process involves annealing 5‐aminotetrazole with varying amounts of NH_4_Cl in the presence of a KCl/LiCl salt melt. NH_4_Cl was introduced in different concentrations: 1%, 2.5%, 5%, and 7% by weight relative to 2.5 g of 5‐aminotetrazole. The resulting materials were denoted as *x%AC*, where x indicates the NH_4_Cl concentration during the synthesis of KPHI. A mixture of 5‐aminotetrazole and appropriate amount of NH_4_Cl underwent ball milling at 25 Hz for 5 min to ensure uniform dispersion of precursor materials. Then, the blend was annealed at 600 °C for 4 h under a N_2_ atmosphere, forming a yellowish‐brown powder.

Transmission electron microscopy (TEM) imaging of all the samples shows structures featuring a rod‐like morphology with an average length of under 200 nm (Figure S1, Supporting Information). Increasing the concentration of NH_4_Cl resulted in a noticeable reduction in the nanocrystal size of the modified KPHI samples, indicating that the presence of NH_4_Cl affected the polymerization of KPHI. This phenomenon leads to the formation of smaller and more refined crystalline structures with a slight propensity to cluster together (Figure S1, Supporting Information). More specifically, the particles adopted a spherical shape and appeared denser in their aggregated state, particularly for the *5%AC* and *7%AC* samples. High‐resolution TEM images (Figure 1b–e) reveal that the large KPHI crystals were broken down into smaller, distorted rod‐like crystals in *2.5%AC* sample. A similar phenomenon was observed with oxamide‐modified KPHI, wherein the inclusion of oxamide during polymerization led to structural corrugation and the formation of defect sites.^[^ ^26^ ^]^ Both KPHI and a representative NH_4_Cl‐modified KPHI sample (2.5%*AC*) exhibit characteristic lattice spacings of 1.06 and 0.31 nm, corresponding to the (110) and (001) crystal planes. Thermogravimetric analysis (TGA) coupled with mass spectrometry (MS) revealed that, unlike the pristine KPHI precursor, the 2.5% *AC* precursor (5‐AT + KCl/LiCl + NH_4_Cl mixture) undergoes a distinct thermal profile due to NH_4_Cl (Figure S2, Supporting Information), demonstrating its impact during synthesis stage. The initial weight loss observed below 150 °C corresponds to the evaporation of physically adsorbed moisture on the precursor. A further mass decline occurring between 200–400 °C is linked to NH_3_ (m/z 17) release during condensation phase. In the 2.5%AC sample, the thermal decomposition of NH_4_Cl contributes substantially to NH_3_ evolution, which promotes the fragmentation of nanocrystals. Likewise, these findings suggest that the small amount of NH_4_Cl does not affect the melting behavior of the KCl/LiCl eutectic mixture, as it decomposes prior to the onset of salt melting. Scanning TEM‐energy dispersive spectroscopy (EDS) elemental mapping images verify the homogenous dispersion of C, N and K elements within the catalyst area (Figure S3, Supporting Information).

To investigate the structural impact of NH_4_Cl on the crystallinity of KPHI, X‐ray diffraction (XRD) measurements were performed (Figure 1f). For the unmodified KPHI, two distinct diffraction peaks were observed at 2*θ* = 8.1° and 28.1°, corresponding to the in‐plane ordering of the heptazine units and stacking mode of the KPHI lamellar structure, respectively. However, with the introduction of NH_4_Cl, particularly in the case of 2.5%AC, a noticeable shift of the (002) peak towards higher angles was observed (Figure S4, Supporting Information). This result is indicative of a more tightly stacked interlayer spacing caused by stronger π–π interactions between neighboring heptazine layers.^[^ ^27^ ^]^ Reducing the interlayer distance can improve charge transport within the stacked structure and lower the surface potential barrier, thereby enhancing photocatalytic activity.^[^ ^28^ ^]^

Additionally, the increased broadening of diffraction peaks at higher NH_4_Cl concentrations (> 5 wt. %) suggests a loss in long‐range crystalline order (Figure S4, Supporting Information). This observation aligns with TEM findings, which reveal that excessive NH_4_Cl disrupts the KPHI framework, leading to significant crystal fragmentation. We clarify that the term “crystal fragmentation” in this work denotes a reduction in the coherent crystalline domain size, rather than a loss of long‐range order. This is supported by the TEM observations (Figure S1, Supporting Information), which show that the originally larger KPHI crystallites become subdivided into smaller rod‐like domains during NH_4_Cl‐assisted synthesis. Because these smaller fragments maintain substantial internal order, this subdivision does not necessarily produce pronounced XRD peak broadening (Figure S4, Supporting Information). Fourier transform infrared spectra (FTIR) indicate that the NH_4_Cl‐modified KPHI samples possess similar functional groups to those of pristine KPHI, suggesting the local structure of the original polymeric framework has been preserved (Figure 1g). The prominent peak at 806 cm^−1^ corresponds to the out‐of‐plane bending mode of the heptazine ring, while the strong absorption bands between 1200 and 1650 cm^−1^ are usually attributed to the stretching modes of the heptazine heterocyclic ring. The signals at 985 cm^−1^ and 1119 cm^−1^ are due to the symmetric and asymmetric stretching of the NC_2_ bonds of K‐NC_2_ groups. Moreover, raising the NH_4_Cl content to 2.5 wt% led to a stronger signal for cyano group (–C≡N) at 2178 cm−^1^, suggesting the formation of more –C≡N groups during NH_4_Cl‐assisted polymerizatio process.^[^ ^29^ ^]^ It is well established that the photocatalytic performance is partially linked to the cyano group content in the catalysts, as these groups both alter the material's electronic structure and act as reservoirs to stabilize photoexcited charge carriers.^[^ ^29^ ^]^ The broad feature observed between 3000 cm^−1^ and 3500 cm^−1^ corresponds to N–H stretching vibrations. These peaks were slightly more prominent in the NH_4_Cl‐modified samples following increased crystal fragmentation. As the amount of NH_4_Cl is increased, the C/N ratio rises up to *2.5%AC* sample, while the K content decreases, as indicated in Table S1 (Supporting Information). One possible explanation is that the decomposition of NH_4_Cl releases H^+^ ions, which could partially substitute K^+^ ions in the PHI framework during pyrolysis, preserving the electroneutrality while reducing the measurable K content. Furthermore, decomposition byproducts like HCl could react with KCl to generate volatile species, lowering K incorporation into the final KPHI structure while retaining the integrity of the carbon nitride framework. NH_4_Cl‐modified KPHI samples showed smaller specific surface areas compared to pristine KPHI (Figure S5, Table S2, Supporting Information). This could be ascribed to the smaller particles created through fragmentation, which aggregated readily due to van der Waals forces or electrostatic interactions (as seen in microscopy analyses), thereby reducing the overall surface area available for N_2_ molecules during BET analysis.^[^ ^30^ ^]^

To explore the differences in chemical structure and composition among the synthesized samples, X‐ray photoelectron spectroscopy (XPS) was employed. The wide‐scan spectra (Figure S6a, Supporting Information) display only characteristic peaks for O 1s, N 1s, K 2p, and C 1s, confirming the absence of any impurities. In the high‐resolution C 1s spectra (Figure S6b, Supporting Information), the two peaks 284.6 eV and 288.5 eV are assigned to surface‐contaminated carbon (C–C) and sp^2^ hybridized carbon (N–C═N), respectively.^[^ ^31^ ^]^ Notably, *2.5%AC* shows a more pronounced peak at 286.3 eV, which corresponds to aminated carbons located at the edges of aromatic units (C–NH_x_). However, since –C≡N groups share a similar C1s binding energy with C–NH_x_ groups, the intensified signal could also be linked to the build‐up of more –C≡N groups, as further supported by FTIR analysis. A shift to higher binding energies for the N–C═N peak denotes modifications in the local bonding environment, attributed to crystal fragmentation, which influences the electronic structure of the C atoms. N 1s spectra can be deconvoluted into three peaks centered at 398.6, 400.1 and 401.0 eV, corresponding to sp^2^‐hybridized N atoms in the CN heterocyclic ring (C–N═C), tertiary nitrogen atoms from N–(C)_3_ groups in the heptazine units, and surface primary/secondary amine C–NH_x_ groups, respectively (Figure S6c, Supporting Information).^[^ ^32^ ^]^ To further verify the chemical structure of the as‐synthesized samples, solid‐state NMR spectroscopy was carried out. As depicted in Figure S7 (Supporting Information), both KPHI and 2.5%AC catalysts exhibited two main peaks centered at 156.6 and 165.1 ppm, corresponding to the chemical shifts of C_3N_ and C_2N_‐_NHx_ within the heptazine units, respectively. This reinforces the preserved structural integrity of the KPHI catalysts functionalized with NH_4_Cl. The augmented intensity of the C_2N_‐_NHx_ peak in 2.5%AC catalyst suggests a higher concentration of N‐containing groups introduced by the NH_4_Cl modification.

UV–vis diffuse reflectance spectroscopy (DRS) was utilized to assess whether the addition of NH_4_Cl modulated the optical and electronic properties of the photocatalysts. As illustrated in Figure 1h, the NH_4_Cl‐modified KPHI samples exhibited stronger absorption intensities compared to the pristine KPHI, especially for the n→π^*^ electronic transition. This is presumably due to their increased amount of –C≡N defect sites and crystal fragmentation which breaks the orthogonality between the lone electron pairs and the plane of π‐conjugation.^[^ ^15^ ^]^ Room‐temperature electron paramagnetic resonance (EPR) spectroscopy was then implemented to further investigate the nature of the –C≡N defect sites (Figure S8, Supporting Information). Lorentzian signals, with a g value of 2.003, are observed for both KPHI and *2.5%AC* catalysts. These are ascribed to the lone pair electrons from sp^2^‐hybridized N atoms in the π‐conjugated CN heptazine rings. Compared to KPHI, the signal in *2.5%AC* is noticeably stronger owing to a higher concentration of the delocalized electrons within its extended π‐conjugated framework.^[^ ^33^, ^34^ ^]^ These findings clearly demonstrate that NH_4_Cl modification in KPHI synthesis induced crystal fragmentation and increased the amount of –C≡N defect, improving visible light absorption and electron localization, respectively.

### 2.2 Photocatalytic H_2_O_2_ Performance

Photocatalytic H_2_O_2_ production was evaluated in an O_2_‐saturated aqueous glycerin solution (10% w/w) without the use of any cocatalyst, under a 410 nm LED irradiation. Considering its anticipated rise in output from the biofuel industry, glycerin is selected as a sacrificial electron donor instead of conventional trialkyl amines. During the 2e^−^ oxidation process, glycerol donates electrons to the photocatalyst and is converted into glycolic acid (GA) and formic acid (FA) (Figure S9, Supporting Information).^[^ ^23^ ^]^ A detailed quantification of these oxidation products lies beyond the scope of the present study and will be addressed in future work. Pristine KPHI sample generated 8.8 mM of H_2_O_2_ after one hour of irradiation. Incorporating NH_4_Cl into the precursor mixture notably improved the performance with *1%AC* catalyst producing 9.2 mM of H_2_O_2_.

**Figure 2:**
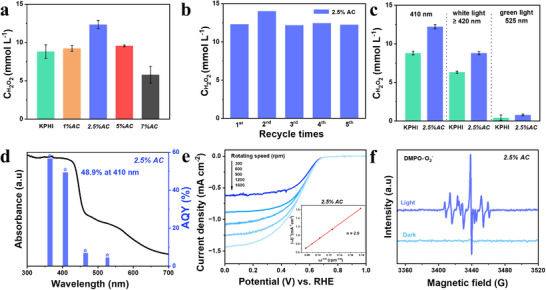
a) Effect of NH4Cl loading on the H_2_O_2_ evolution performance of KPHI. b) Recyclability tests for 2.5%AC in H2O2 production. c) Photocatalytic H2O2 evolution activities of KPHI and 2.5%AC using different excitation wavelengths. Reaction conditions for a), b) and c): photocatalyst, 5 mg; solvent, 2 mL of 10% w/w glycerin bubbled with O2 for 1 min before the reaction. d) UV–vis spectrum and AQY for 2.5%AC as a function of the irradiation wavelength. e) LSV curves of 2.5%AC tested by RDE (Inset: fitted K–L plot). f) EPR spectra of DMPO‐⋅O2− over *^2.5%AC^* ^.^

Further increasing the NH_4_Cl content to 2.5% resulted in an even higher photocatalytic activity, generating 12.3 mM of H_2_O_2_, a concentration 1.4 times greater than that of pristine KPHI (**Figure** **2**a). The catalytic process is purely light‐driven as no H_2_O_2_ is detected in the absence of light or catalysts (Figure S10, Supporting Information). Based on these observations, the catalytic performance is significantly improved due to crystal fragmentation caused by NH_4_Cl modification, along with defect formation, which improves electron localization. However, when the NH_4_Cl concentration exceeded 2.5 wt%, a slight drop in H_2_O_2_ production rate was observed due to reduced crystal sizes. The formation of smaller crystal domains introduces more grain boundaries, which can serve as charge recombination centers (Figure 1d). Additionally, the photocatalytic performance is further diminished due to intensified light scattering, which lowers the effective absorption of incoming photons.^[^ ^35^ ^]^ In the case of *2.5% AC*, the H_2_O_2_ generation rate declines after 1 hour of illumination, likely due to H_2_O_2_ decomposition at elevated temperatures caused by prolonged light exposure (Figure S11, Supporting Information). To further verify this, we conducted a control experiment using a 1 mM H_2_O_2_ aqueous solution with only 2.5%AC catalyst under continuous light irradiation, thereby excluding catalyst‐induced H_2_O_2_ decomposition. As shown in Figure S12 (Supporting Information), the H_2_O_2_ concentration remained essentially unchanged, confirming that no significant degradation occurred.

Furthermore, the 2.5% AC catalyst exhibited excellent stability and reusability, maintaining its H_2_O_2_ production performance at the initial level even after five consecutive runs (Figure 2b). The crystalline structure of the 2.5%AC catalyst was preserved after the recyclability tests (Figure S13a, Supporting Information); however, its physical appearance changed from yellowish‐brown to a dull white powder. In the FTIR spectrum of reused 2.5%AC catalyst, additional bands emerged at 3266 and 2178 cm^−1^, corresponding to –NH_x_ and –CH groups, respectively, while the metal–NC_2_ groups disappeared (Figure S13b, Supporting Information). Simultaneously, the (002) diffraction peak shifted to smaller angles, indicating an expansion of inter‐planar packing distance (Figure S13c, Supporting Information). These changes can be attributed to the coordination of protons generated during glycerin oxidation with the anionic sites on the catalyst surface, leading to the replacement of K ions, in agreement with our previous findings.^[^ ^9^ ^]^ This protonated state of the reused catalyst is further verified by STEM‐EDX mapping images, which show a negligible K signal (Figure S14, Supporting Information) in contrast to the original catalyst (Figure S3, Supporting Information). In addition, a long‐term test conducted in a larger setup for approximately 28 hours confirmed the excellent stability of the catalyst with continuous O_2_ purging, as evidenced by the nearly linear increase in H_2_O_2_ concentration throughout the experiment (Figure S15, Supporting Information). Post‐reaction characterization revealed properties consistent with those observed in the recyclability studies, including an expanded interplanar spacing and signatures of a protonated state in the FTIR spectrum (Figure S16, Supporting Information), indicating that the PHI framework remain intact while undergoing structural adaptation during operation.

The *2.5%AC* catalyst exhibits consistently higher H_2_O_2_ production across various wavelengths compared to pristine KPHI, indicating that NH_4_Cl treatment improves the performance of KPHI in visible‐light region (Figure 2c). The evolution of H_2_O_2_ is still detectable even at an excitation wavelength of 525 nm, albeit with low efficiency, suggesting that absorbed visible photons still contribute in driving the reaction. The *2.5%AC* catalyst reached an AQY 58.3%, 48.9%, 6.9%, and 4.5% under 365, 410, 465, and 525 nm excitation, respectively (Figure 2d). This exceptional performance ranks among the highest for visible‐light responsive carbon nitride‐based photocatalysts and even exceeds majority of recently reported materials under comparable experimental conditions (Table S3, Supporting Information).

The intrinsic electrocatalytic activity of the *2.5%AC* catalyst for oxygen reduction reaction (ORR) was compared to the pristine KPHI catalyst. Linear sweep voltammetry (LSV) measurements were performed in an O_2_‐saturated 0.2 M Na_2_SO_4_ solution using a rotating disk electrode (RDE). Within a comparable potential range, the *2.5%AC* catalyst exhibited higher cathodic current densities than KPHI, showing that the incorporation of NH_4_Cl facilitated an enhanced ORR rate (Figure 2e; Figure S17, Supporting Information). From the polarization curves recorded at different rotation speeds, the electron transfer number (*n*) was estimated using the Koutecky–Levich (K–L) equation. The calculated *n* values for KPHI and *2.5%AC* are 2.5 and 2.6, respectively, confirming that both catalysts predominantly favor the two‐electron ORR pathway over the four‐electron route leading to H_2_O formation. To further confirm these results, a series of control experiments were carried out under different gas atmospheres, as depicted in Figure S10 (Supporting Information). The H_2_O_2_ production rate was reduced under an atmosphere of ambient air (1.093 mM), and further reduced under a N_2_‐deoxygenated system (0.88 mM). These findings show that molecular oxygen (O_2_) is indispensable for the reaction to proceed. H_2_O_2_ can be produced either through a two‐step, 1e^−^ reduction process (O_2_ → ∙O_2−_ → H_2_O_2_) or a direct one‐step, 2e^−^ reduction (O_2_ → H_2_O_2_). To further elucidate the H_2_O_2_ formation mechanism in this system, EPR was employed to detect ^.^O_2_^−^. 5, 5‐dimethyl‐1‐pyrroline N‐oxide (DMPO) was used as a spin trap for ∙O_2−_. In the absence of light, no detectable EPR signal was observed, indicating the absence of ∙O_2−_ radicals under dark conditions. After 5 minutes of exposure to a 410 nm LED light, strong characteristic peaks corresponding to ∙O_2−_ radicals appeared (Figure 2f).

The free radical trapping experiments were also repeated using specific scavengers: AgNO_3_, tert‐butyl alcohol (TBA), and Na_2_S_2_O_3_. These reagents were carefully chosen to selectively neutralize electrons (e^−^), superoxide radicals (∙O_2−_), and hydroxyl radicals (∙OH), respectively (Figure S18, Supporting Information). The addition of AgNO_3_ to the reaction mixture significantly reduced the H_2_O_2_ production rate, underscoring the critical role of electrons (e^−^) in H_2_O_2_ generation. When TBA was introduced, the H_2_O_2_ yield decreased by approximately 50%, signifying that ∙OH radicals contribute to the reaction, which is confirmed by EPR measurement under light irradiation in Figure S19 (Supporting Information). Detectable H_2_O_2_ formation is obtained even in the absence of glycerin, indicating that photoexcited holes can oxidize water to generate ∙OH radicals (Figure S18, Supporting Information). This process simultaneously releases protons from H_2_O, which can subsequently facilitate the hydrogenation steps involved in the 2e^−^ ORR. The most notable effect was observed with Na_2_S_2_O_3_, which nearly eliminated the H_2_O_2_ production, suggesting that ∙O_2−_ radicals are crucial intermediates in the formation of H_2_O_2_. This implies that ∙O_2−_ radicals are essential intermediates and *2.5%AC* follows two consecutive 1e^–^ ORRs in H_2_O_2_ production.

### 2.3 Optical and Photo(electro)Chemical Characterizations

**Figure 3:**
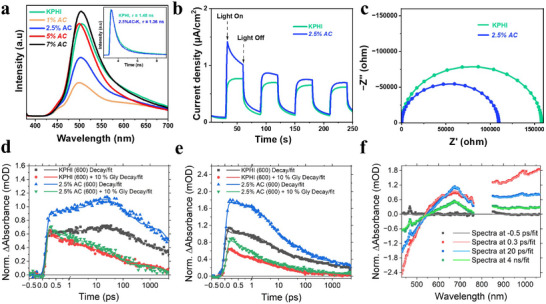
a) Room temperature steady‐state PL emission spectra of KPHI and *x%AC* with an excitation wavelength of λ = 365 nm (inset: solid‐state time‐resolved PL decay of KPHI and *2.5%AC*). b) Transient photocurrent (λ = 410 nm) for KPHI and *2.5%AC* in 0.2 m Na_2_SO_4_ aqueous solution. c) Electrochemical impedance spectroscopy (EIS) Nyquist plots. d, e) The normalized fs‐TAS decays of *2.5%AC* (600) and KPHI (600) monitored at 675 nm (d) and 990 nm (e). The timescale is shown in linear scale until 1 ps and logarithmic scale for longer decay times. f) The normalized fs‐TAS spectra of *2.5%AC* (600). The excitation was at 400 nm with 0.1 mJ cm^−2^ excitation energy density for all samples.

Based on the Mott‐Schottky analysis (Figure S20, Supporting Information), the flat‐band potential positions were determined to be −0.01 V and −0.07 V (versus RHE) for KPHI and *2.5%AC*, respectively (Figure S21, Supporting Information). The observed cathodic shift implies that *2.5%AC* possesses a stronger thermodynamic driving force to facilitate the O_2_‐to‐H_2_O_2_ reduction process more effectively. Next, steady‐state and time‐resolved photoluminescence (PL) spectroscopy measurements were performed to compare the photogenerated charge separation efficiencies of the catalysts. The *2.5%AC* catalyst had a lower fluorescence intensity compared to pristine KPHI in **Figure** **3**a. This is due to the presence of the electron‐withdrawing cyano groups, which act as electron traps, thus enhancing charge separation efficiency and suppressing the radiative recombination pathway.^[^ ^36^ ^]^ This is further supported by the shorter average carrier lifetime of *2.5%AC* (1.36 ns) relative to KPHI (1.48 ns) (Figure 3a, inset). At higher loadings (5 wt% = 2.26 ns; 7 wt% = 2.08 ns), the mean lifetimes increase again, which we attribute to the formation of excess defects that introduce trap states and promote trap‐assisted recombination (Figure S22, Table S4, Supporting Information). This trend is fully consistent with the observed decline in photocatalytic activity, confirming that excessive NH4Cl addition is detrimental to performance. The charge transfer properties of the catalysts were evaluated through photoelectrochemical measurements. The photocurrent response profiles revealed that the *2.5%AC* catalyst displayed higher current densities than KPHI, which indicates more efficient charge separation and transport (Figure 3b). Moreover, the electrochemical impedance spectroscopy (EIS) revealed a smaller semicircular in the Nyquist plot for the *2.5%AC* catalyst, reflecting its lower interfacial charge transfer resistance compared to KPHI (Figure 3c). These results, combined with improved light absorption and suppressed charge recombination due to defect formation, demonstrate that the enhanced charge carrier separation and migration capabilities are key factors driving the superior ORR performance of the *2.5%AC* catalyst.

The dynamics of carbon nitrides are derived from excitons that dissociate into polaron pairs on adjacent polymeric sheets or recombine geminately. The polarons can diffuse perpendicularly through adjacent polymeric sheets before recombination occurs between the original or different polaron pairs.^[^ ^37^ ^]^ The timescales of geminate and bimolecular recombination occur in the fs–ps and ns–µs timescales, respectively.^[^ ^38^ ^]^ Femtosecond transient absorption spectroscopy (fs‐TAS) measurements were performed for KPHI and *2.5%AC* photocatalysts with and without 10 % glycerin to understand how the cyano defects affect the photophysics for H_2_O_2_ production. The 2D transient spectra (Figure S23, Supporting Information) show a ground state bleach (GSB) at wavelengths below 510 nm for KPHI. The GSB edge shifts to approximately 550 nm for *2.5%AC* due to the increased strength of the n → π* transition. Immediately after excitation, they show similar positive transient absorption features due to excited state absorption (ESA) from photogenerated carriers,^[^ ^28^, ^39^, ^40^ ^]^ with a localized transient absorption band centered at 675 nm and a more intense flat transient absorption in the NIR range. In the presence of glycerin, both KPHI and *2.5%AC* indicate a blue shift of the GSB signal and a decrease in the intensity and lifetime of the positive ESA features.

The data was fit with a three‐exponential model (see Supporting Information for details) and the transient decays and spectra at different decay times are shown in Figure 3d–f; Figure S24 (Supporting Information) after normalizing the responses to the steady‐state absorbance at the excitation wavelength (400 nm, Figure S25, Supporting Information). The normalized *2.5%AC* transient signal has a stronger intensity than KPHI, indicative of a larger exciton concentration after excitation. Interestingly, at fast to intermediate delay times between 1–20 ps, the positive ESA band at 675 nm increases in intensity for both KPHI and *2.5%AC* with exponential lifetimes of 6.4 and 5.3 ps, respectively, whereas in the IR range the signal decays with the same lifetime (Table S5, Supporting Information). After 20 ps, the signal in both ranges decays biexponentially with a faster component with lifetime of 169 and 113 ps for KPHI and *2.5%AC*, respectively, and a long‐living component with unresolved lifetime (> 5 ns) for both materials. In the presence of glycerin, the TAS signals in the whole monitoring range decay more rapidly with lifetimes of 1.2 ps, 36 ps, and 3.46 ns for KPHI and 1.2 ps, 57 ps, and 4.49 ns for *2.5%AC*, without a growing or very long‐living component visible for either material after excitation.

To better understand the differences in dynamics in the presence and absence of glycerin, the decay associated spectra (DAS) representing the exponential components of the multiexponential fitting are presented in Figure S26 (Supporting Information). The DAS are similar between KPHI and *2.5%AC*. Each decay component is shown in separate graphs for a more rigorous analysis (Figure S27, Supporting Information). In the fastest decay component, the growth of the signal at 675 nm disappears in the presence of glycerin. The growth is assigned to the formation of hole polarons due to exciton dissociation via electron trapping, whereas the hole polarons are scavenged from the excitons by glycerin. In the NIR region, a decay that is assigned to the excitons is observed simultaneously with the hole polaron formation. The second decay component shows very similar spectral changes for all samples with the exception that *2.5%AC* retains its larger signal intensity. In the third decay component, the long‐living signal of hole polarons after electron trapping are clearly visible for both KPHI and *2.5%AC*, with the signal intensity being two times larger for *2.5%AC* indicating a much larger concentration of residual hole polarons at longer delay times. The additional cyano defects in *2.5%AC* may increase trapping and explain the larger TAS signal intensity when compared to KPHI, but since the decay dynamics are very similar between the two materials, we cannot prove this. We believe that most of the TAS intensity increase is from a higher exciton concentration originating possibly from the reduced domain sizes in *2.5%AC*. Thus, the fs‐TAS spectra and decays display an increased contribution of hole polarons and trapped electrons for *2.5%AC* compared to KPHI. However, the hydrogen peroxide formation presumably occurs at a longer timescale than the measurement window of our fs‐TAS instrument

**Figure 4:**
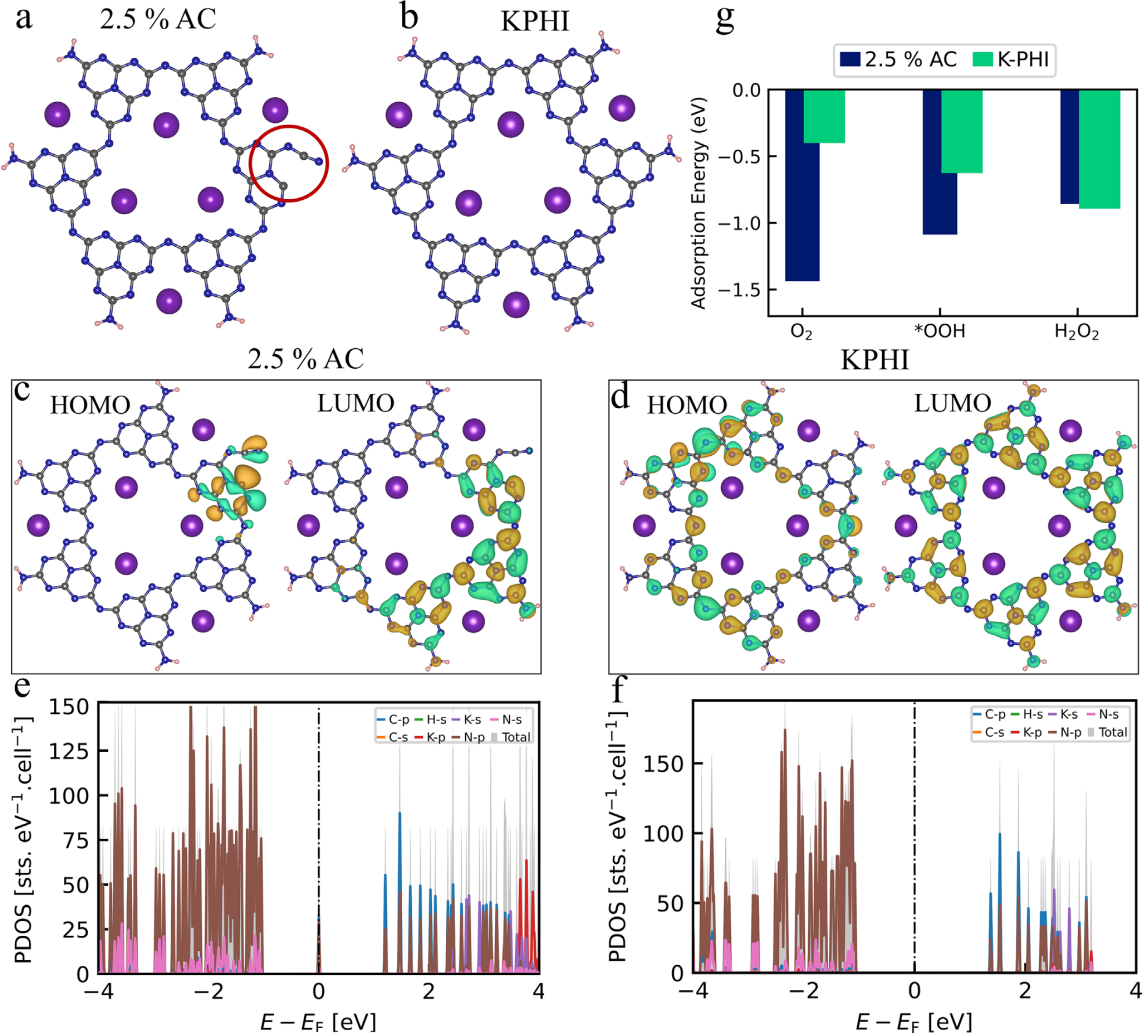
a, b) Structure, c, d) projected density of state, e, f) electronic structure of the optimized HOMO and LUMO for 2.5%AC and KPHI, respectively. g) Adsorption energy profile of O_2_, OOH, and H_2_O_2_ on 2.5% AC (blue) and KPHI (green).

To further elucidate the influence of defect sites in photocatalytic H_2_O_2_ evolution, we performed density functional theory (DFT) calculations linking structural modifications to catalytic performance. **Figure** **4**a and b compare the pristine KPHI structure with its *2.5%AC* counterpart, the latter incorporating a defective heptazine unit functionalized with an in‐plane cyano group. For consistency, molecular flakes of identical dimensions were employed to eliminate size‐dependent electronic effects and isolate the role of the cyano defect. The optimized *2.5%AC* structure reveals that the highest occupied molecular orbital (HOMO) and lowest unoccupied molecular orbital (LUMO) are strongly localized around the cyano moieties (Figure 4c), consistent with their radical character. This orbital confinement introduces additional adsorption sites for reactant molecules. By contrast, pristine KPHI exhibits more delocalized electron density (Figure 4d), limiting its ability to effectively anchor reactive intermediates. Projected density of states (PDOS) analysis provides further insights: in both systems, C 2p and N 2p orbitals dominate the conduction band, while the valence band is primarily derived from N 2p states (Figure 4e,f). Notably, the bandgap of 2.5%AC undergoes a negligible change; however, the presence of a free‐electron state near the Fermi level suggests enhanced electronic conductivity. In agreement with the orbital distribution, PDOS confirms that *2.5%AC* possesses an elevated electron density around the Fermi level, thereby strengthening interactions with adsorbates and promoting charge transfer.

We then evaluated the adsorption energetics of key species along the H_2_O_2_ formation pathway: O_2_, OOH, and H_2_O_2_ (Figure 4g). Remarkably, O_2_ adsorption is much more favorable on *2.5%AC* (‐1.44 eV), underscoring its enhanced capacity to initiate the catalytic cycle. Adsorption of intermediates likewise yields more negative energies on *2.5%AC* (‐1.08 eV), confirming improved stabilization. These effects stem from the increased free‐electron density introduced by cyano defects, which promote stronger binding of reactants and intermediates. Taken together, these findings demonstrate that NH_4_Cl modification, through the introduction of cyano defects, restructures the electronic landscape of KPHI. This defect engineering strategy enhances conductivity, enriches surface active sites, and establishes a more favorable pathway for photocatalytic H_2_O_2_ production.

### 2.4 Low‐Temperature Design of NH_4_Cl‐Modified KPHI

As discussed earlier, the introduction of NH_4_Cl during KPHI synthesis led to the evolution of NH_3_ gas, which played a key role in inducing defect formation and promoting crystal fragmentation. Prior studies have demonstrated that well‐crystallized carbon nitride nanocrystals can be obtained under solvothermal conditions at a relatively low temperature of 400 °C, using NH_4_Cl as a nitriding agent and CCl_4_ as a carbon source.^[^ ^41^, ^42^, ^43^ ^]^ Inspired by the potential of achieving KPHI formation at reduced temperatures by leveraging autogenous NH_3_ pressure generated from NH_4_Cl decomposition, the solid‐state synthesis of KPHI and *2.5%AC* at 400 °C and 500 °C was explored to evaluate the impact of thermal conditions on structural integrity and photocatalytic performance (denoted as catalyst@temperature).

**Figure 5:**
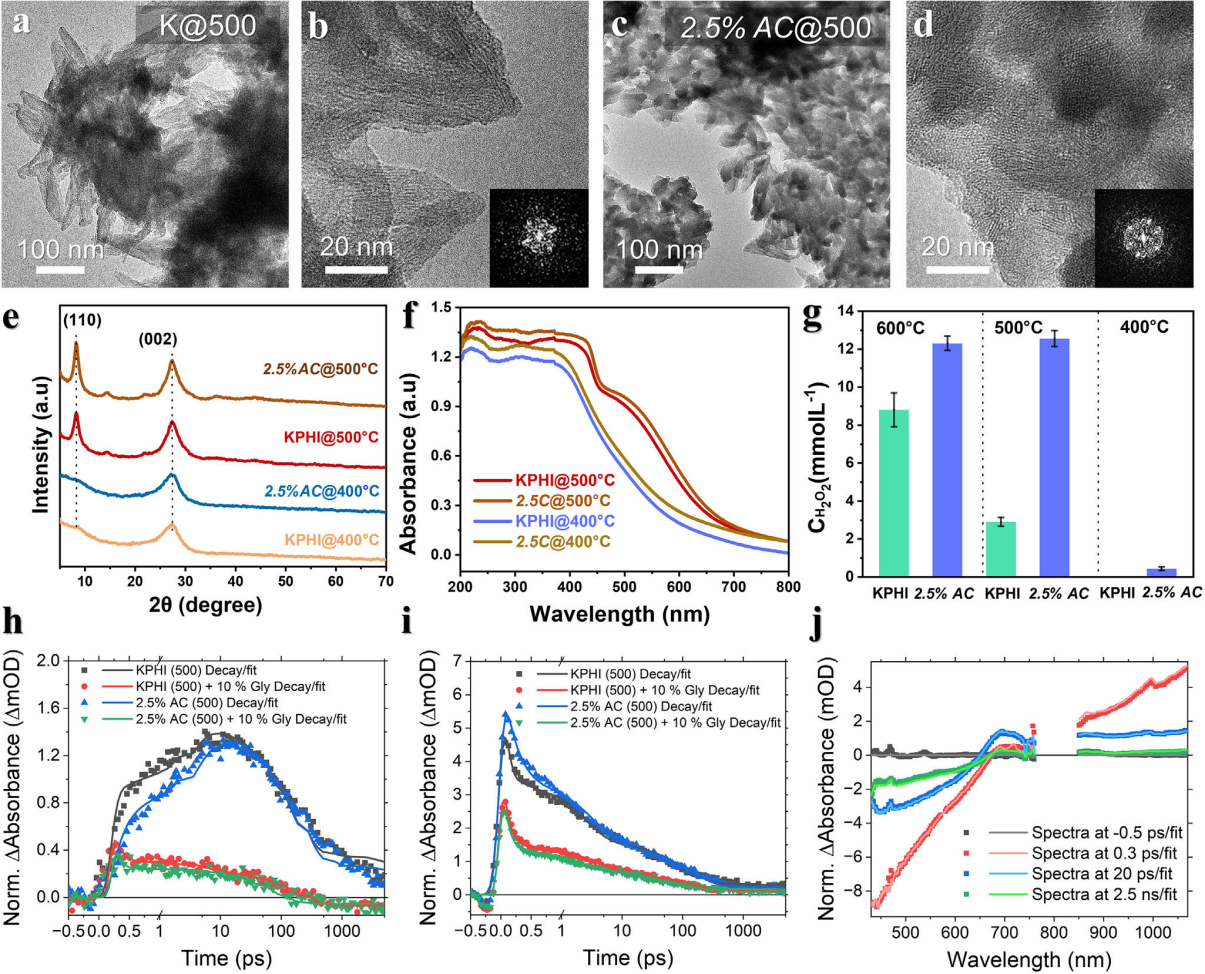
TEM images of a, b) KPHI and c, d) *2.5%AC* synthesized at 500 °C, e) XRD patterns, f) UV–vis absorbance spectra, and g) photocatalytic H_2_O_2_ performance of KPHI and *2.5%AC* catalysts prepared at different temperatures. h, i) The normalized fs‐TAS decays of *2.5%AC*@500 and KPHI@500 monitored at 715 nm (h) and 990 nm (i). The timescale is shown in linear scale until 1 ps and logarithmic scale for longer decay times. j) The normalized fs‐TAS spectra of *2.5%AC* (500). The spectra are cut off around 800 nm due to the fundamental laser pulse. The excitation was at 400 nm with 0.04 mJ cm^−2^ excitation energy density for all samples.

TEM images show that pristine KPHI@500 formed pointed nanorods with an average length of about 100 nm and a rough surface texture (**Figure** **5**a,b). In contrast, *2.5%AC*@500 catalyst exhibited smaller rod‐like nanocrystals compared to both KPHI@600 and KPHI@500, composed of densely packed crystalline domains (Figure 5c,d). *2.5%AC*@500 displays more pronounced diffraction peaks (Figure 5e) along with weaker ‐NH_x_ group signals compared to KPHI@500, signifying a higher degree of crystallinity, as further supported by the microscopic analyses. However, when the synthesis was made at 400 °C, both catalysts developed into large amorphous nanosheets with lower absorption, suggesting this temperature is insufficient to drive the growth of well‐defined crystalline domains (Figure 5e; Figure S28, Supporting Information). *2.5%AC*@500 and KPHI@500 had similar UV–vis absorption profiles showing both π→ π* and n→ π* electronic transitions with the former possessing a slightly higher intensity in the visible region. In comparison, the absorption profiles of *2.5%AC*@400 and KPHI@400 were blue shifted and lacking profound absorption edges (Figure 5f). The release of NH_3_ gas from NH_4_Cl decomposition triggers a localized pressure buildup around the carbon nitride precursors, which can lower the activation energy required for polymerization. To further validate this effect beyond the TGA‐MS analysis, we synthesized *2.5%AC@500* in a tube furnace to examine whether the NH_4_Cl decomposition products re‐condense during the reaction. As shown in Figure S29 (Supporting Information), a white powder was deposited at the cooler end of the tube. XRD analysis identified this deposit as NH_4_Cl in the case of *2.5%AC@500*, while no such condensation was observed for KPHI synthesized under identical conditions at 500 °C. These results confirm that NH_4_Cl acts as a transient promoter, releasing gaseous species that drive polymerization at lower temperature and subsequently re‐condensing outside the reaction zone.

Photocatalytic H_2_O_2_ production experiments revealed that KPHI@400 exhibited nearly no activity, whereas *2.5%AC* @400 produced a small amount of H_2_O_2_ (0.43 mM) (Figure 5g). Interestingly, the *2.5%AC* @500 achieved the same H_2_O_2_ evolution rate as the catalyst synthesized at 600 °C, while KPHI@500 presented a 65% decrease in activity in comparison to KPHI@600. These results highlight that the addition of NH_4_Cl promotes effective polymerization at lower temperatures, enabling the synthesis of highly active KPHI catalysts without the need for high thermal input, thereby offering a more energy‐efficient route to functional photocatalysts.

As before, fs‐TAS measurements were performed for KPHI@500 and *2.5%AC*@500 to understand the difference in the photophysics compared to those of the samples synthesized at 600 °C (Figure S30, Supporting Information). The 2D plots showed a red shifted GSB up to 650 nm due to the shifts in the steady‐state absorption of the samples, while the positive ESA in the visible range is centered at 715 nm.^[^ ^44^ ^]^ Notably, the increase in the intensity of ESA features for both KPHI@500 and *2.5%AC*@500 are slower compared to their counterparts produced at 600 °C. This can be due to the more distorted structure of the samples produced at lower temperatures, which slows the intralayer exciton diffusion and subsequent interlayer charge separation.^[^ ^45^ ^]^ In the presence of glycerin, both samples displayed a more pronounced decrease in intensity of the positive ESA features than the materials prepared at 600 °C, and there was an associated red‐shift in the GSB signal.

After fitting with a three‐exponential model (see SI for details), the transient spectra and decays shown in Figure 5h–j and Figure S31 (Supporting Information) were normalized to their steady‐state absorptions at 400 nm (Figure S25, Supporting Information). At these lower synthesis temperatures, the normalized ESA signal intensities at 715 nm were comparable for both samples though *2.5%AC*@500 had a slightly smaller intensity immediately after excitation than KPHI@500. Similarly, to the samples produced at 600 °C, the ESA band at 715 nm increases in intensity within the first 20 ps with comparable exponential lifetimes of 2.2 ps and 3.5 ps for KPHI@500 and *2.5%AC*@500 respectively. In the same timescale, the NIR transient signal displayed only decay (Table S5, Supporting Information). In the same timescale, the rise time at 715 nm was slower while the decay at 990 nm was faster for *2.5%AC*@500 compared to KPHI@500. After 20 ps, the signal in both ranges decayed biexponentially with similar lifetimes of 107 and 133 ps for KPHI@500 and *2.5%AC*@500, respectively, and a long‐living component with unresolved lifetime (>5 ns) for both materials. In the presence of glycerin, there was a loss in intensity for the GSB and ESA, and the associated lifetimes in the intermediate timescale reduced most significantly for *2.5%AC*@500 to 1.1 ps and 53 ps.

To better understand the differences with glycerin, the DAS were compared between the samples in Figure S32 (Supporting Information). The DAS are similar for KPHI@500 and *2.5%AC*@500 in the absence of glycerin. In the presence of glycerin, the dynamics of KPHI@500 become slightly slower, whereas the decay dynamics of *2.5%AC*@500 became faster. A more rigorous analysis was made in Figure S33 (Supporting Information) with a separate graph for each decay component. The *2.5%AC*@500 sample has increased intensity for the GSB in all decay components compared to KPHI @500 which suggests an increased concentration of charge carriers after photoexcitation, although ESA intensities were comparable in all but the last component. Like for the samples at 600 °C, the fastest decay component corresponds to the formation of hole polarons due to exciton dissociation via electron trapping, most visible as the disappearance of the 715 nm ESA band in the presence of glycerin. This was also accompanied by a reduction in intensity in the NIR region, corresponding to exciton dissociation, and a large decrease in GSB intensity. The second and third decay components are spectrally very similar, indicating strongly that they originate from the same decay process that occurs biexponentially, presumably from intra‐ and intersheet recombination of the hole polarons with trapped electrons.

Overall, compared to the samples at 600 °C, the differences in ESA intensity between KPHI@500 and *2.5%AC*@500 are less pronounced, which suggests a similar concentration of polaron holes from excitonic dissociation due to electron trapping, possibly due to the reduced domain sizes which does not allow for efficient intersheet charge separation. However, the marked difference in photocatalytic activity between the samples implies that the relevant photoreactions occur outside the monitoring timescales (ns‐µs). To gain further insight into the enhanced photocatalytic performance of *2.5%AC*@500, the differences in their steady‐state fluorescence intensities were examined (Figure S34, Supporting Information). The slightly lower fluorescence intensity of *2.5%AC*@500 in comparison to its KPHI counterpart suggests suppressed charge carrier recombination, due to the trapping of electrons at the cyano defects. This observation is consistent with the transient photocurrent (Figure S35, Supporting Information), which show that *2.5%AC*@500 exhibits a much higher photocurrent response and lower charge transfer resistance. Together, these findings indicate that the activity enhancement in *2.5%AC*@500 is primarily due to more efficient interfacial charge separation and transfer.

## 3 Conclusion

In this work, we demonstrate that the addition of NH_4_Cl into the synthesis of KPHI significantly boosts its photocatalytic performance for solar‐driven H_2_O_2_ production. By varying the NH_4_Cl content during KPHI synthesis, we achieved controlled fragmentation of KPHI nanocrystals, resulting in reduced particle size, and the introduction of –C≡N groups. These structural modifications enhance visible‐light absorption through activation of the n→π* transition. Among the synthesized materials, *2.5%AC* catalyst displayed the highest H_2_O_2_ yield with an AQY of 49% and 5% at an excitation wavelength of 410 nm and 525 nm, respectively. Comprehensive characterizations confirmed that this outstanding performance can be attributed to a combination of factors such as increased structural distortion, a higher density of defect sites that facilitate electron trapping, and improved interlayer stacking that enhances charge separation and mobility. Electrochemical assessment further established that NH_4_Cl modification enhances the ORR kinetics. Additionally, we found that the presence of NH_4_Cl allows the synthesis of highly active KPHI photocatalysts at a reduced temperature of 500 °C, providing a more energy‐efficient alternative to conventional high‐temperature methods. Overall, this work underscores the dual role of NH_4_Cl in promoting structural optimization and defect engineering, and introduces a scalable route to produce efficient, durable ionic carbon nitrides for sustainable H_2_O_2_ production under visible light. Looking ahead, translation of these findings toward large‐scale application will require engineering solutions that complement our materials design approach. In particular, thin‐film or microstructured continuous‐flow photoreactors could be employed to maximize light penetration and mass transfer, coupled with efficient O_2_ delivery and catalyst immobilization to simplify product separation. Moreover, incorporating in‐situ product removal strategies can help suppress H_2_O_2_ decomposition and increase effective yields. Finally, practical implementation demands attention to durability and safety via long‐term testing under realistic photon fluxes and the use of H_2_O_2_‐compatible reactor materials.

## Conflict of Interest

The authors declare no conflict of interest.

## Supporting information



Supporting Information

## Data Availability

The data that support the findings of this study are available from the corresponding author upon reasonable request.
