# Supplementary material for: NH3‐Guided Low‐Temperature Nanostructural Refinement Boosts Visible‐Light‐Driven H2O2 Synthesis in Ionic Carbon Nitrides
Source: Adv Mater. 2025 Nov 16;38(7):e10585. doi: 10.1002/adma.202510585 (PMC12862718; doi:10.1002/adma.202510585)
Supplement: Supplementary file 1 — Supporting Information [file ADMA-38-e10585-s001.docx]

Supporting Information

NH_3_-Guided Low-Temperature Nanostructural Refinement Enables Superior Solar-Driven H_2_O_2_ Synthesis in Ionic Carbon Nitrides

*Jaya Bharti, ^1^ Jokotadeola Odutola,^2^* *Zahra Hajiahmadi,^3^ Karlo Nolkemper,^1, 3^ Zhihong Tian,^4^Haijian Tong,^1^ Vitaliy Shvalagin, ^1^ Thomas D. Kühne,^3^ Tero-Petri Ruoko,^2^ and Christian Mark Pelicano^1, *^*

^1^ Department of Colloid Chemistry, Max Planck Institute of Colloids and Interfaces, Potsdam 14476, Germany

^2^ Chemistry and Advanced Materials, Faculty of Engineering and Natural Sciences, Tampere University, Tampere, 33101 Finland

^3^CASUS - Center for Advanced Systems Understanding, Helmholtz-Zentrum

Dresden-Rossendorf e.V. (HZDR), Untermarkt 20, D-02826 Görlitz, Germany

^4^Engineering Research Center for Nanomaterials, Henan University, Kaifeng 475004, P. R. China

Corresponding author: christianmark.pelicano@mpikg.mpg.de

**Experimental Section**

**Synthesis of KPHI**

Following the methodology from our previous publication, potassium poly(heptazine imide) (KPHI) was synthesized. The process started by placing 2.5 g of dried 5-Amino-1H-tetrazole monohydrate and 12.5 g of a KCl/LiCl eutectic mixture (in a 0.55/0.45 ratio) into a steel ball mill vessel, which was then ground at a frequency of 25 Hz for 5 minutes. The resulting white powder was transferred into a lidded porcelain crucible and subjected to a heating process. The temperature in the furnace was gradually increased to 600°C at a rate of 2.3°C per minute under a continuous nitrogen flow (4 L/min), where it was held for 4 hours. After the furnace was allowed to cool naturally to room temperature, the product was moved to a beaker with deionized water and stirred at room temperature overnight. Finally, the mixture was vacuum filtered, extensively washed with water via centrifugation, and dried overnight in a vacuum oven at 60°C.

**Synthesis of *x%AC***

To obtain *x% AC* with varying NH_4_Cl content, NH_4_Cl was introduced at 1-7 wt% relative to the weight of 5-Amino-1H-tetrazole into the KCl-LiCl eutectic mixture. The synthesis of *x% AC* followed a procedure similar to that of KPHI, with this minor modification in the salt template. The weight ratio of NH_4_Cl to 5-Amino-1H-tetrazole was adjusted between 1 and 7. After this adjustment, the subsequent steps mirrored those used for KPHI synthesis. The final products were labeled as *x% AC*, where x represents 1, 2.5, 5, and 7, respectively.

**Characterization**

X-ray powder diffraction (XRD) patterns were obtained using a Rigaku Smart Lab instrument (Japan, Cu Kα radiation, 0.154 nm) with a generator voltage of 40 kV and a current of 50 mA, scanning at a speed of 2°/min over a range of 5° to 80°. X-ray photoelectron spectroscopy (XPS) was performed with a Thermo Fisher Scientific ESCALAB 250Xi system. Fourier transform infrared (FTIR) spectroscopy was carried out using a Thermo Scientific Nicolet iD5 spectrometer equipped with an attenuated total reflection (ATR) sampling accessory. Thermogravimetric analysis (TGA) was conducted on a Shimadzu TGA-60H, operating from 25 to 900°C under synthetic air flow (50 mL/min) with a heating rate of 10°C/min, using an alumina crucible.

Inductively coupled plasma mass spectrometry (ICP-MS) measurements were performed with a PerkinElmer ICP-OES Optima 8000. Elemental combustion analysis (EA) was executed using a vario MICRO cube CHNOS analyzer (Elementar Analysensysteme GmbH). Nitrogen adsorption-desorption isotherms and pore size distributions were evaluated at 77 K using a Quantachrome Quadrasorb SI instrument. Before analysis, the samples were degassed at 150 °C under a vacuum of 0.5 Torr for 15 hours. Specific surface areas were determined from the adsorption branch data (P/P0 < 0.3) using the Brunauer-Emmett-Teller (BET) method.

Sample morphologies were examined with a Zeiss LEO 1550-Gemini scanning electron microscope (SEM) equipped with an Oxford Instruments X-MAX energy-dispersive X-ray (EDX) detector. Transmission electron microscopy (TEM) images were captured using a JEOL JEM F200 and a JEOL JEM-ARM 200F with double Cs correction, operating at 80 kV. The setup included a cold-field emission gun and a high-angle silicon drift EDX detector (Jeol JED 2300) with a solid angle of up to 0.98 steradians and a detection area of 100 mm^2^.

The optical properties and charge carrier dynamics of the samples were analyzed using UV-vis diffuse reflectance spectroscopy (UV-vis DRS) on a Shimadzu UV-2600 instrument, electron paramagnetic resonance (EPR) with a Bruker EMXnano, and steady-state photoluminescence (PL) spectroscopy on a Jasco FP-8300 fluorescence spectrometer with a 365 nm excitation wavelength. Time-resolved PL (TRPL) measurements were recorded on a FluoTime 250 spectrometer (PicoQuant) equipped with a PDL 800-D picosecond pulsed diode laser. The average lifetime (τ_ave_) was calculated using the formula:

(τ_ave_) = (A_1_ τ_1_^2^ + A_2_ τ_2_^2^ + A_3_ τ_3_^2^) /(A_1_ τ_1_ + A_2_ τ_2_ + A_3_ τ_3_)

**Electrochemical Measurements**

The rotating ring-disk electrode (RRDE) and rotating disk electrode (RDE) methods were employed to determine the number of electrons transferred (n) during the oxygen reduction reaction (ORR). These electrochemical measurements utilized a three-electrode setup, comprising an Ag/AgCl reference electrode, a Pt ring counter electrode, and an O₂-saturated 0.2 M Na_2_SO_4_ electrolyte. The RRDE was operated at varying rotational speeds, ranging from 400 to 1600 rpm, and the applied potential was scanned between 0 and 1.0 V vs. the reversible hydrogen electrode (RHE). To prepare the catalyst ink, 6 mg each of the catalyst and black carbon were ground thoroughly and dispersed in a mixture of pure water (490 μL), isopropanol (490 μL), and 5 wt.% Nafion solution (20 μL). The suspension was sonicated for 1 hour, and 11 μL of the resulting ink was deposited onto the RRDE electrode and dried at room temperature, achieving a catalyst loading of approximately 0.28 mg cm^-2^. For RRDE experiments, linear sweep voltammetry (LSV) was conducted on the disk electrode while a constant potential (e.g., 1.2 V vs. RHE) was maintained on the ring electrode. Hydrogen peroxide (H_2_O_2_) generated on the disk diffused to the ring electrode, where it was detected. The number of electrons transferred (n) was calculated using the formula:

n = Id / (Id + Ir / N)

where Ir is the ring current, Id is the disk current, and N (collection efficiency) is 0.37. In RDE measurements, the Koutecky–Levich (K–L) equation was applied to compute n:

1 / I = 1 / IK + 1 / (0.620 n F A D_0_^2/3^ ω^1/2^ ν^-1/6^ C_O_^2^)

where I is the measured steady-state current (mA cm^-2^), IK is the kinetic current, F is the Faraday constant (96458 C mol^-1^), A is the geometric area of the working electrode, D₀ is the oxygen diffusion coefficient (cm^2^ s^-1^), ω is the angular rotation speed, ν is the kinematic viscosity of the electrolyte (cm^2^ s^-1^), and C_o_^2^ is the saturated oxygen concentration (mol cm^-3^).

**Photoelectrochemical measurements**

All photoelectrochemical experiments were performed using a three-electrode setup, with a platinum wire as the counter electrode and an Ag/AgCl electrode serving as the reference. The measurements were conducted using a Gamry Interface 1010E potentiostat. For the preparation of the working electrode, fluorine-doped tin oxide (FTO) glass substrates (3 x 1 cm) were sequentially cleaned with detergent, distilled water, and ethanol for 15 minutes each to eliminate organic contaminants. A catalyst ink was prepared by combining 5 mg of photocatalyst powder, 0.5 mL of water, and 20 μL of 5 wt.% Nafion solution, followed by sonication for 30 minutes. Subsequently, 50 μL of the resulting slurry was applied to the FTO substrate, dried at 60^°^C, and then heated to 120^°^C for 1 hour to enhance adhesion. The measured potentials were standardized to the reversible hydrogen electrode (RHE) scale using the equation:

*E_RHE_* = *E* *_Ag/AgCl_* + 0.059 × pH + 0.197

**Transient photocurrent response (TPR)**

The photocurrent response was recorded at 0 V against the reference electrode in a 0.5 M aqueous Na_2_SO_4_ solution under illumination with a white LED (100 mW cm^-2^), utilizing a Gamry Interface 1010Epotentiostat.

**Electrochemical impedance spectroscopy (EIS)**

EIS measurements were conducted using the same electrodes as described earlier, over a frequency range of 10 kHz to 1 Hz. The data were analyzed and fitted to a complete semicircle using Z-View software.

**Mott–Schottky Measurements**

Mott Schottky analysis was performed using a Biologic MPG-2 system across various frequencies, employing the same electrodes as outlined in previous sections.

**Radical Quenching Experiments**

A sample vial was prepared by adding 5 mg of 2.5%AC powder to 2 mL of water containing 10% glycerin and a 10 mM sacrificial reagent: AgNO_3_ for e^-^, NaS_2_O_3_ for ·O_2_^-^, or t-butyl alcohol (TBA) for ·OH. The mixture was purged with O₂ gas for 1 minute before being irradiated under stirring with two 50 W LED lamps (λ = 410 nm) for 1 hour. The produced H_2_O_2_ was measured via spectrophotometry.

**EPR Experiment**

The presence of ·O_2_^-^ radicals in the reaction system was detected using electron paramagnetic resonance (EPR) with a spin-trapping reagent, 5,5-Dimethyl-1-pyrroline N-oxide (DMPO). Measurements were carried out in a H_2_O/MeOH mixture (1:4, 500 μL) containing 1 mg of the sample and 0.1 mmol DMPO, irradiated with a 410 nm LED.

**Evaluation of Photocatalytic Performance**

For performance testing, 5 mg of the catalyst was dispersed in 2 mL of an aqueous glycerin solution at a specified concentration. O_2_ gas was bubbled through the mixture for 1 minute, and the reactor was irradiated under stirring with two 50 W LED lamps (λ = 410 nm) for 1 hour. Post-irradiation, the suspension was centrifuged at 10,000 rpm for 10 minutes to separate the catalyst. The amount of H_2_O_2_ produced was quantified spectrophotometrically using the titanium oxalate method. Specifically, a 10 g/L solution of K_2_[TiO(C_2_O_4_)_2_]·2H_2_O was prepared by dissolving the compound in 450 mL of water and 50 mL of H₂SO₄ to prevent precipitation. Then, 1.5 mL of this reagent was mixed with 0.5 mL of the supernatant from the photocatalytic test. The solutions, diluted as needed, were analyzed using UV–vis spectrometry at 400 nm. A calibration curve was constructed with H_2_O_2_ standards ranging from 0 to 10 mmol/L, displaying a linear response (R² = 0.9999).

**Apparent Quantum Yield (AQY) Estimation**

The AQY was determined using monochromatic LEDs at 365, 410, 465, and 525 wavelengths. It was calculated using the equation:

AQY (%) = (2 × R_product_ × N_A_ × h*c) / (I*A*λ) × 100

where R_product_ is the rate of H_2_O_2_ production (mol s^-1^) after the first hour of reaction, N_A_ is Avogadro's constant (6.022 × 10^23^ mol^-1^), h is Planck’s constant (6.626 × 10^-34^ J s^-1^), c is the speed of light (3 × 10^8^ m s^-1^), A is the illuminated area (cm^2^), I is the light intensity (W cm^-2^), and λ is the wavelength of the monochromatic light (nm).

**Femtosecond transient absorption spectroscopy (fs-TAS) and steady state absorbance spectroscopy**

For the measurements, 5 mg of each of the powder samples@600 were prepared in 5 mL of milliQ water and 10 mg of each of the powder samples@500 were prepared in 5 mL of milliQ water. Then, the samples were sonicated for an hour in a bath sonicator to improve their optical quality for the spectroscopic measurements. Due to the differences in structure and size, the samples prepared at 500°C were centrifuged at 750 rpm for 10 minutes, while the samples prepared at 600°C were centrifuged at 2500 rpm for 10 minutes to achieve stable dispersions in water. The supernatants were used as stock solutions and diluted appropriately to make a total of eight samples: including (a) a control sample of each in milliQ water alone, and (b) a sample of each in 10 % glycerin. The glycerin acts as a sacrificial electron and proton donor. All measurements were made in quartz cuvettes of path length 2 mm under atmospheric conditions of oxygen, temperature, and pressure.

The femtosecond transient absorption spectroscopy (fs-TAS) measurements were made using pump-probe spectroscopy. The fundamental ultrashort laser pulses were generated at 800 nm from the Ti:Sapphire laser (Libra F, Coherent Inc.), of which 90 % was coupled to an optical parametric amplifier (OPA) from Topas C, Light Conversion Ltd to produce the pump beam (for 400 nm, 0.1 mJ/ cm^2^ for the samples@600 and 0.04 mJ/ cm^2^ for the samples@500). The remaining 10 % was passed through a delay line for a transient response up to 5 ns. A water cuvette was used to generate the probe beam in the visible range (430 – 760 nm) while a sapphire crystal was used to generate the probe beam in the near infrared range (850-1070 nm). The transient absorption responses of the visible range probe beam were measured using an ExciPro TA spectrometer (CDP, Inc.), with a Si CCD diode array for the visible range and an InGaAs diode array for the near infrared range. The time resolution of the instrument was 100 fs.

The measured data was corrected against group velocity dispersion and fitted globally to estimate characteristic time constant and the spectra of intermediate states. Typically, an initial fit model was a sum of exponentials as shown as in equation (**6**) below:

$$\boldsymbol{\Delta}\boldsymbol{A} \left( \boldsymbol{\lambda}\mathbf{,}\boldsymbol{t} \right)\mathbf{=}\boldsymbol{A}_{\mathbf{0}} \left( \boldsymbol{\lambda} \right)\mathbf{+}\sum_{\mathbf{i=1}}^{\mathbf{N}} \boldsymbol{A}_{\mathbf{i}} \left( \boldsymbol{\lambda} \right)\boldsymbol{exp}^{\frac{\mathbf{-}\boldsymbol{t}}{\boldsymbol{\tau}_{\mathbf{i}}}}$$

where ΔA (λ,t) was the transient absorption at a specific wavelength and time; A_0_ (λ) was the response independent of the delay time; A_i_ (λ) were the pre-exponential factors or decay associated spectra (DAS); and τ_i_ were the time constants for each decay component. The fs-TAS spectra also displayed scattering of the pump pulse by the particles in suspension. Thus, it was necessary for some of the samples to add an additional exponent in the multiexponential fitting to account for the scattering. Since these additional exponents were faster than the instrument resolution of 100 fs, they are not included in the discussion.

The steady state absorbance of these solutions was determined using a Shimadzu UV–3600 series spectrophotometer in the range of 250 to 800 nm. These were used to normalize the fs-TAS decays based on their absorbance at 400 nm. The poly dispersity indexes (PDI) and the zeta-average (nm) of the samples were measured by dynamic light scattering using the Malvern Panalytical Zetasizer nano ZS. The PDI values are from 0 to 1 with smaller values showing a more monodisperse sample. The zeta-averaged sizes which were measured were taken as the diameter of the samples in suspension. The samples were centrifuged with a Hettich® Universal 320R centrifuge.

**Computational Details**

All calculations were performed using density functional theory (DFT) with the r2SCAN-3c ^1^ meta-generalised gradient approximation (meta-GGA) functional, as implemented in the ORCA 6.1.0 code ^2^. Frontier molecular orbital and projected density of states (PDOS) were analayzed by Multiwfn 3.8 ^3^. The adsorption energy (ΔE_ads_) of adsorbate adsorption on the surface is defined as:

ΔE_ads_ =E(*adsorbate)-E(*)-E(adsorbate)

Where E(*aadsorbate) and E(*) denote the total energies of the surface with and without the adsorbate, respectively, and E(adsorbate) is the energy of an isolated adsorbate (O_2_, OOH, H_2_O_2_). By this definition, a negative adsorption energy indicates an exothermic process and thus a thermodynamically stable adsorption system. All structural models and isosurface visualizations were generated using VESTA ^4^.


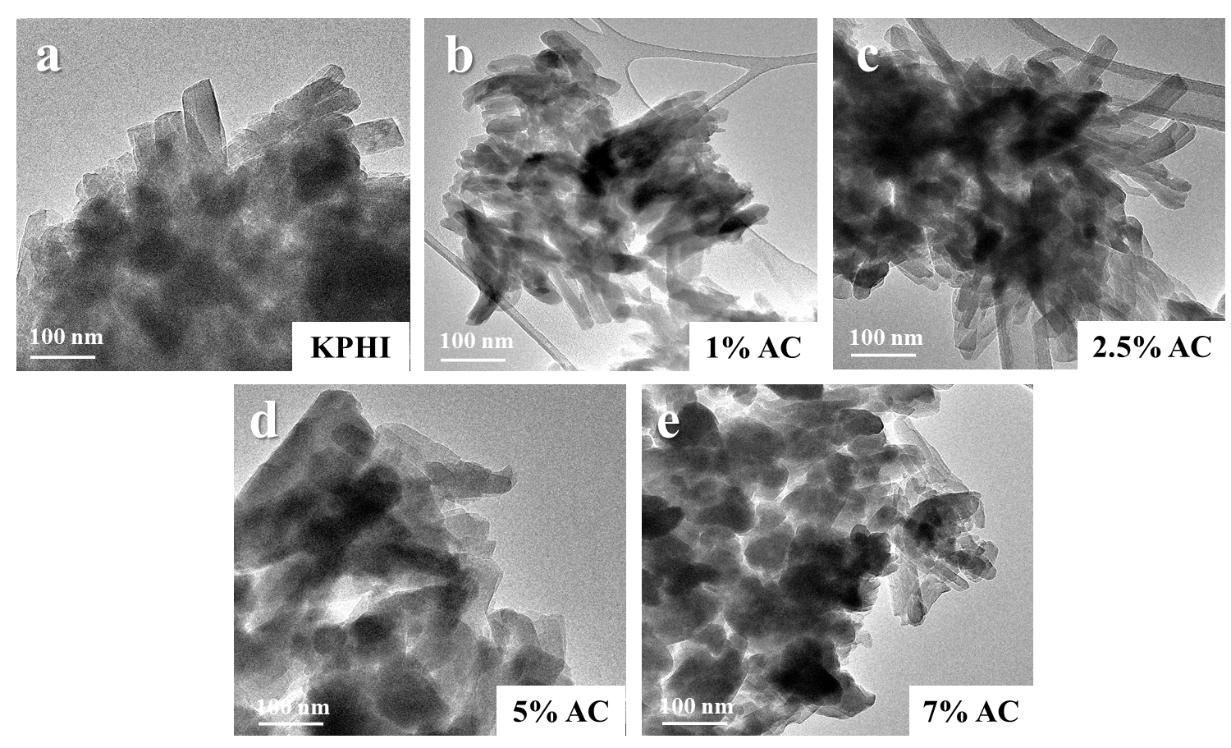


**Figure S1.** TEM images of KPHI and *x*%*AC* samples.


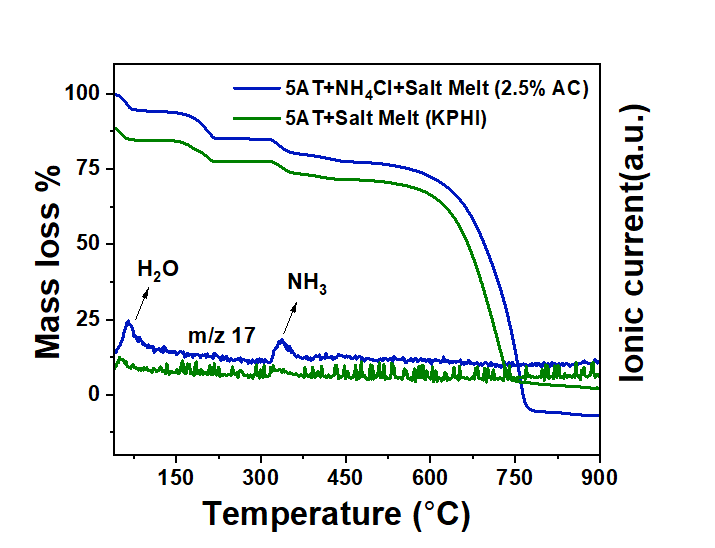


**Figure S2.** TGMS curves of KPHI and *2.5% AC* recorded under inert atmosphere (10 K min^-1^).


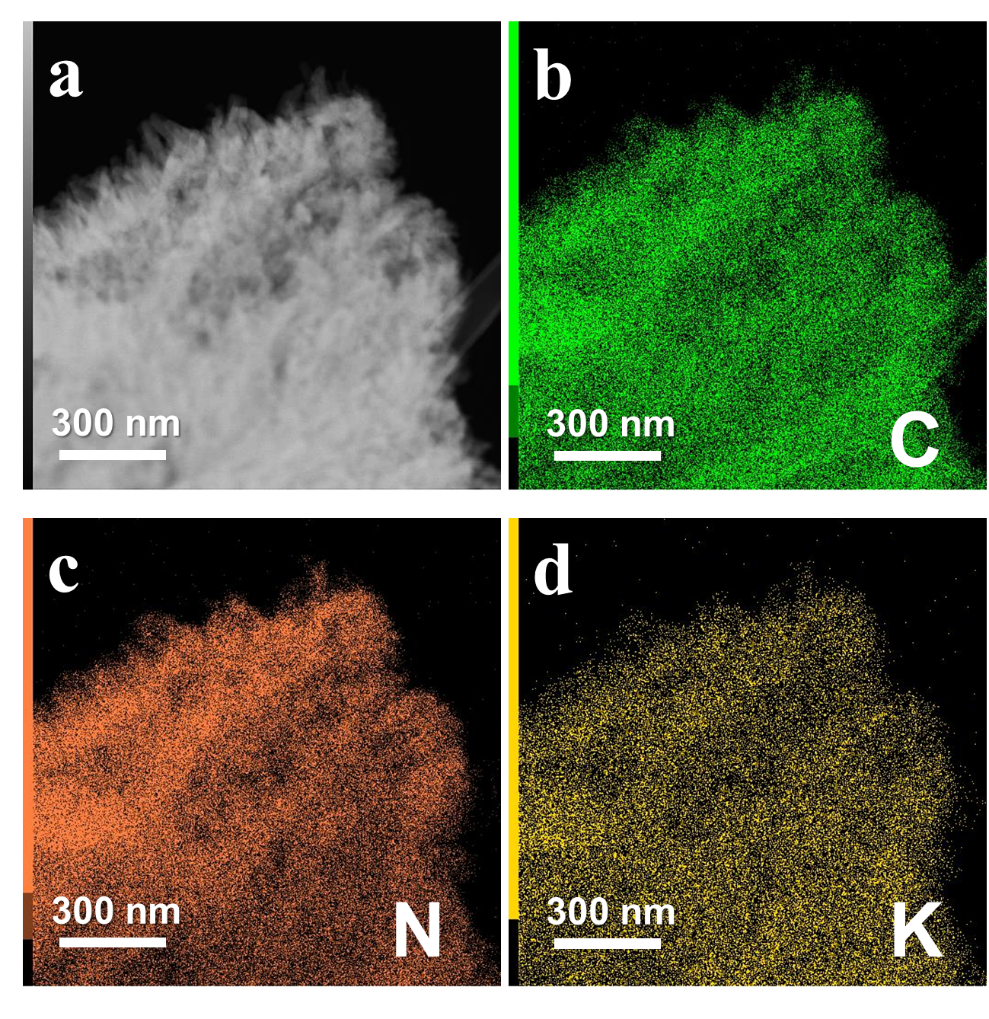


**Figure S3.** **(a)** TEM image of *2.5%AC* and **(b–d)** its corresponding elemental mapping images.


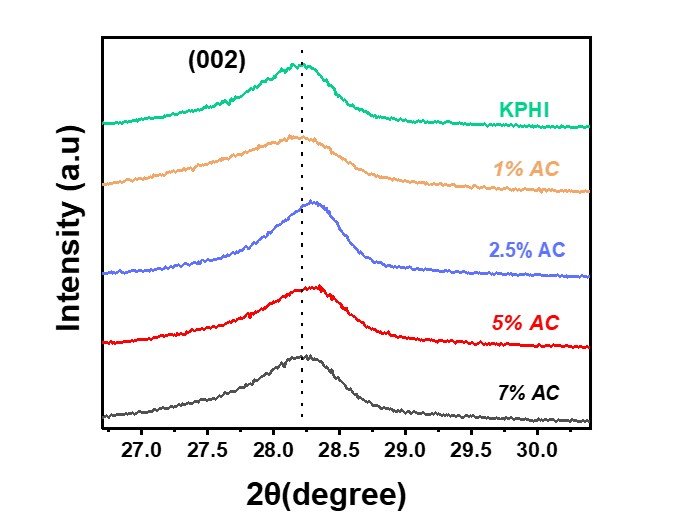


**Figure S4.** XRD patterns of KPHI and *x*%*AC* samples.

**Table** **S1.** The relative ratios of C, N and O elementals in all samples determined by elemental analysis and the total amount of alkali metals in all catalysts via ICP.

| **Samples** | **C (%)** | **N (%)** | **C/N** | **K (%)** | **Li (%)** |
| --- | --- | --- | --- | --- | --- |
| KPHI | 27.3 | 45.9 | 0.59 | 12.4 | 0.20 |
| *1%AC* | 27.3 | 45.2 | 0.60 | 10.4 | 0.28 |
| *2.5%AC* | 27.7 | 44.9 | 0.62 | 10.8 | 0.18 |
| *5%AC* | 27.3 | 45.4 | 0.60 | 11.5 | 0.25 |
| *7%AC* | 27.4 | 45.7 | 0.60 | 9.8 | 0.13 |


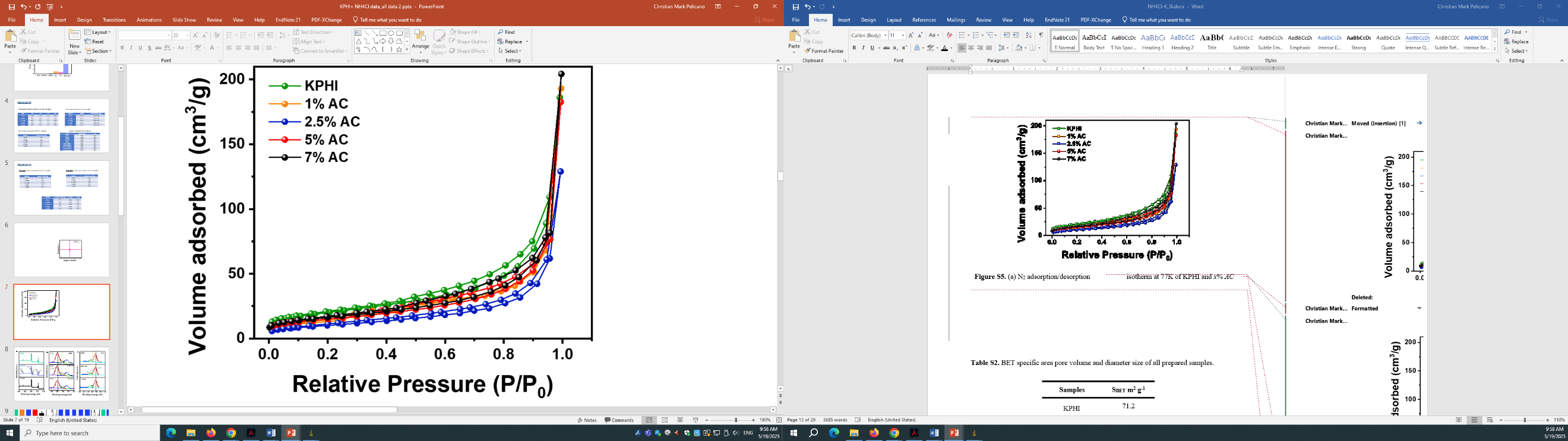


**Figure S5.** **(a)** N_2_ adsorption/desorption isotherm at 77K of KPHI and *x*% *AC* catalysts.

**Table** **S2.** BET specific area pore volume and diameter size of all prepared samples.

| **Samples** | **S_BET_ m^2^ g^-1^** |
| --- | --- |
| KPHI | 71.2 |
| *1%AC* | 46.5 |
| *2.5%AC* | 36.6 |
| *5%AC* | 53.1 |
| *7%AC* | 57.7 |


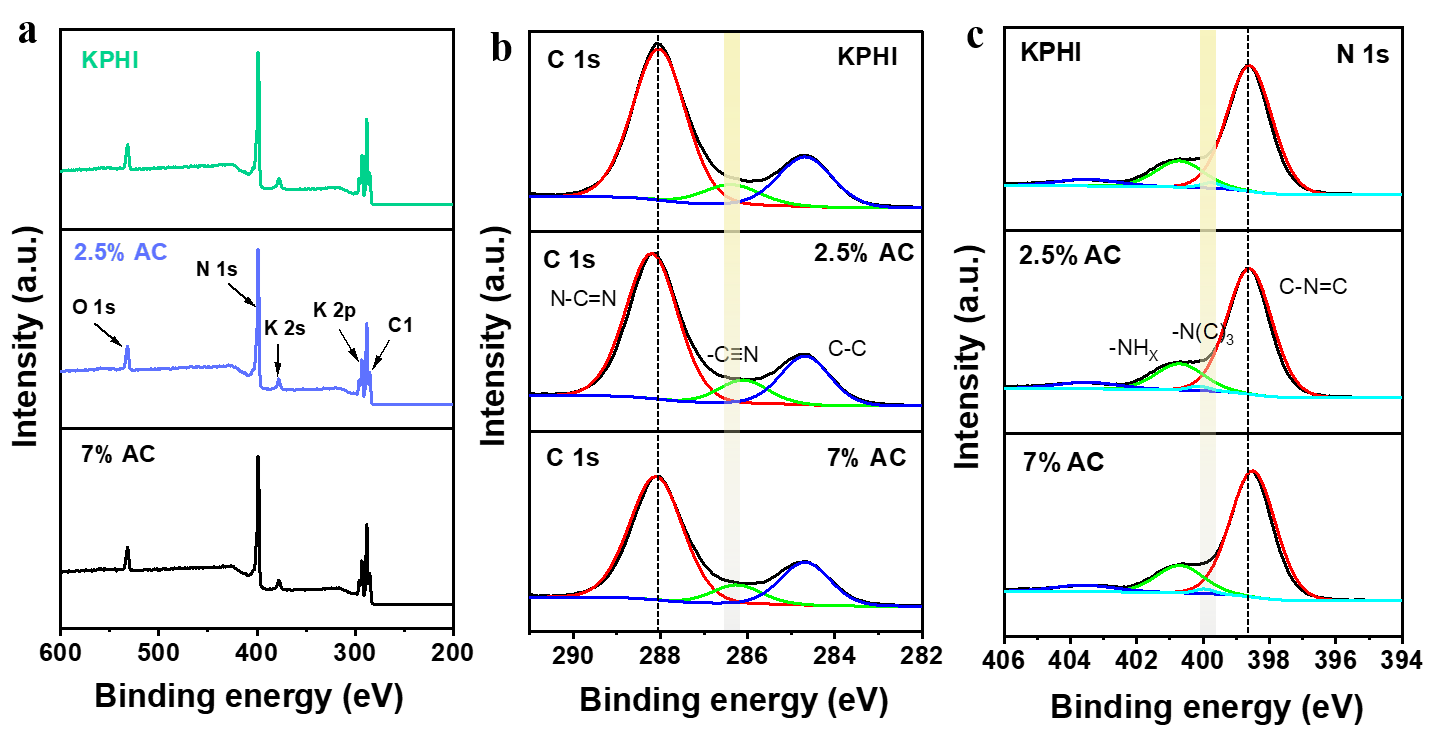


**Figure S6.** **(a)** XPS full spectra, **(b)** high-resolution C 1s and **(c)** N 1s of KPHI and *x*%AC.


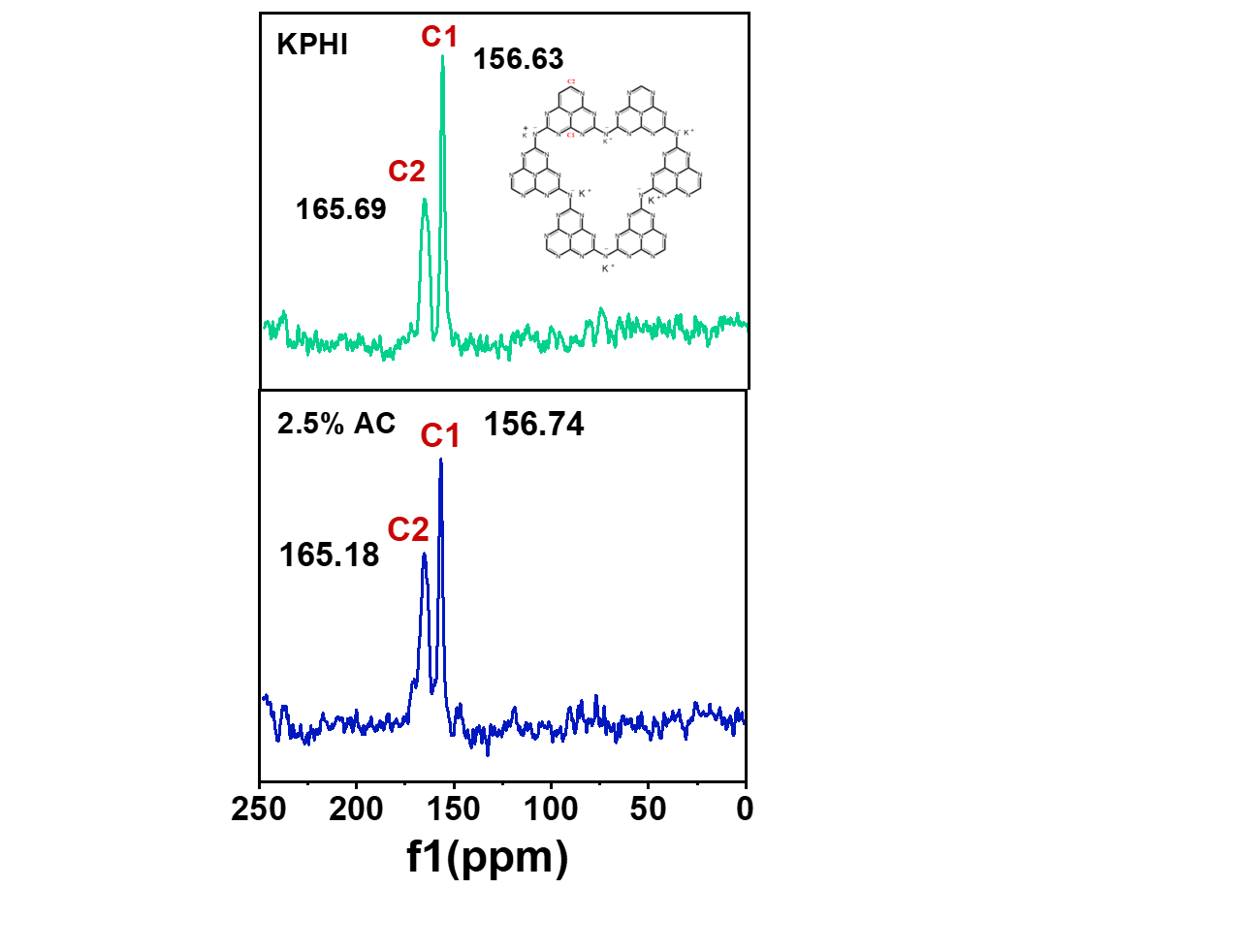


**Figure S7.** Solid-state ^13^C NMR spectrum of KPHI and *2.5*%*AC.*


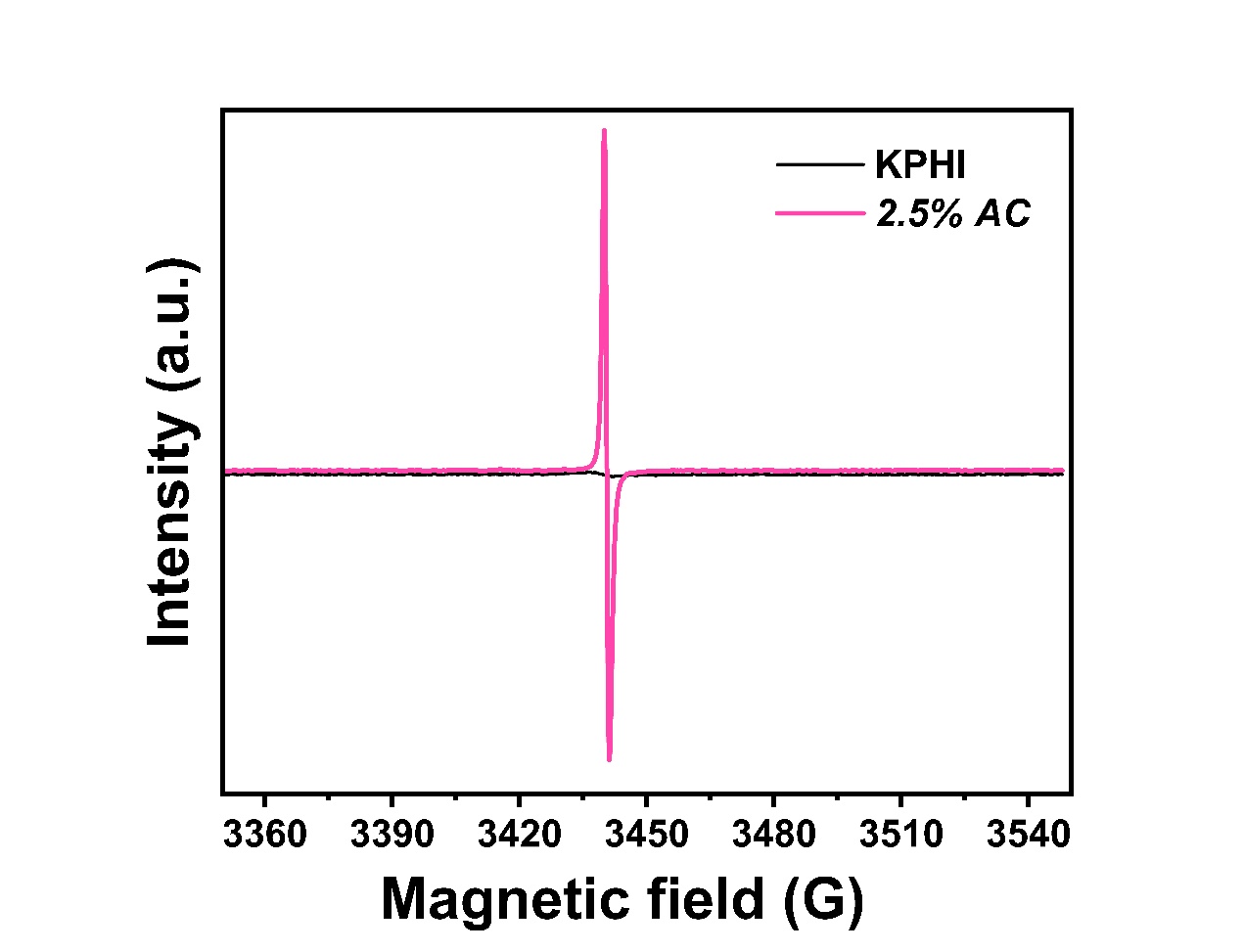


**Figure S8.** Solid-state EPR spectrum of KPHI and *2.5*%*AC*.


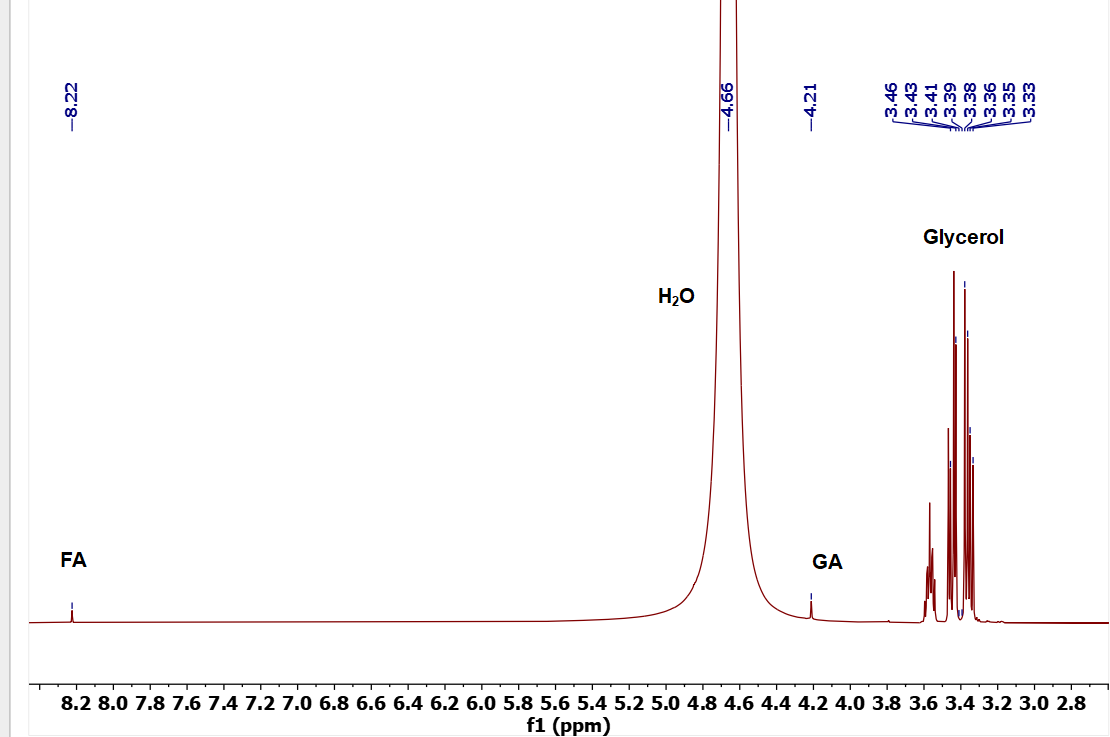
**Figure S9.** ^1^H NMR of the aqueous glycerin solution (with 2.5% AC catalyst) after 1hr of light irradition.


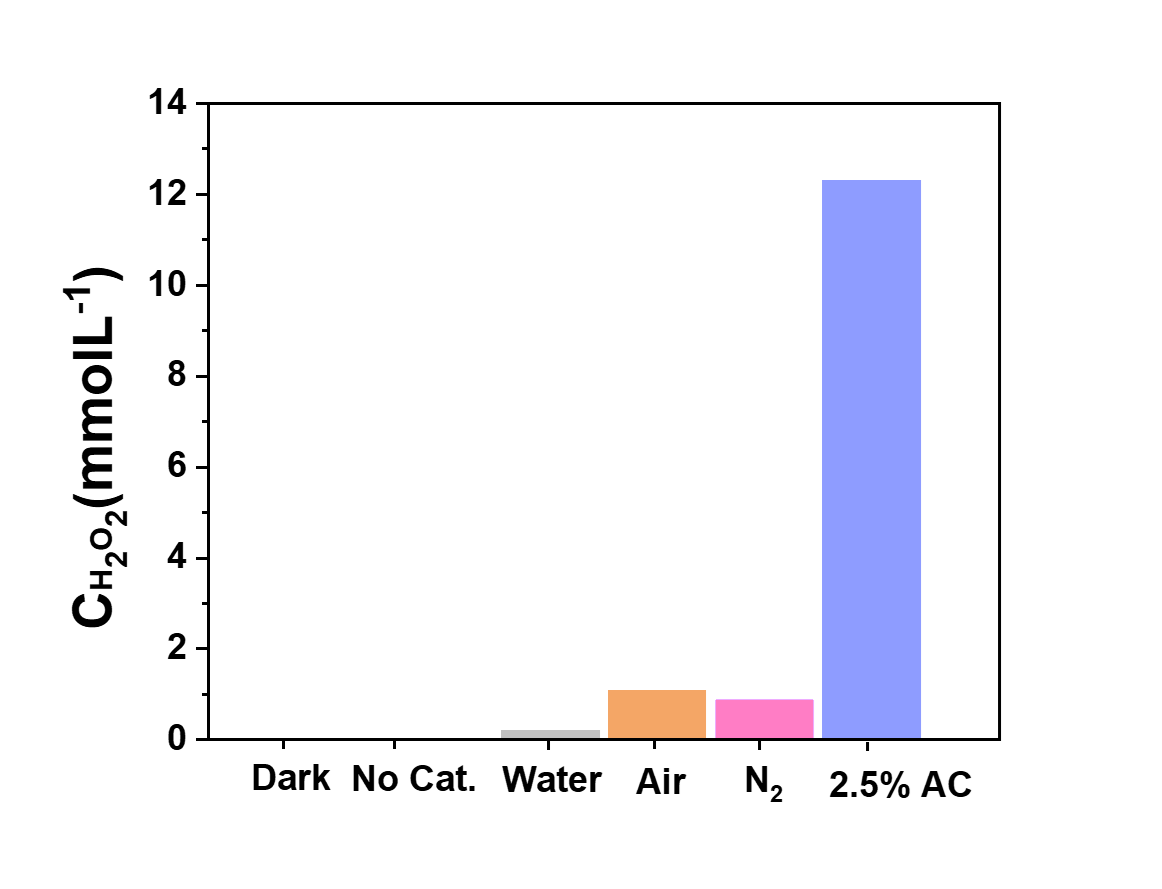


**Figure S10.** Photocatalytic H_2_O_2_ production under different testing conditions.


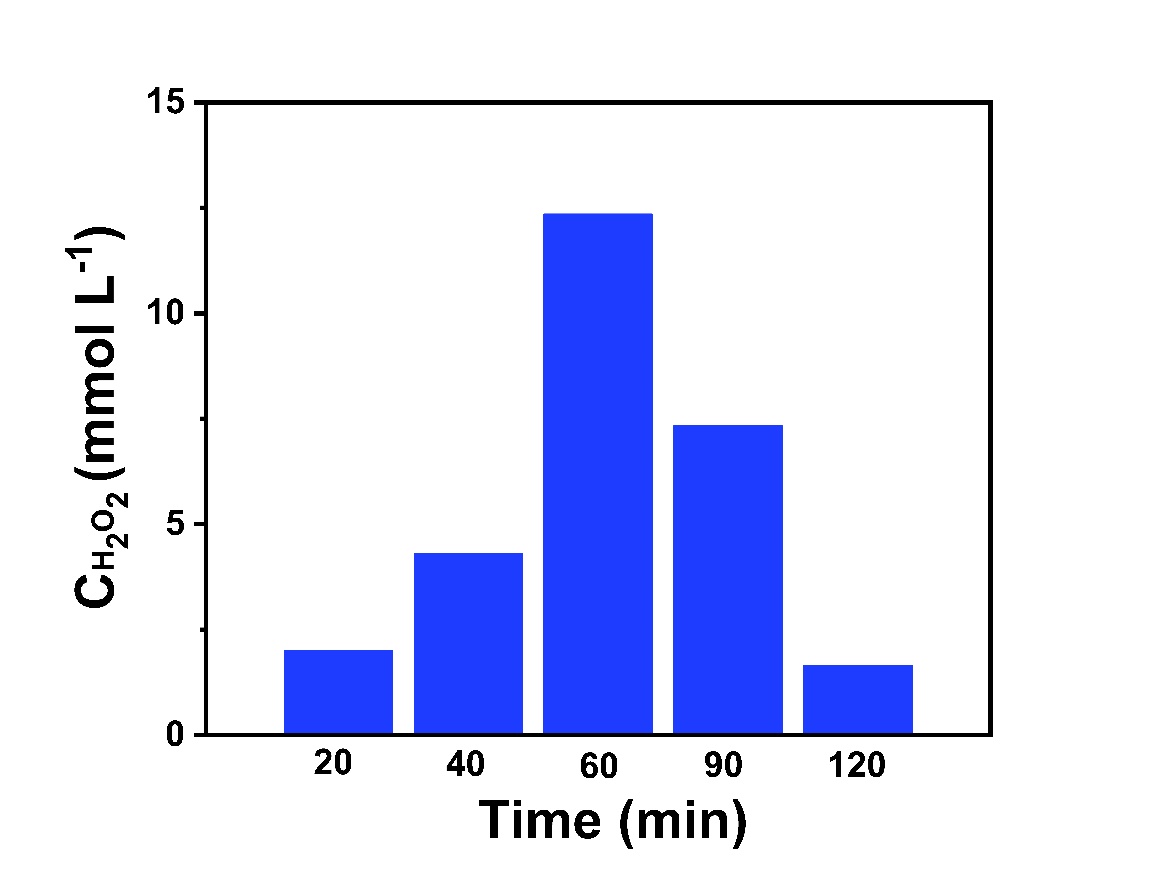


**Figure S11.** Photocatalytic H_2_O_2_ production under different reaction time for *2.5%AC*.


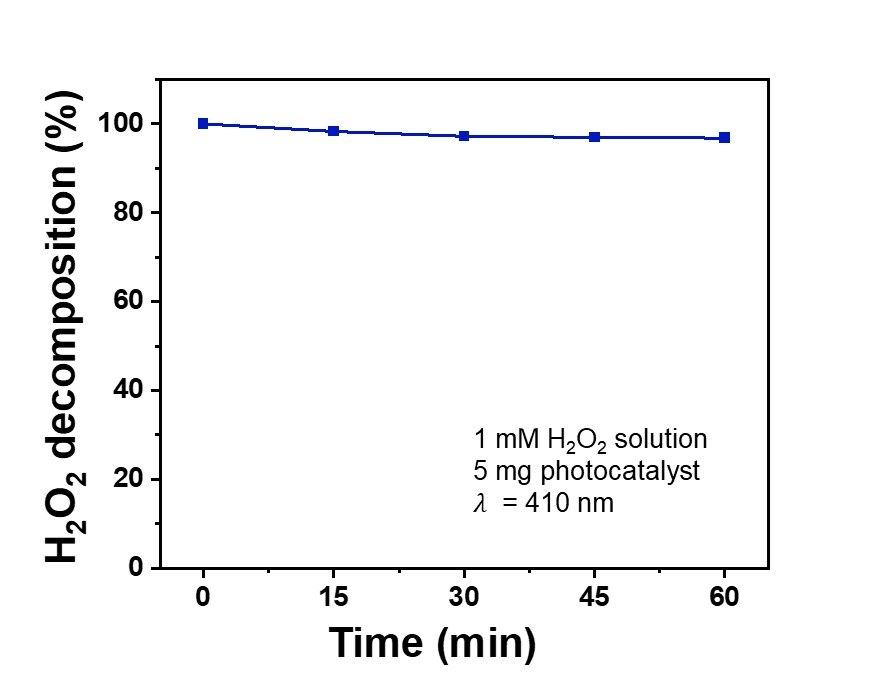


**Figure S12.** Light irradiation of the 5mg 2.5% AC and 1mM H_2_O_2_ solution under different reaction time.

**
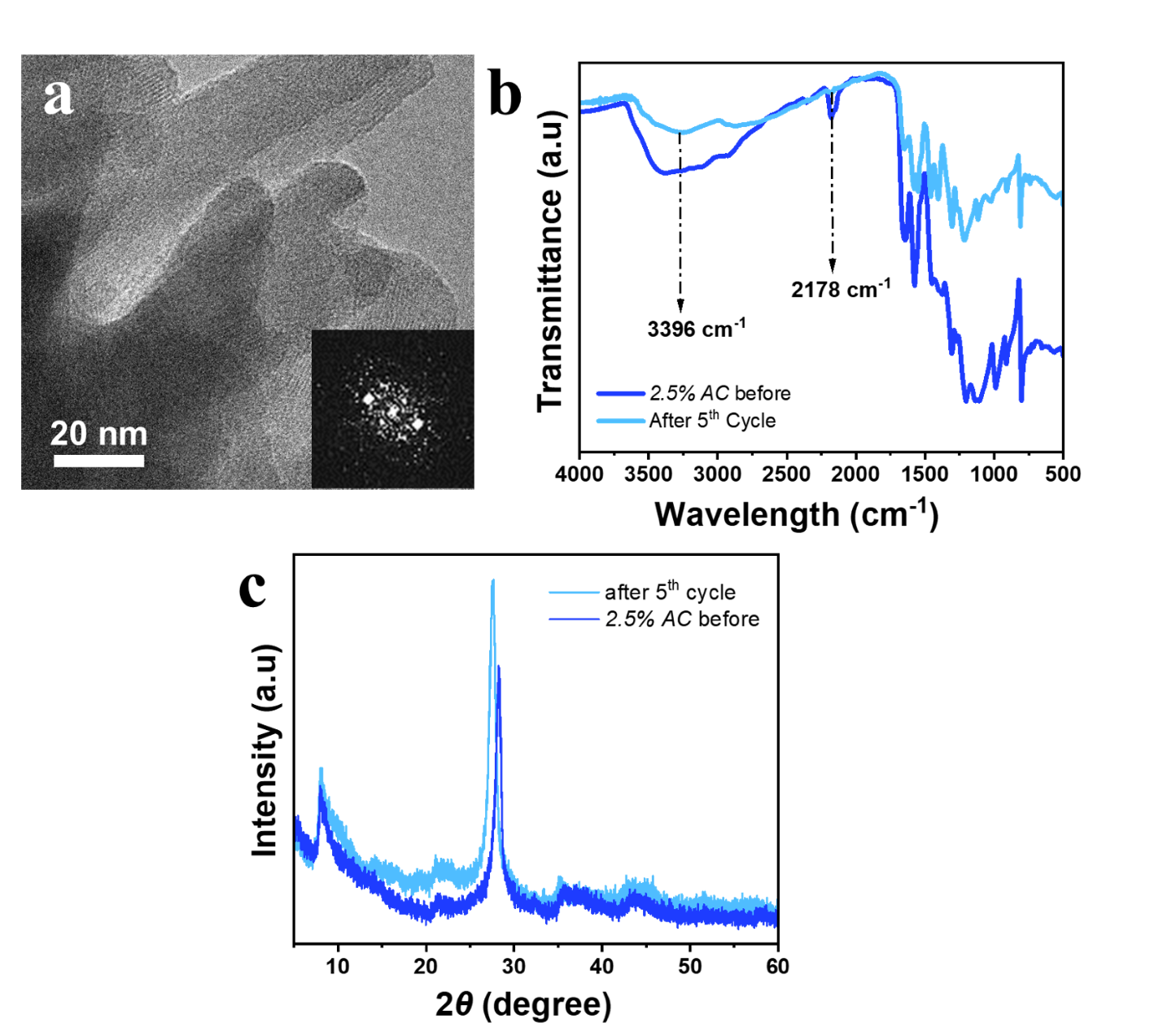
**

**Figure S13.** **(a)** TEM image of used *2.5%AC* catalyst, **(b)** FTIR spectra and **(c)** XRD patterns of *2.5%AC* catalyst before and after H_2_O_2_ production recyclability test.


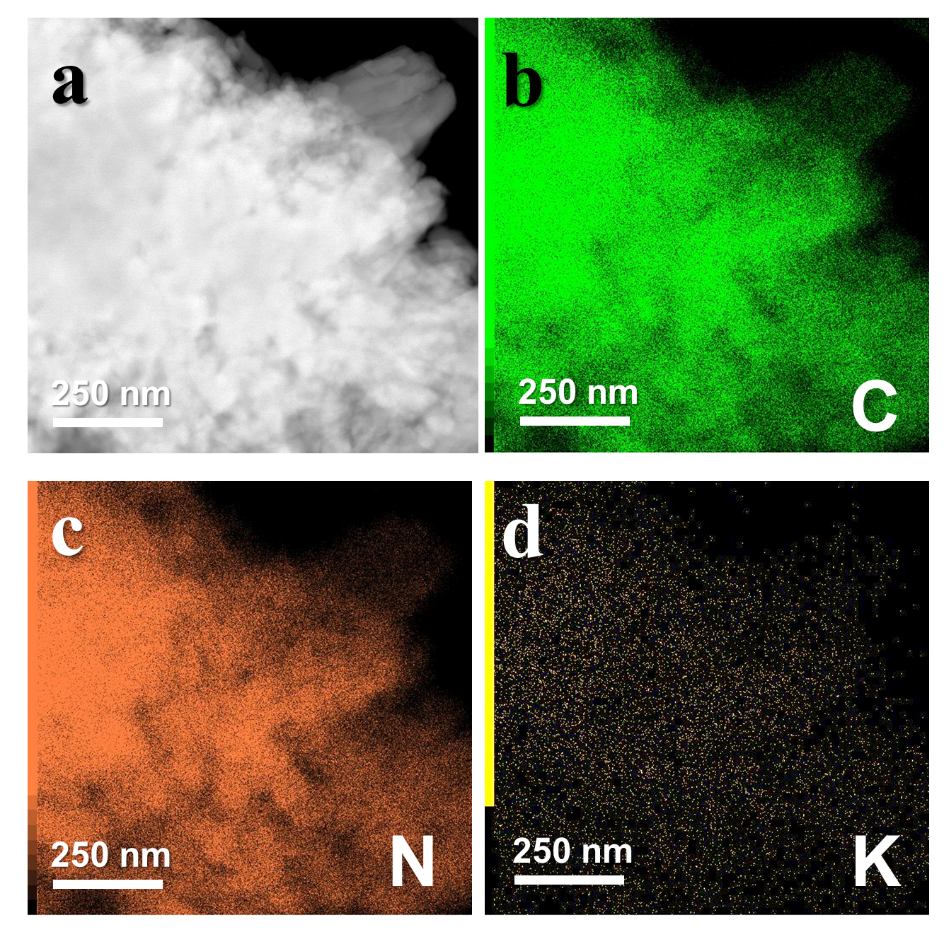


**Figure S14.** EDX mapping of the *2.5%AC* catalyst after H_2_O_2_ production recyclability tests.


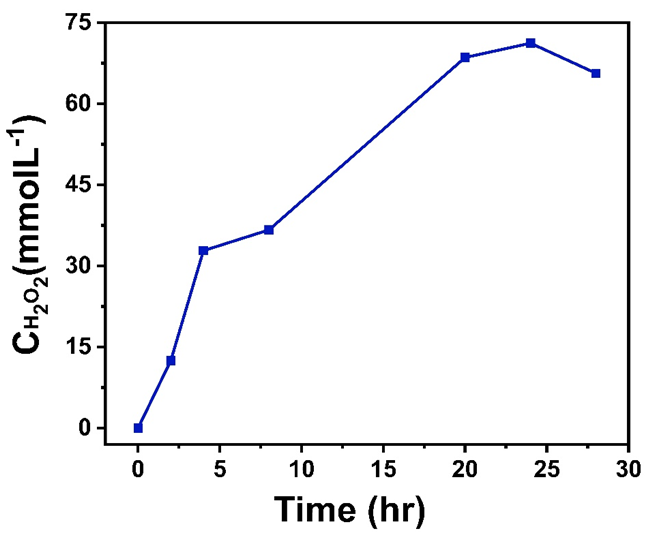


**Figure S15.** Long-term photocatalytic H_2_O_2_production using 2.5% AC in an aqueous glycerol solution under continuous O_2_ flow. Photocatalytic conditions: 100 mL of 10 wt.% aqueous glycerin solution, 100 mg catalyst.


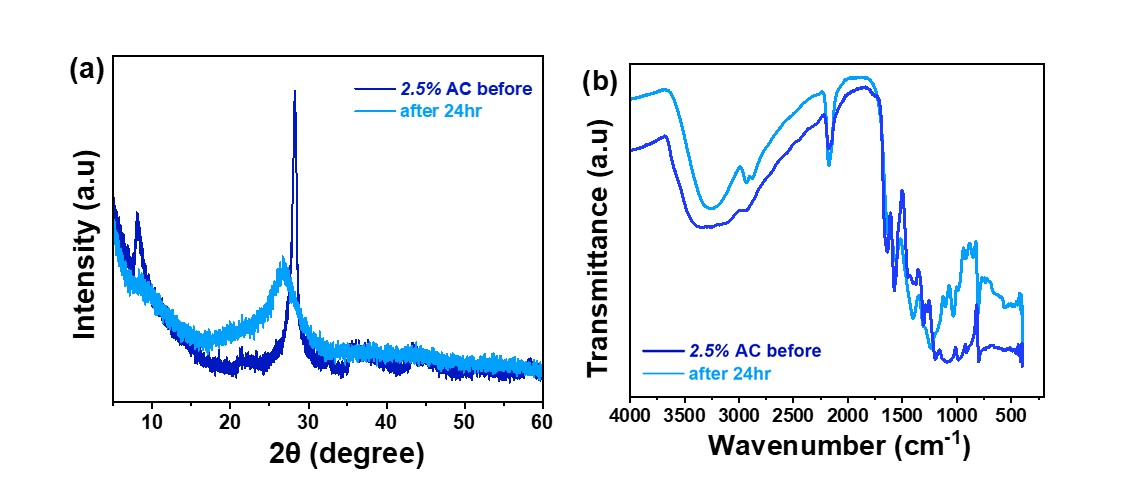


**Figure S16.** **(a)** XRD pattern and **(b)** FTIR spectra of *2.5%AC* catalyst before and after long term (28hr) photocatalytic H_2_O_2_ production.

**Table** **S3.** Comparison of H_2_O_2_ production yields and AQY values with other carbon nitride photocatalysts.

| **Photocatalyst** | **Modification** | **Synthesis Method** | **Conditions** | **H₂O₂ Yields (μmol·g⁻¹·h⁻¹)** | **AQY (%)** | **Ref.** |
| --- | --- | --- | --- | --- | --- | --- |
| Au/g-C_3_N_4_ | Au Co-catalyst | Carbon-layered stabilized | Xe-lamp (λ > 420 nm) | 82.5 | 3.63%  @400nm | [5] |
| Alkali metal-doped Nv-g-C_3_N_4_ | N vacancy, K, Na doping | Thermal copolymerization | 300 W Xe-lamp (λ > 400 nm) | 3,080 | 6.8%  @400nm | [6] |
| Bulk g-C_3_N_4_ | — | Calcination | Xe-lamp (λ > 420 nm) | 125 | 7%  @420nm  0% @500nm | [7] |
| WSe₂/g-C_3_N_4_ | Z-scheme heterojunction | Calcination Solvothermal | Xe-lamp (λ > 420 nm) | 40.62 | 7.18%  @420nm | [8] |
| Ni single-atom/g-C_3_N_4_ | Ni single-atom sites | Not specified | Pure water, 420 nm irradiation | Not specified | 10.9%  @420nm  1.04%  @500nm | [9] |
| g-C_3_N_4_ with hole defects | Hole defects | Photo-assisted heating | AM1.5 (λ > 420 nm) | 5.81 | 10.2%  @420nm | [10] |
| Nv-g-C_3_N_4_ nanosheets | N vacancy | Thermal condensation | Xe-lamp (λ > 420 nm) | 1,768 | 10.5%  @420nm  2.8%  @500nm | [11] |
| Sb-doped g-C_3_N_4_ | Sb doping | Thermal polymerization | LED 420 nm | 3,480 | 17.6%  @420nm | [12] |
| HTCN | H/T junction | Thermal condensation | Visible light (400 < λ < 800 nm) | 22,825.5 | 21.5%  @420nm | [13] |
| Nv–CN–CN | N vacancy, –C≡N group | Two-step calcination | 300 W Xe-lamp (λ ≥ 420 nm) | 3,093 | 22.1%  @420nm | [14] |
| Na-cyanaminate modified g-C_3_N_4_ | Sodium cyanaminate moiety | Molten-salt treatment | Solar simulator, 380 nm | Not specified | 27.6%  @420nm  0.4% @550nm | [15] |
| PDI/CNA | D-A structure | Thermal condensation | LED (400 nm ≤ λ ≤ 760 nm) | 1,605.32 | 27.18%  @400nm  1.48%  @500nm | [16] |
| KPHI | — | Calcination | LED 410 nm | 5,876 | 32.08%  @410nm | [17] |
| Alkali metal-halide modulated PHI | Alkali metal-halide modulation | Polymerization with urea | UV–vis irradiation | Not specified | Not specified | [18] |
| Ox-KPHI | Oxamide induced | Calcination | LED 410 nm | 6,772 | 40.72%  @410nm  0.74%  @520nm | [17] |
| **2.5% AC** | **NH_4_Cl**  **induced** | **Calcination** | **LED 410 nm** | **4,920** | **48.9%**  **@410nm**  **4.5%**  **@525nm** | **This**  **work** |


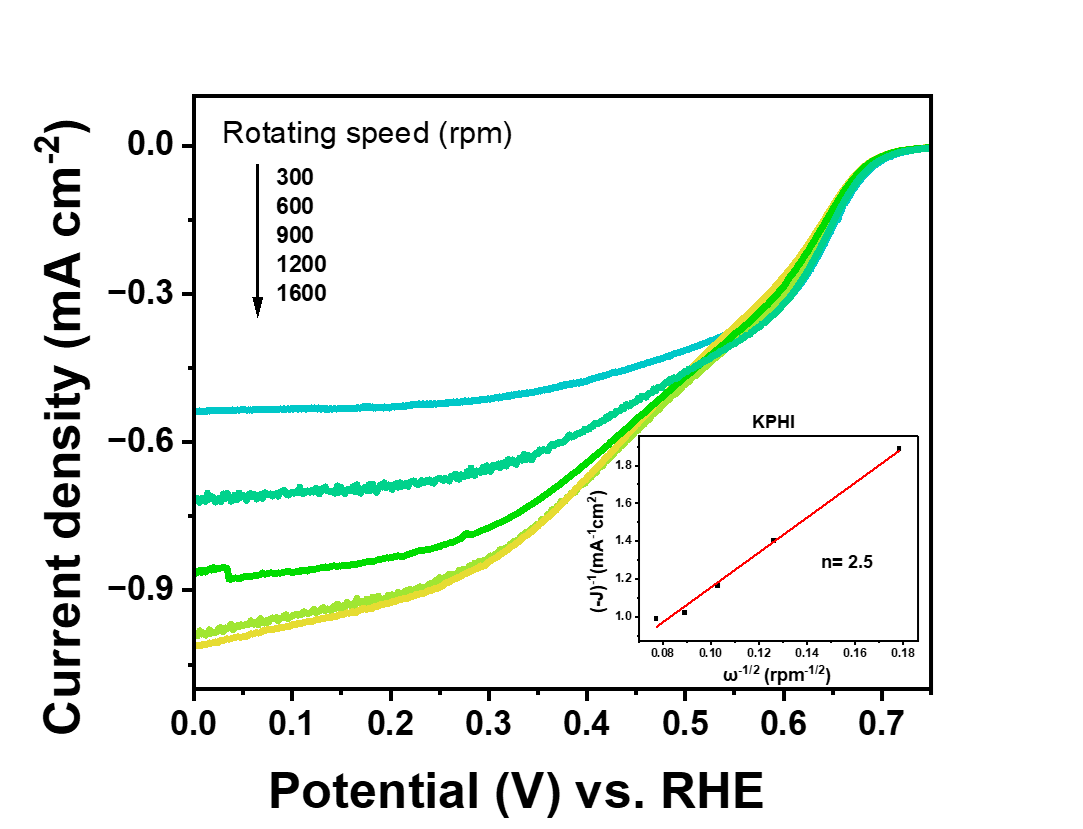


**Figure S17.** Linear sweep voltammetry (LSV) curves of KPHI recorded on a rotating disk glassy carbon electrode with inset graph of K-L.


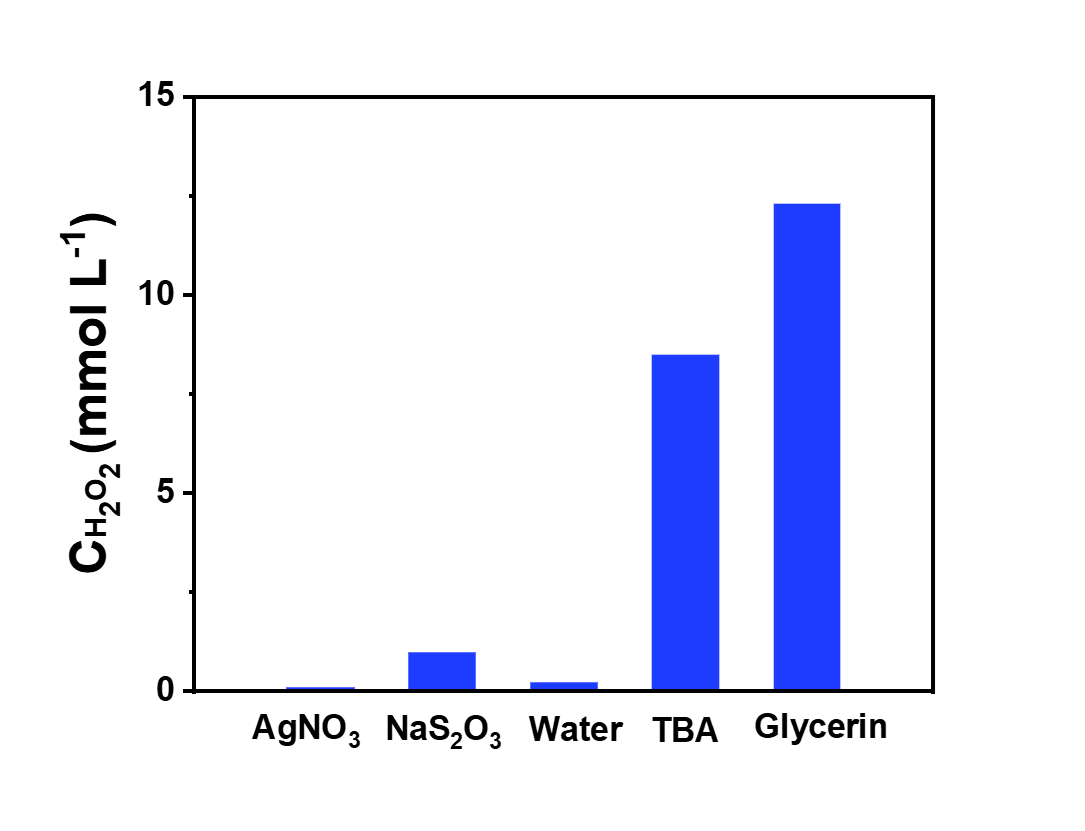


**Figure S18.** Influence of different scavengers on the photocatalytic H_2_O_2_ production of *2.5%AC.*

**
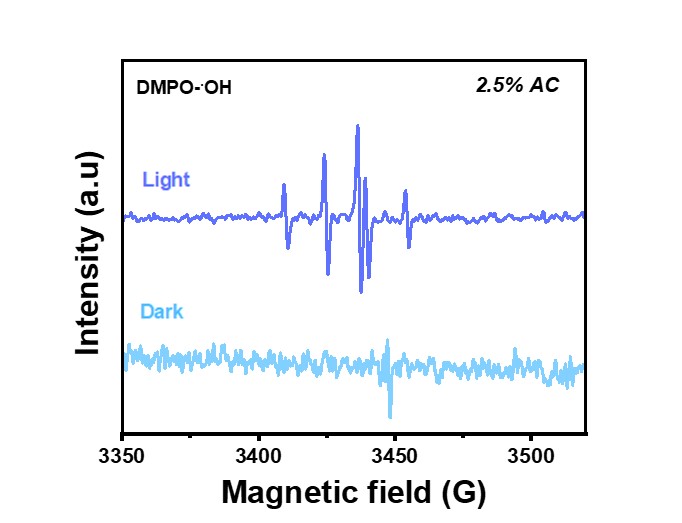
**

**Figure S19.** EPR spectra of DMPO-⋅OH over *2.5%AC*.

**
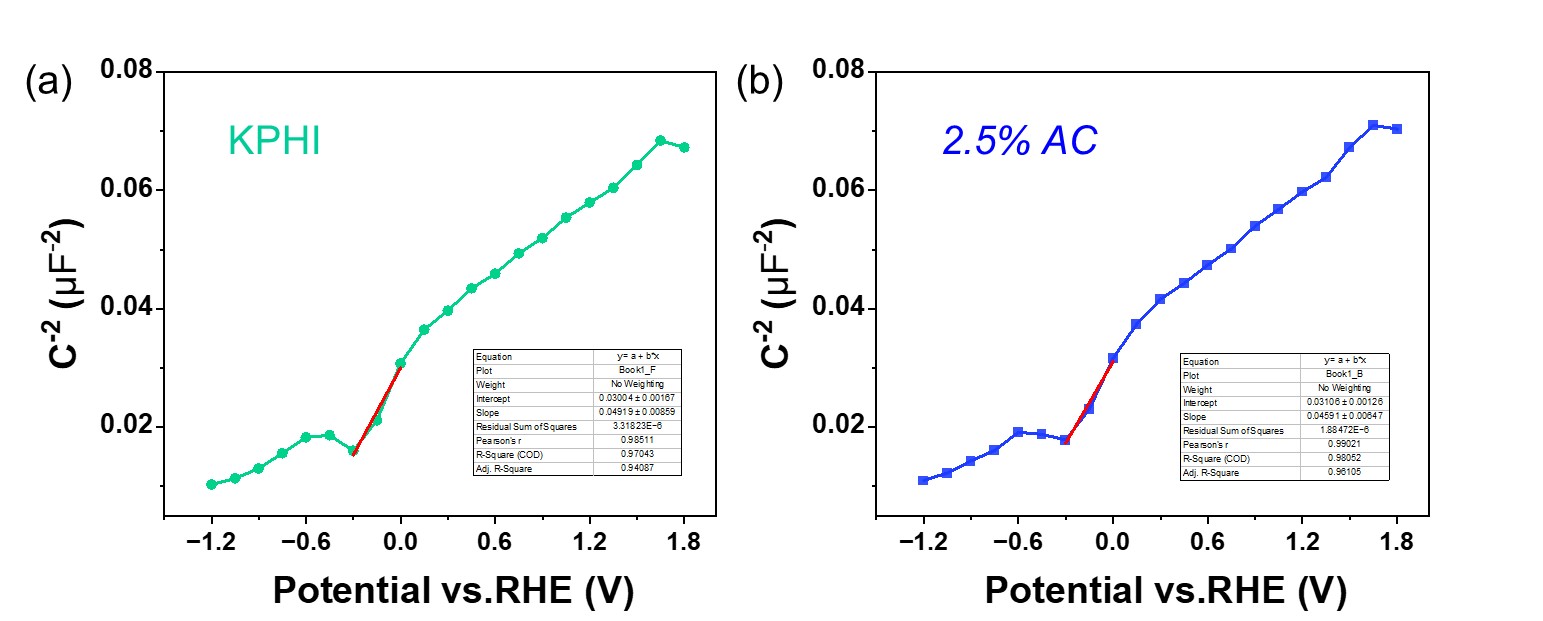
Figure S20.** Mott-Schottky plot of (a) KPHI and (b) *2.5%AC*.

**
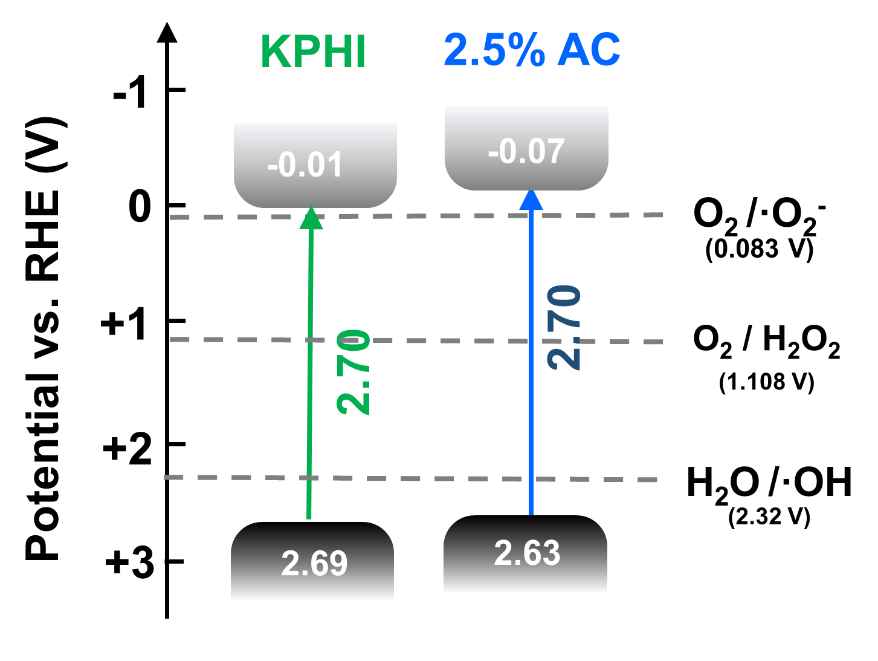
**

**Figure S21.** Experimentally determined band structures of KPHI and *2.5% AC*.


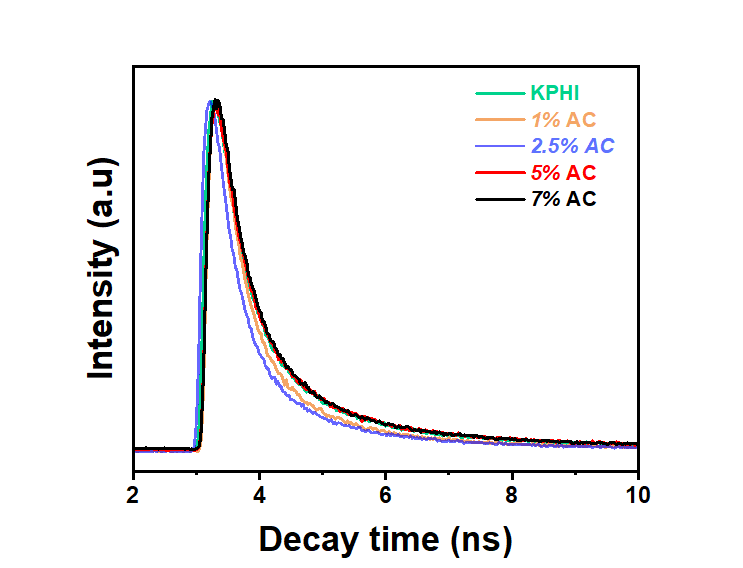


**Figure S22:** Solid-state time-resolved PL decay of KPHI and *X%AC* catalysts.

**Table S4.** PL lifetime values of KPHI and modified catalysts.

| ***Sample*** | $\boldsymbol{A}_{\boldsymbol{1}}$ | $\boldsymbol{\tau}_{\boldsymbol{1}}$**(ns)** | $\boldsymbol{A}_{\boldsymbol{2}}$ | $\boldsymbol{\tau}_{\boldsymbol{2}}$**(ns)** | $\boldsymbol{A}_{\boldsymbol{3}}$ | $\boldsymbol{\tau}_{\boldsymbol{3}}$**(ns)** | $\boldsymbol{\tau}_{\boldsymbol{ave}}$**(ns)** |
| --- | --- | --- | --- | --- | --- | --- | --- |
| **KPHI** | 0.88 | 3.67 | 6.84 | 0.82 | 17.59 | 0.15 | 1.46 |
| **1% AC** | 0.47 | 4.65 | 3.77 | 1.01 | 14.67 | 0.36 | 1.40 |
| **2.5%AC** | 0.74 | 3.8 | 6.73 | 0.72 | 22.4 | 0.12 | 1.38 |
| **5% AC** | 0.43 | 5.31 | 4.61 | 0.43 | 2.72 | 1.36 | 2.26 |
| **7% AC** | 0.39 | 5.09 | 2.76 | 1.36 | 4.58 | 0.43 | 2.08 |


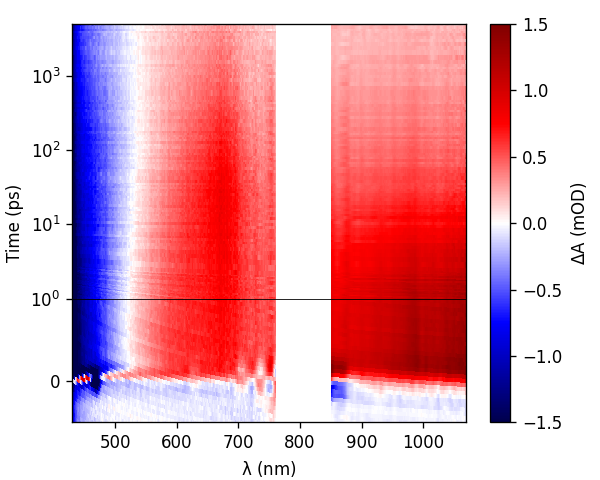

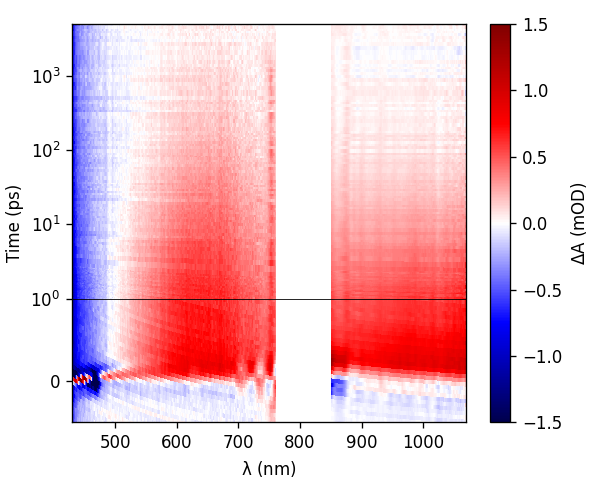


**b**

**a**


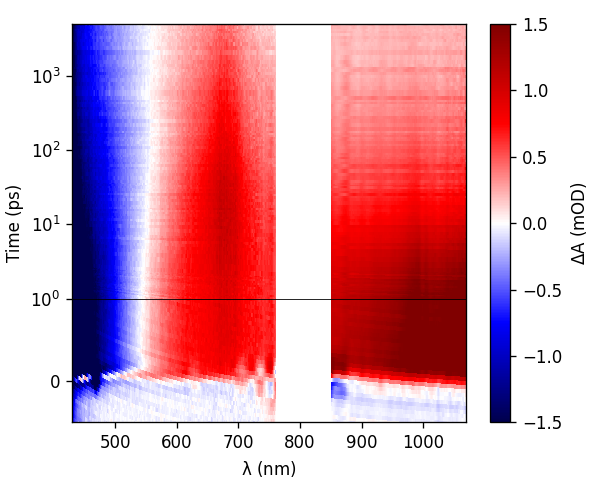

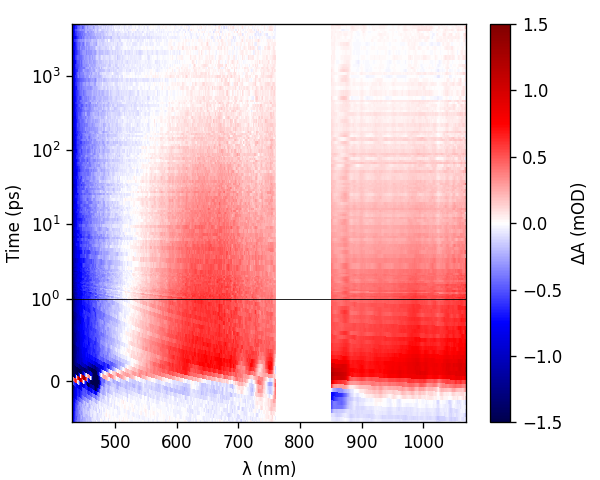


**c**

**d**

**Figure S23.** 2D plots of fs-TAS for **a)** KPHI (600), **b)** KPHI (600) + 10% Gly, **c)** 2.5% AC (600), and **d)** 2.5% AC (600) + 10% Gly. The horizontal axis, vertical axis and colour scale represent the monitoring wavelength, pump-probe time delay and the differential absorbance intensities, respectively. Measurements were made with 400 nm, 0.1 mJ/cm^2^ excitation.


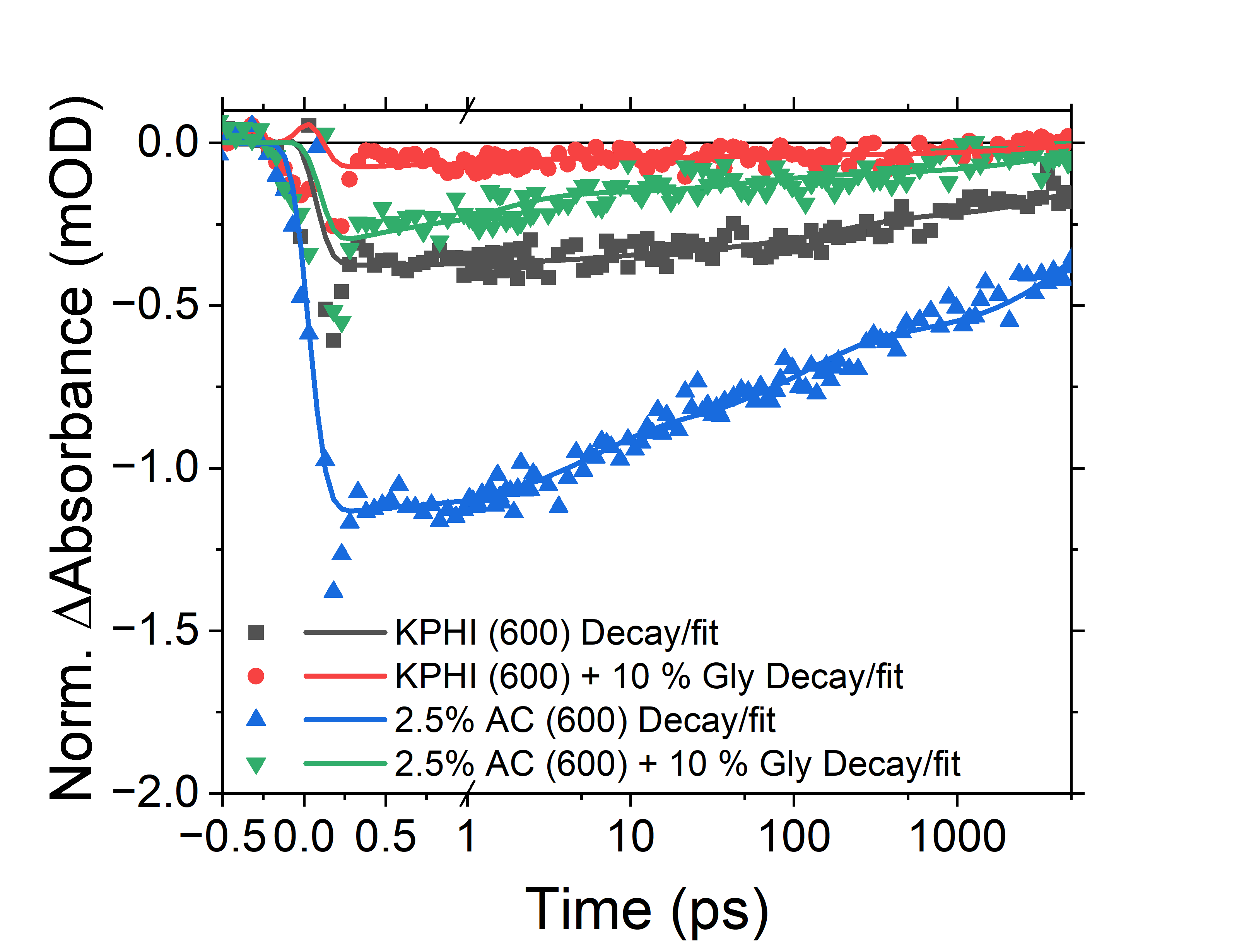

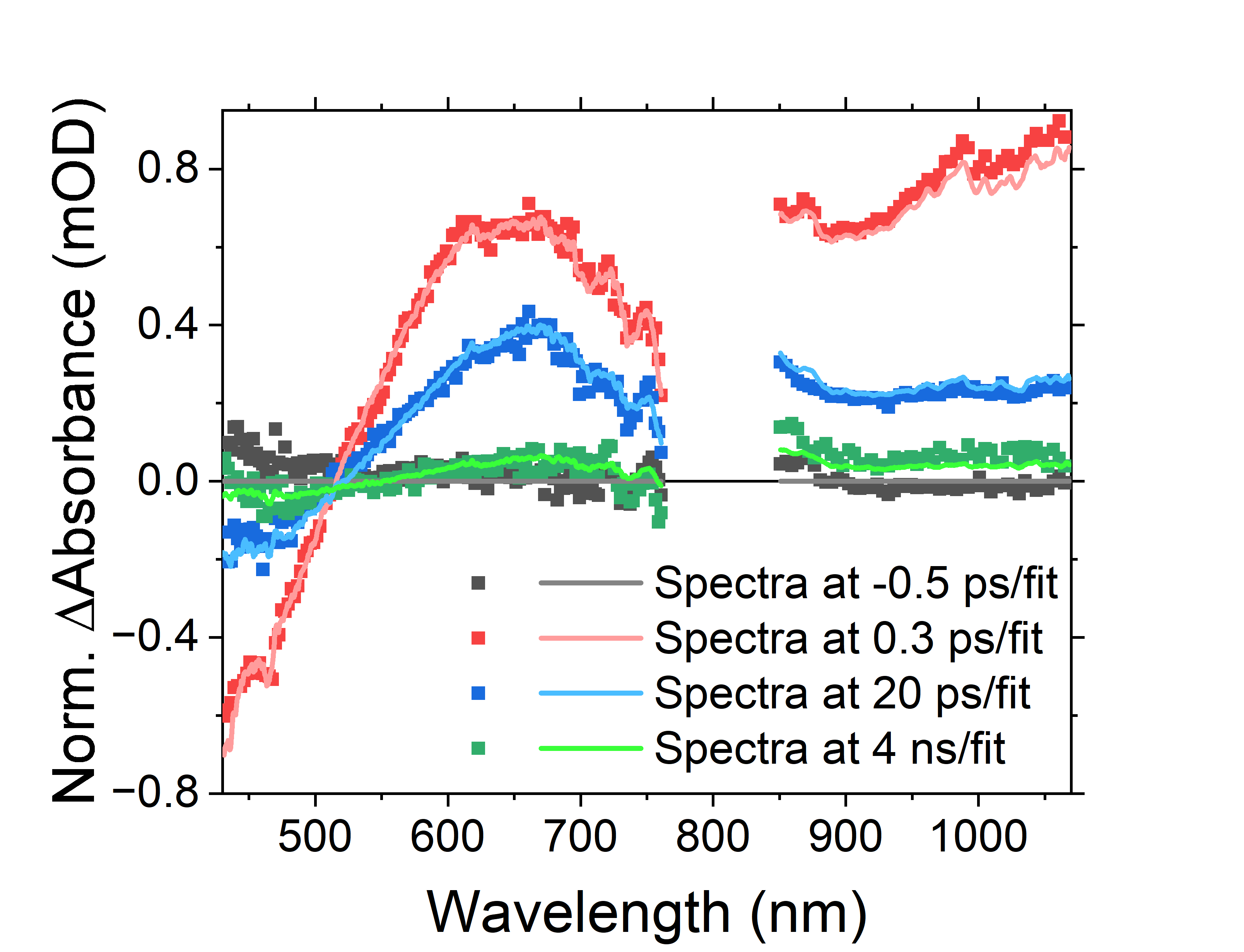


**b**

**a**


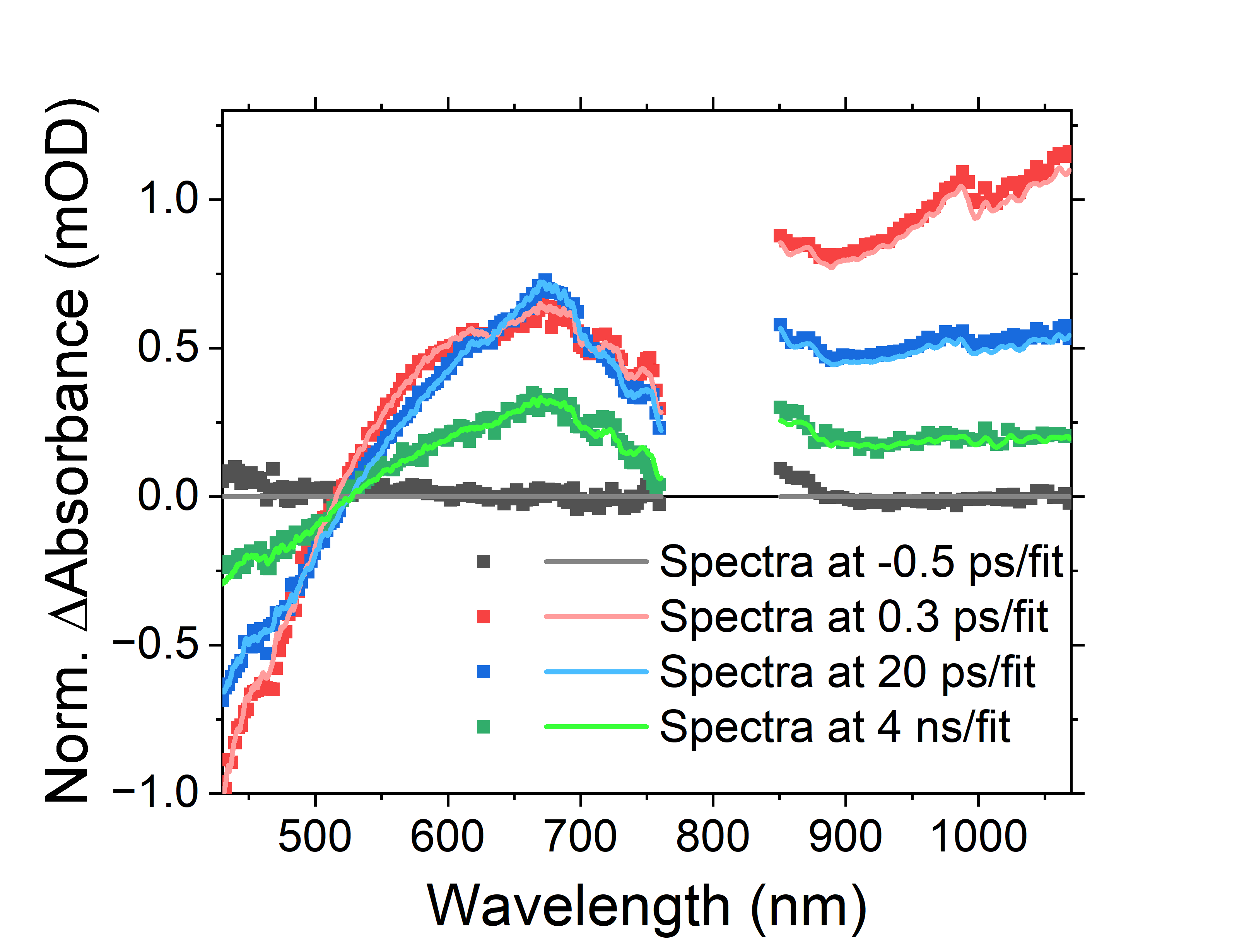

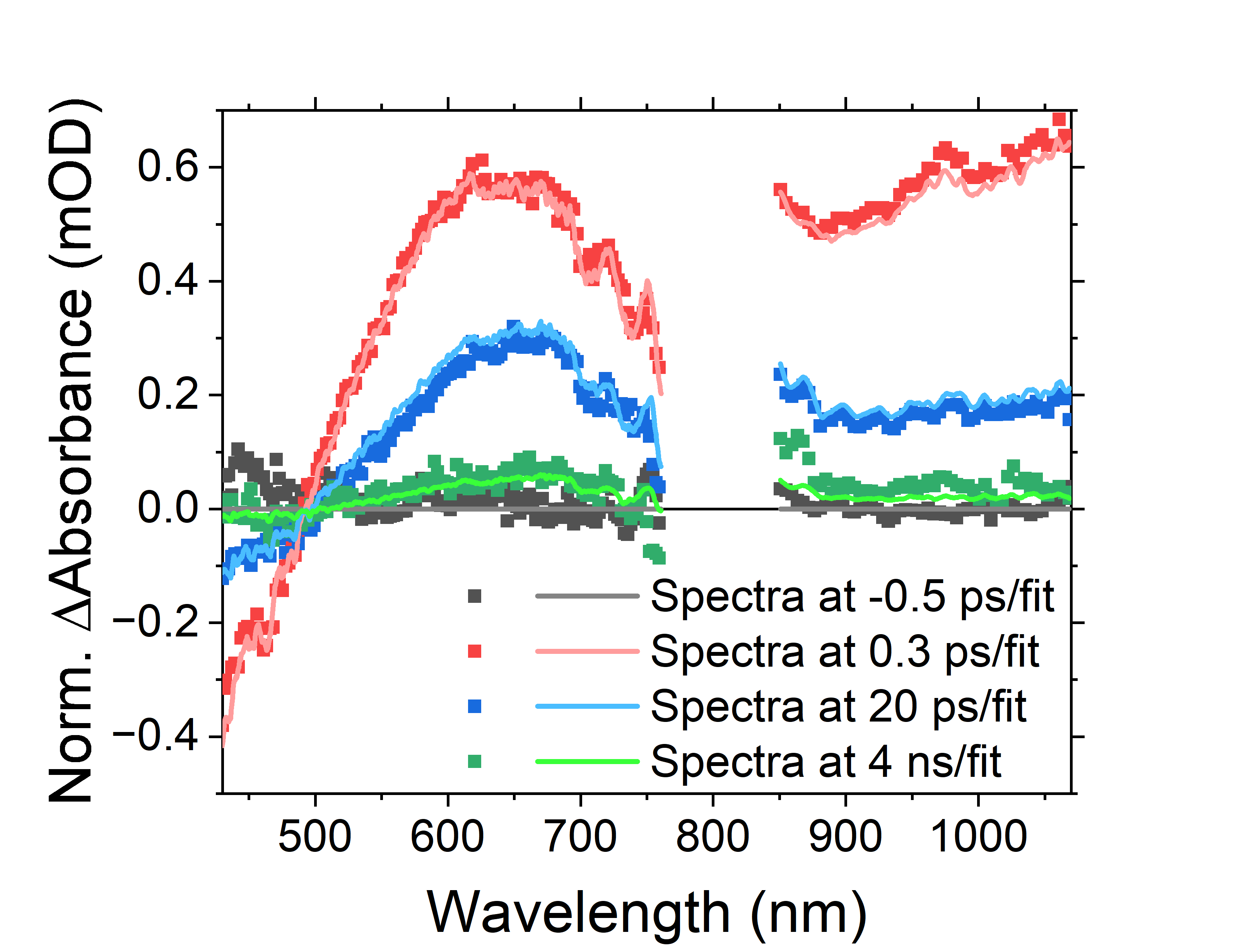


**d**

**c**

**Figure S24.** **a)** The fs-TAS decays normalized to absorbances at 400 nm of 2.5% AC (600) and KPHI (600) monitored at 482 nm. The first picosecond is on a linear scale and the rest is on a logarithmic scale. The fs-TAS spectra at different time delays for **b)** 2.5% AC (600) + 10 % Glycerin, **c)** KPHI (600) and, **d)** KPHI (600) + 10 % Glycerin. The spectra are cut off around 800 nm due to the fundamental laser pulse. Measurements were made with 400 nm, 0.1 mJ/cm^2^ excitation.

**
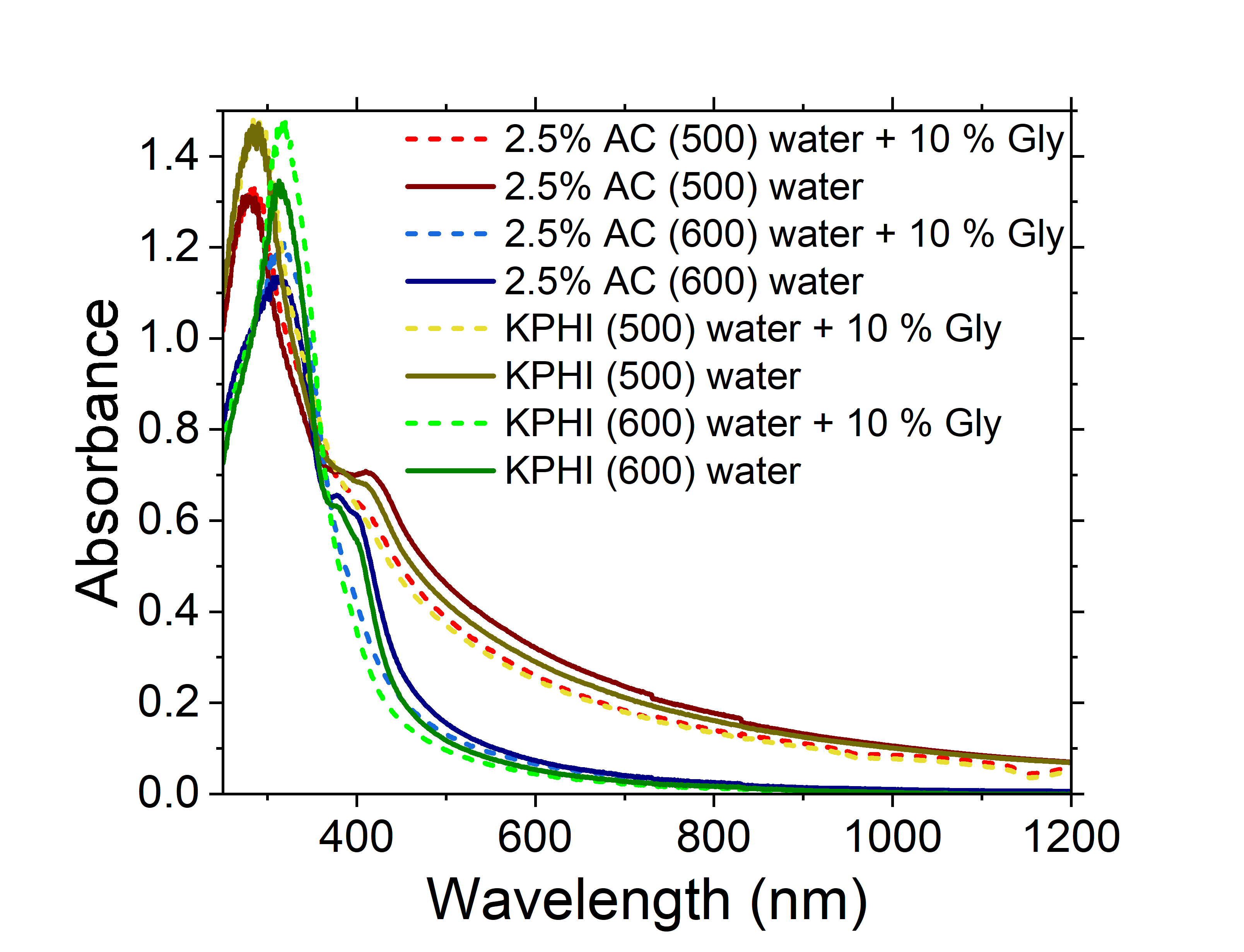
**

**Figure S25.** Absorption spectra of solutions used in fs-TAS measurements.

**Table** **S5.** The time constants (τ) from the global multi-exponential fitting from the fs-TAS measurements for the samples in solutions. Samples marked with (*) used an additional exponent to account for scattering which are not included in the table.

|  | **τ_1_, ps** | **τ_2_, ps** | | **τ_3_, ns** |
| --- | --- | --- | --- | --- |
| KPHI (600)* | 6.4 | | 169 | >5 |
| 2.5% AC (600) | 5.3 | | 113 | >5 |
| KPHI (600) + 10 % Glycerin* | 1.2 | | 36 | 3.46 |
| 2.5% AC (600) + 10 % Glycerin* | 1.2 | | 57 | 4.49 |
| KPHI (500)* | 2.2 | | 107 | >5 |
| 2.5%AC (500)* | 3.5 | | 133 | >5 |
| KPHI (500) + 10 % Glycerin* | 4.0 | | 132 | >5 |
| 2.5%AC (500) + 10 % Glycerin* | 1.1 | | 53 | >5 |


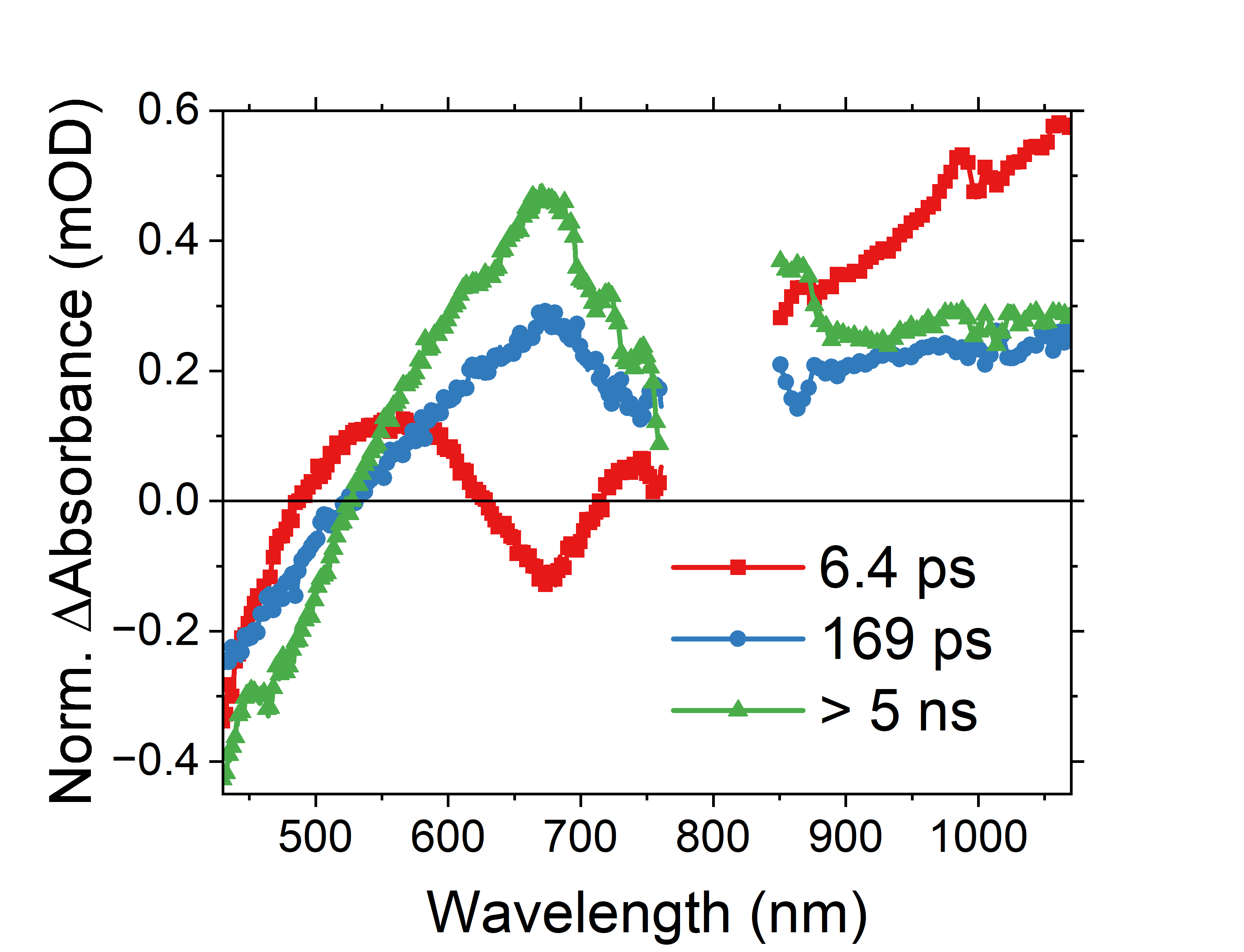

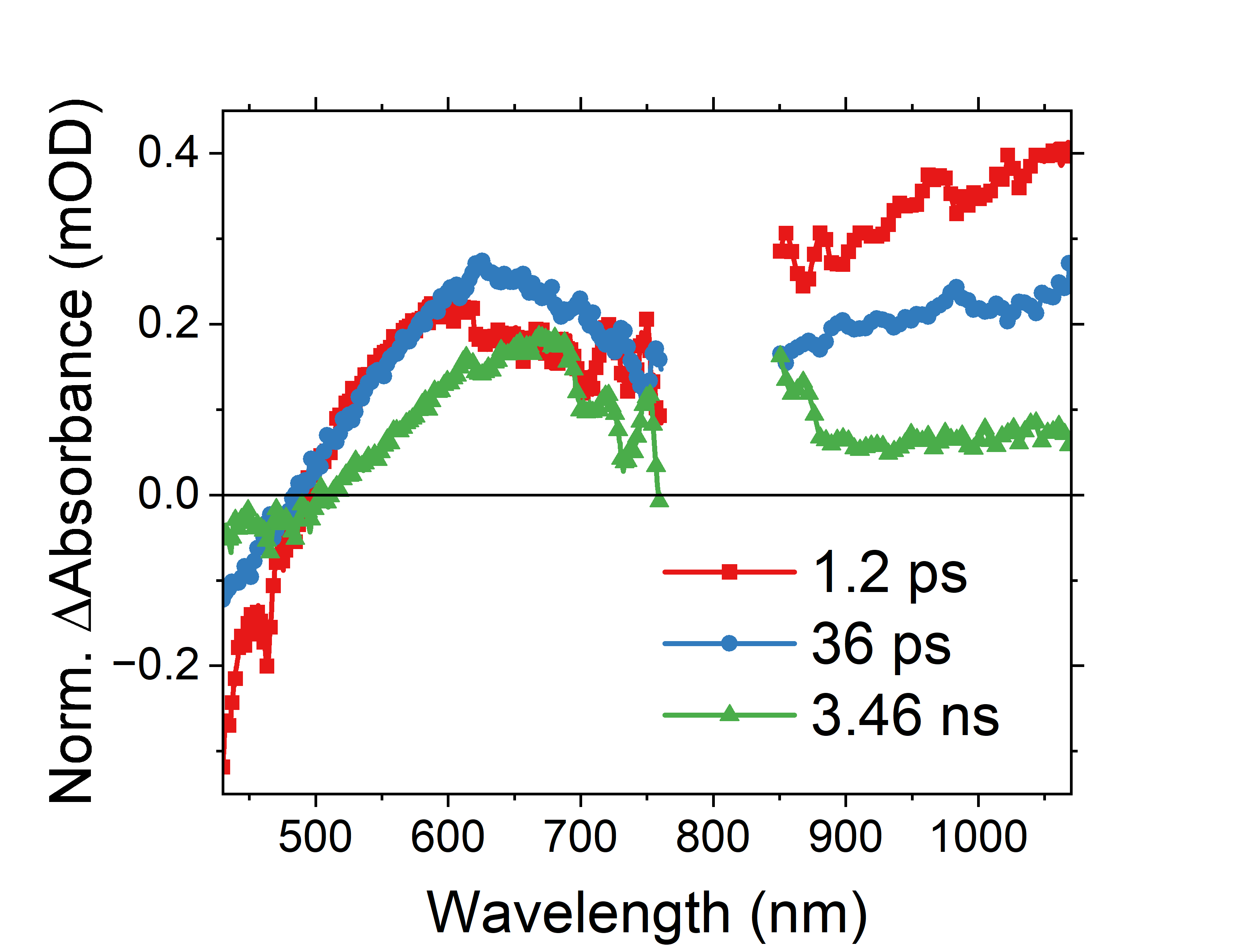

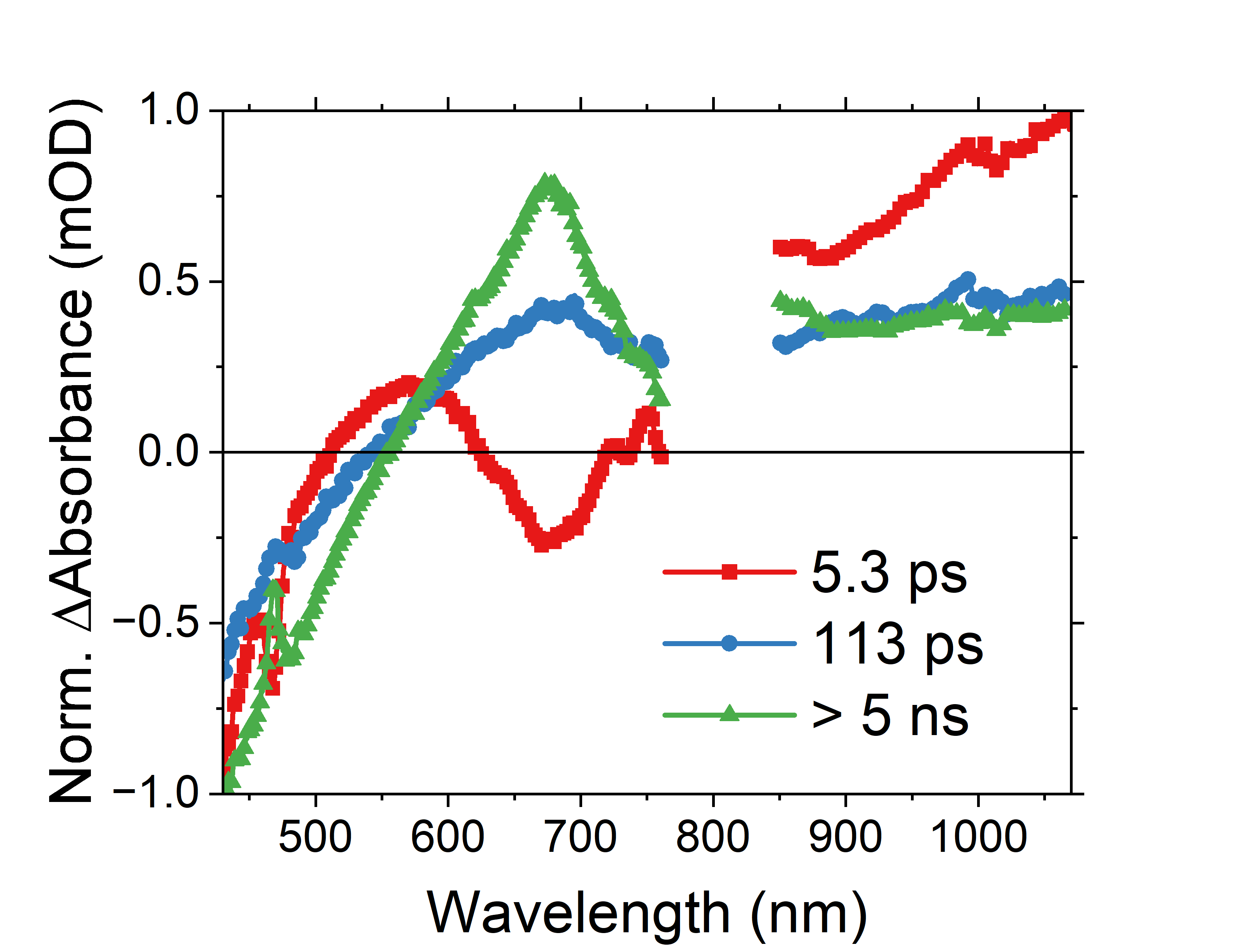

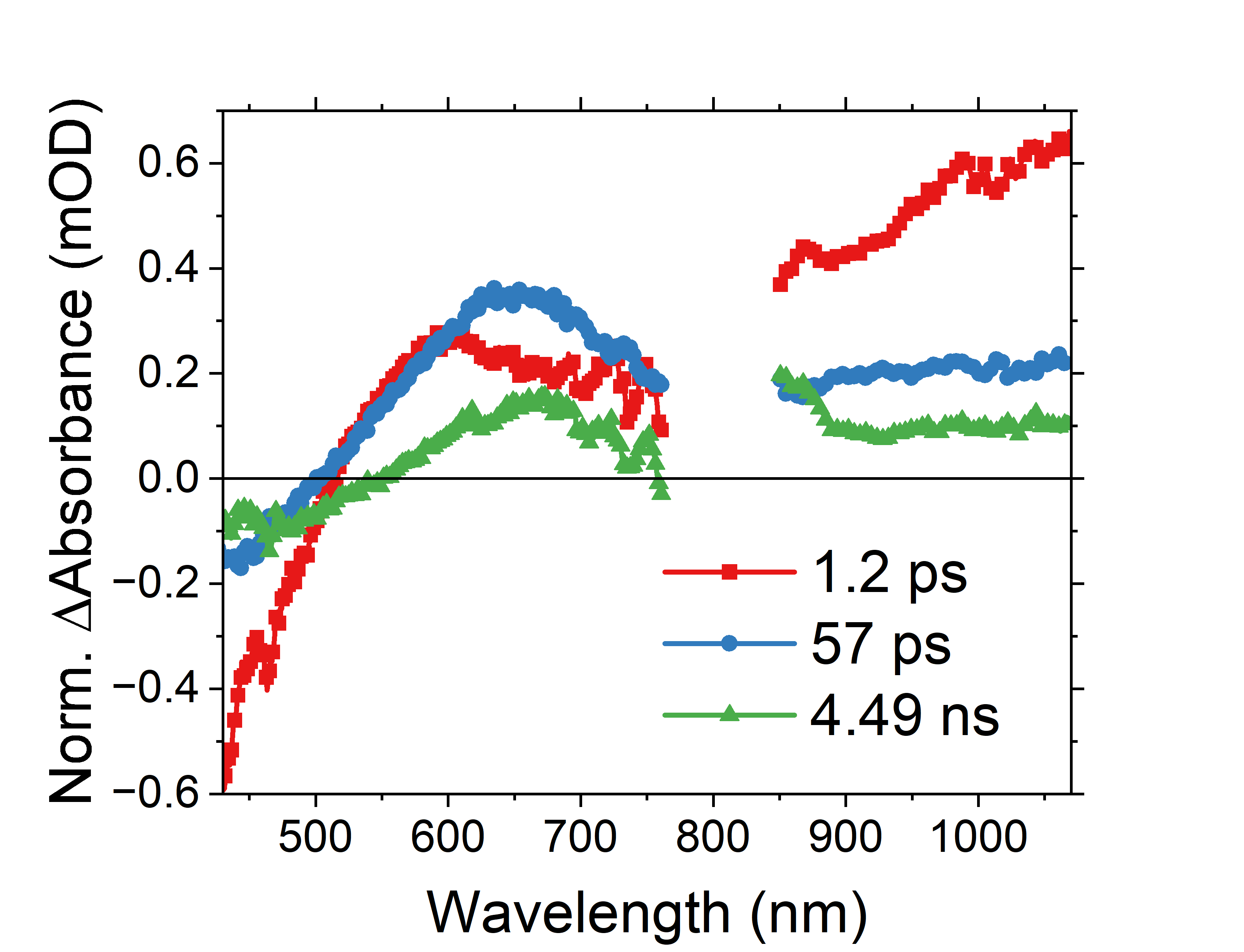


**d**

**c**

**b**

**a**

**Figure S26.** The decay associated spectra of **a)** KPHI (600), **b)** KPHI (600) + 10 % Glycerin, **c)** 2.5% AC (600) and, **d)** 2.5% AC (600) + 10 % Glycerin derived from the multiexponential fitting.

**
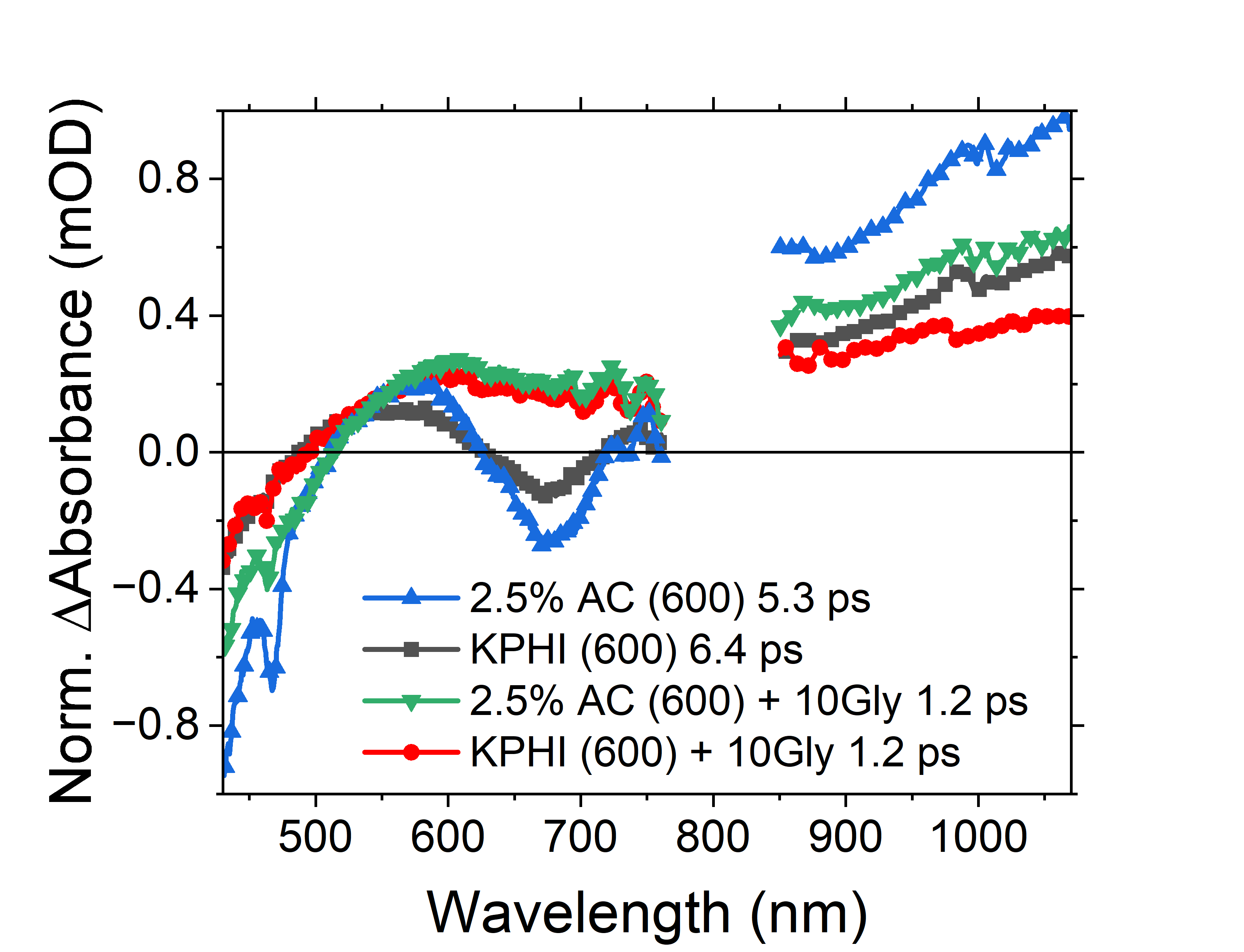
** **
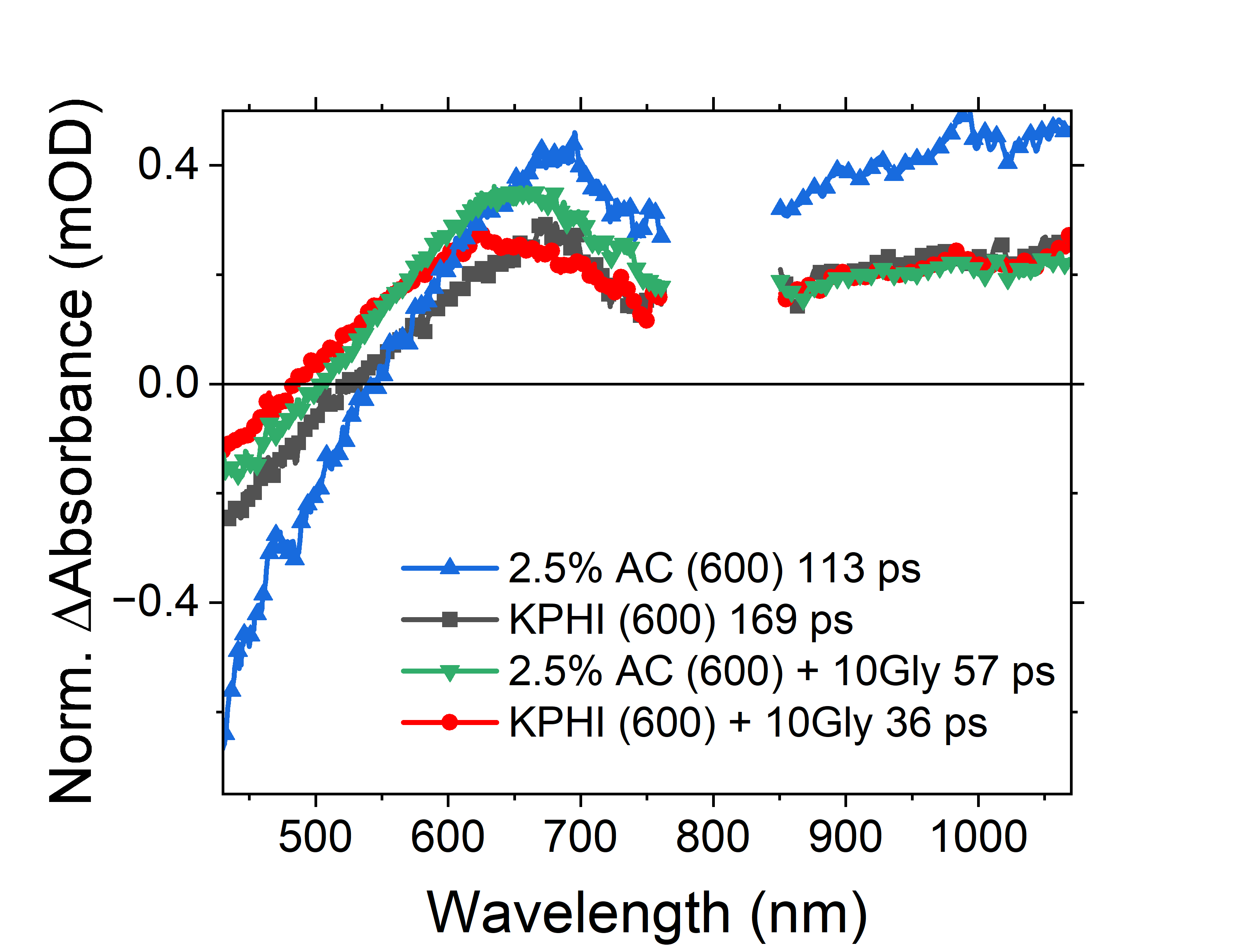

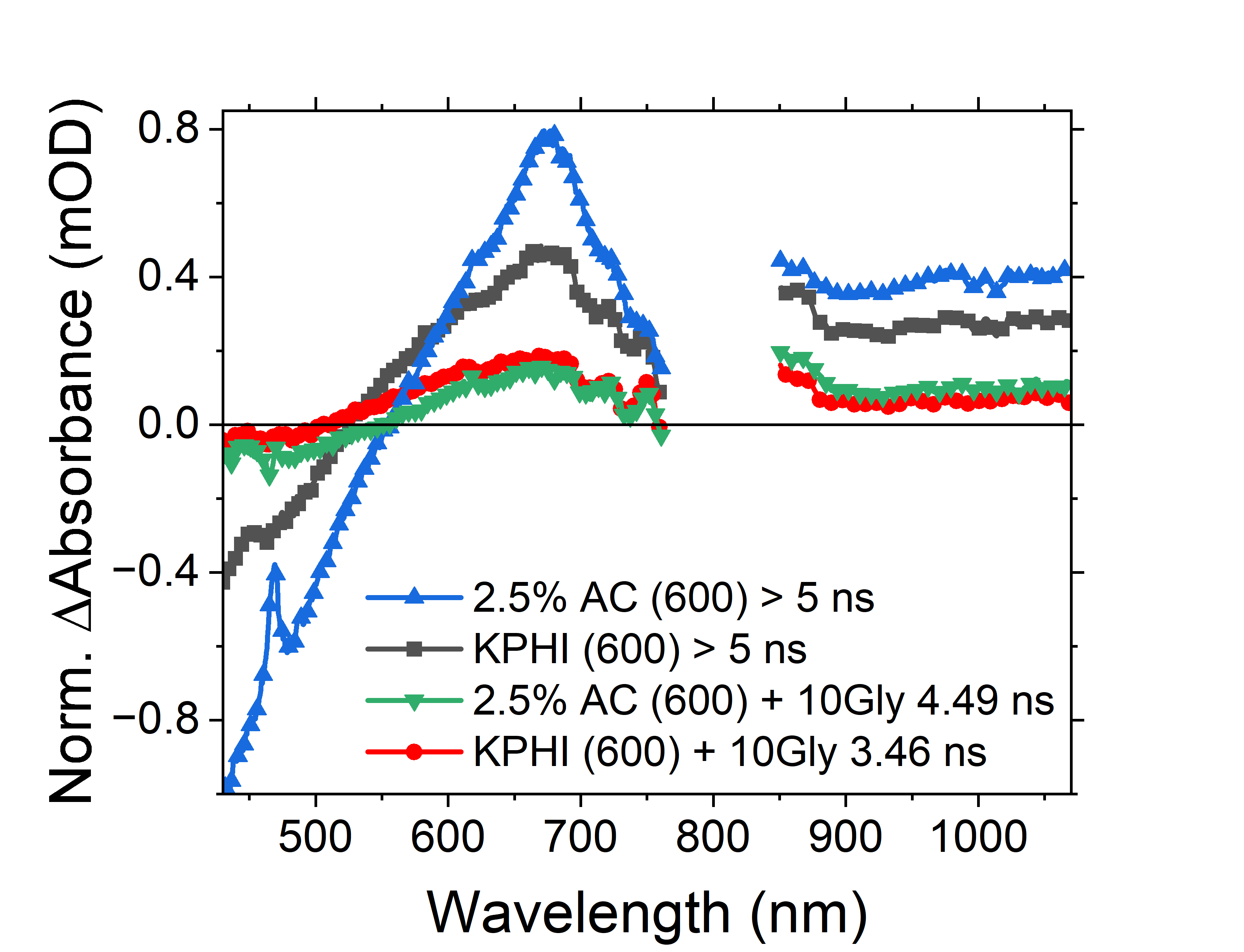
**

**a**

**c**

**b**

**Figure S27.** The decay associated spectra of 2.5% AC (600) and KPHI (600) of the **a)** first **b)** second and, **c)** third non-scattering exponents derived from the multiexponential fitting.


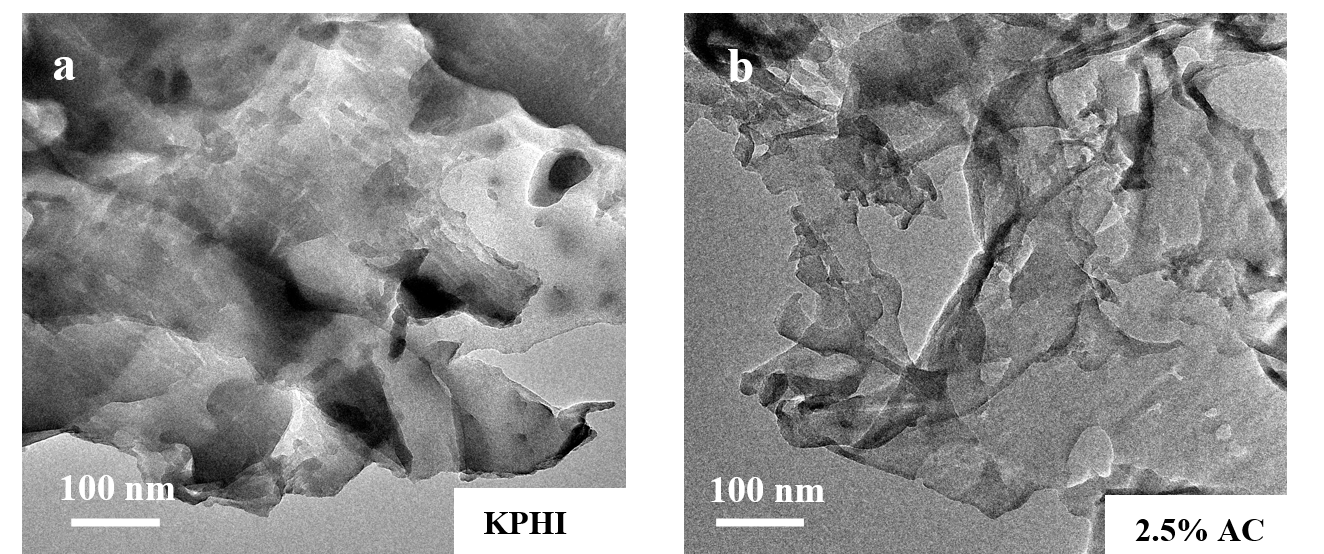


**Figure S28.** TEM images of **a)** KPHI and **b)** *2.5%AC* synthesized at 400°C.


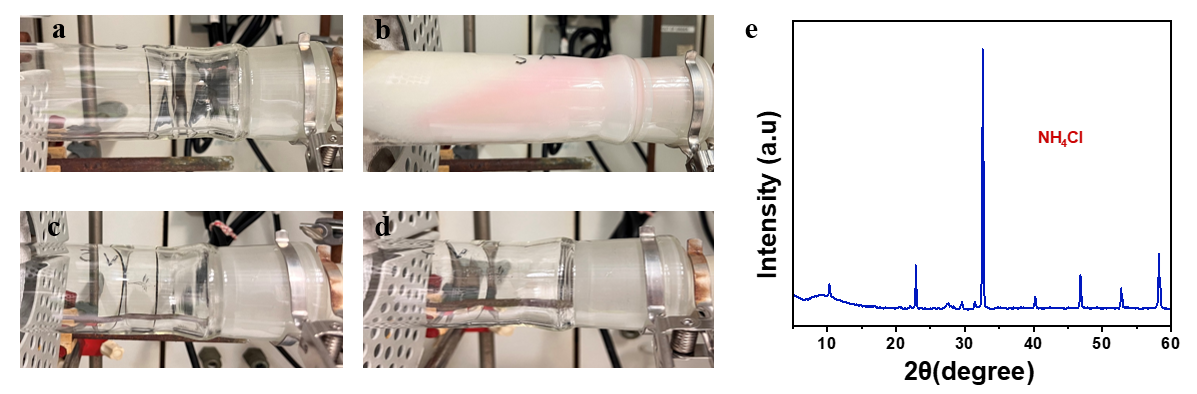

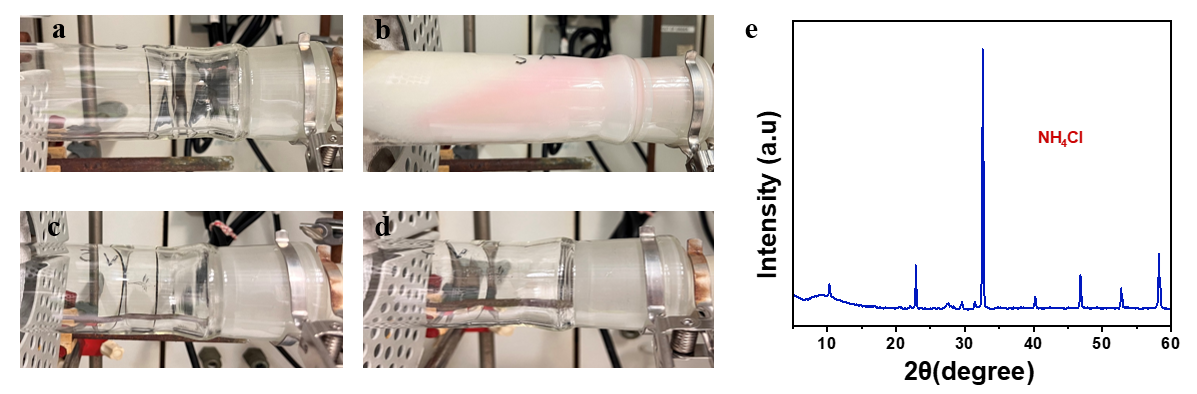


**Figure S29.** **(a)** *2.5*% *AC*@500°C before experiment **(b)** *2.5*% *AC*@500°C after experiment **c)** *KPHI*@500°C before experiment **(d)** *KPHI* @500°C after experiment **(e)** XRD plot of the NH_4_Cl found in the furnace after the synthesis of *2.5*% *AC*@500°C.


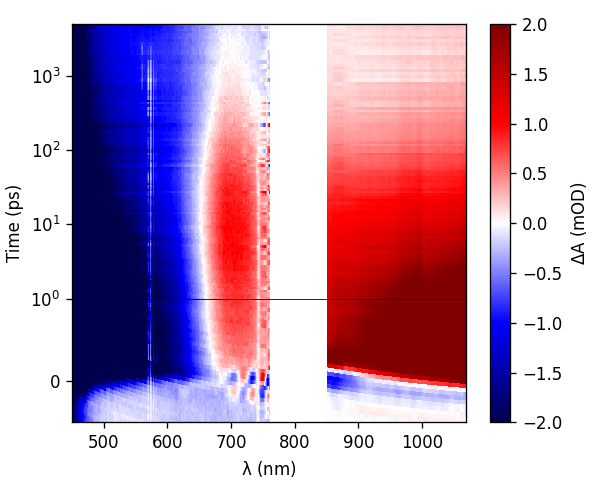

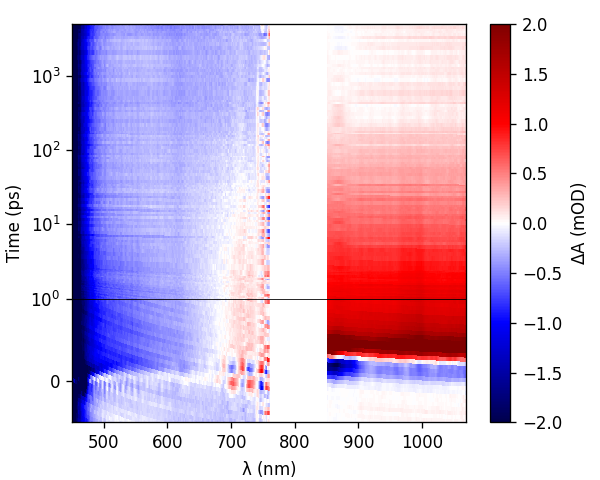


**b**

**a**


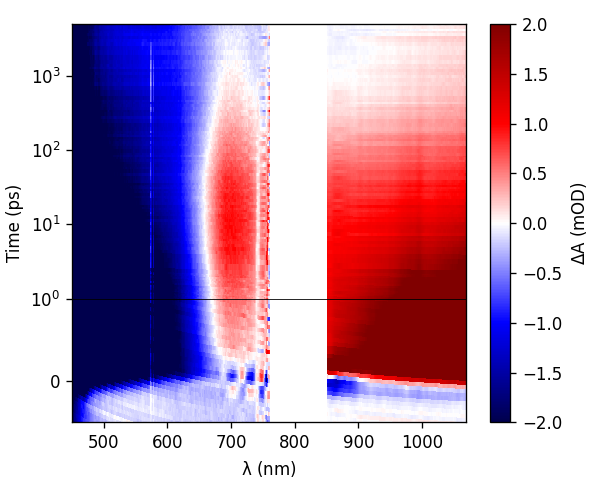

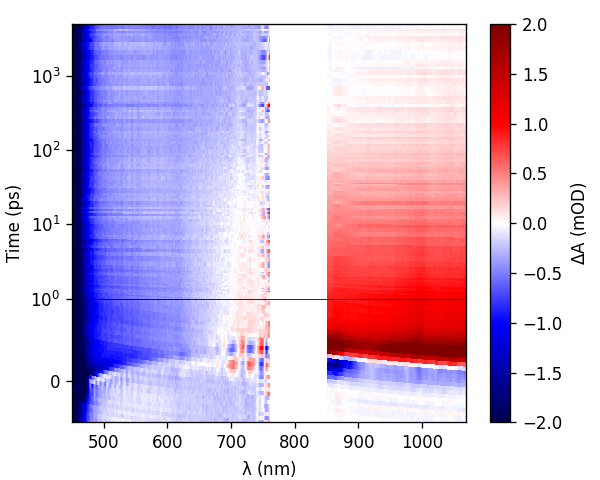


**d**

**c**

**Figure S30.** 2D plots of fs-TAS for **a)** KPHI (500), **b)** KPHI (500) + 10% Gly, **c)** 2.5% AC (500), and **d)** 2.5% AC (500) + 10% Gly. The horizontal axis, vertical axis and colour scale represent the monitoring wavelength, pump-probe time delay and the differential absorbance intensities, respectively. Measurements were made with 400 nm, 0.04 mJ/cm^2^ excitation.

**Table** **S6.** The poly dispersity indexes (PDI) and the zeta-averaged size (nm) of the samples from the dynamic light scattering measurements.

|  | **Size, nm** | **PDI** |
| --- | --- | --- |
| KPHI (600) | 161.8 | 0.18 |
| 2.5%AC (600) | 168.9 | 0.13 |
| KPHI (500) | 442 | 0.30 |
| 2.5%AC (500) | 437.9 | 0.26 |
|  |  |  |


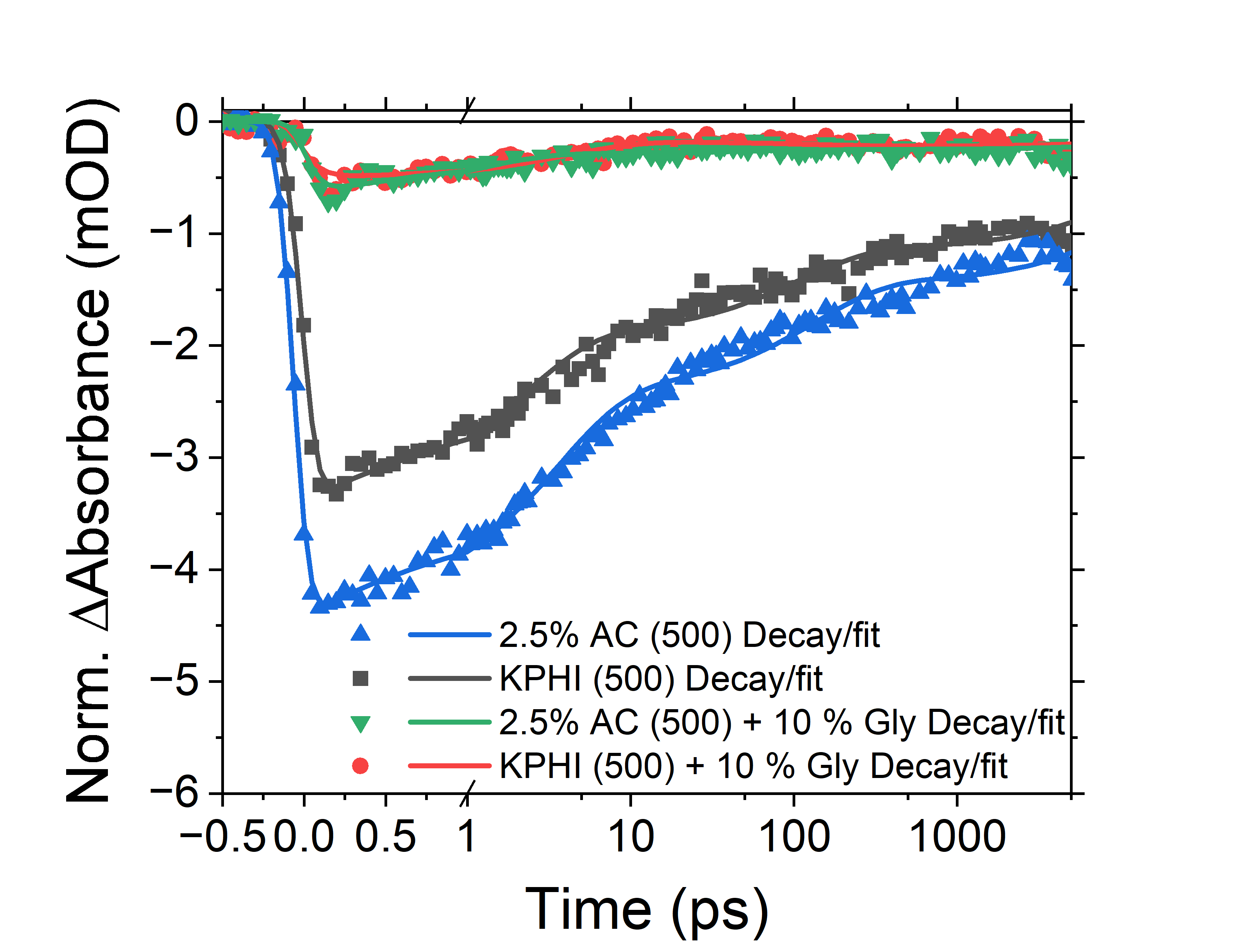

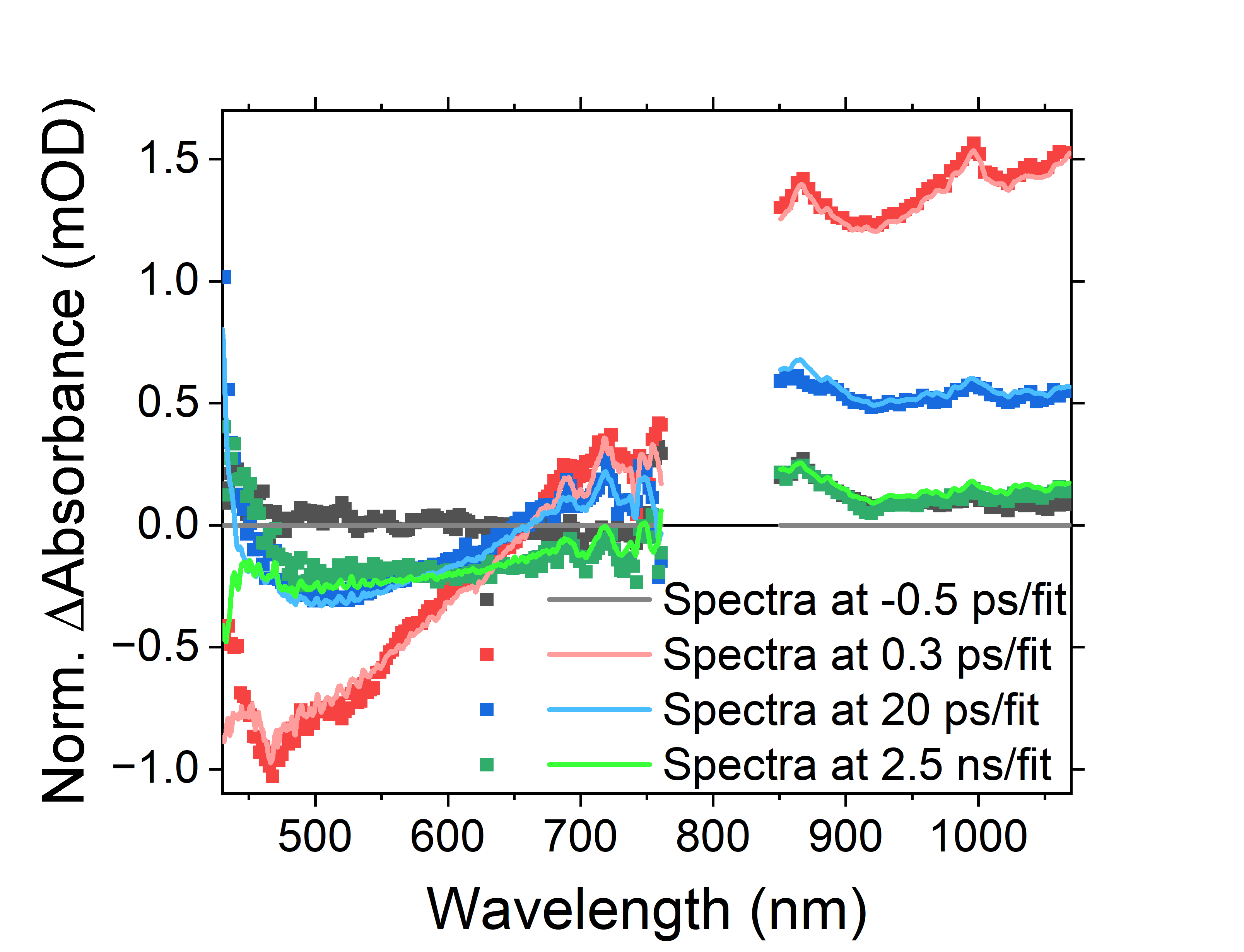


**a**

**b**


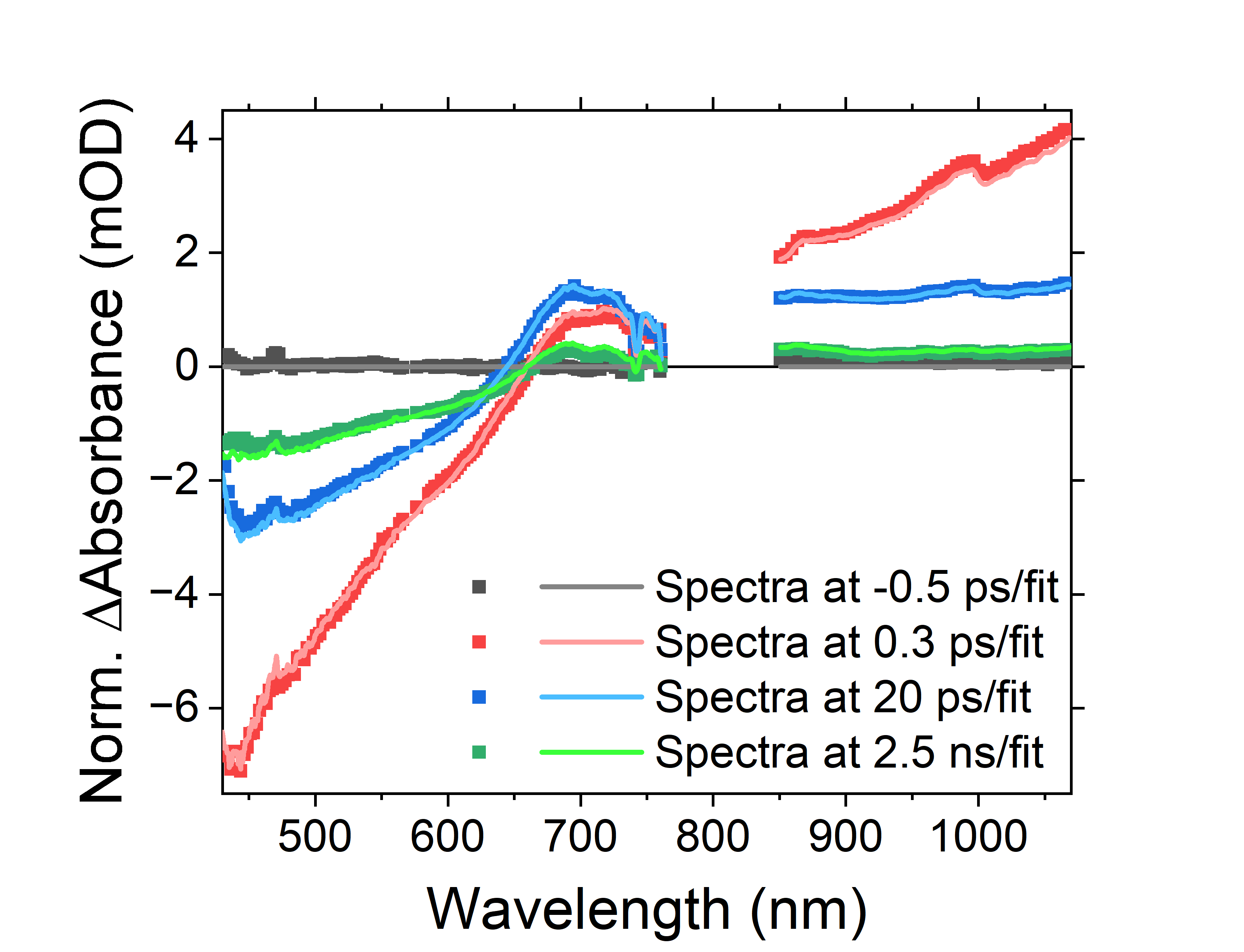

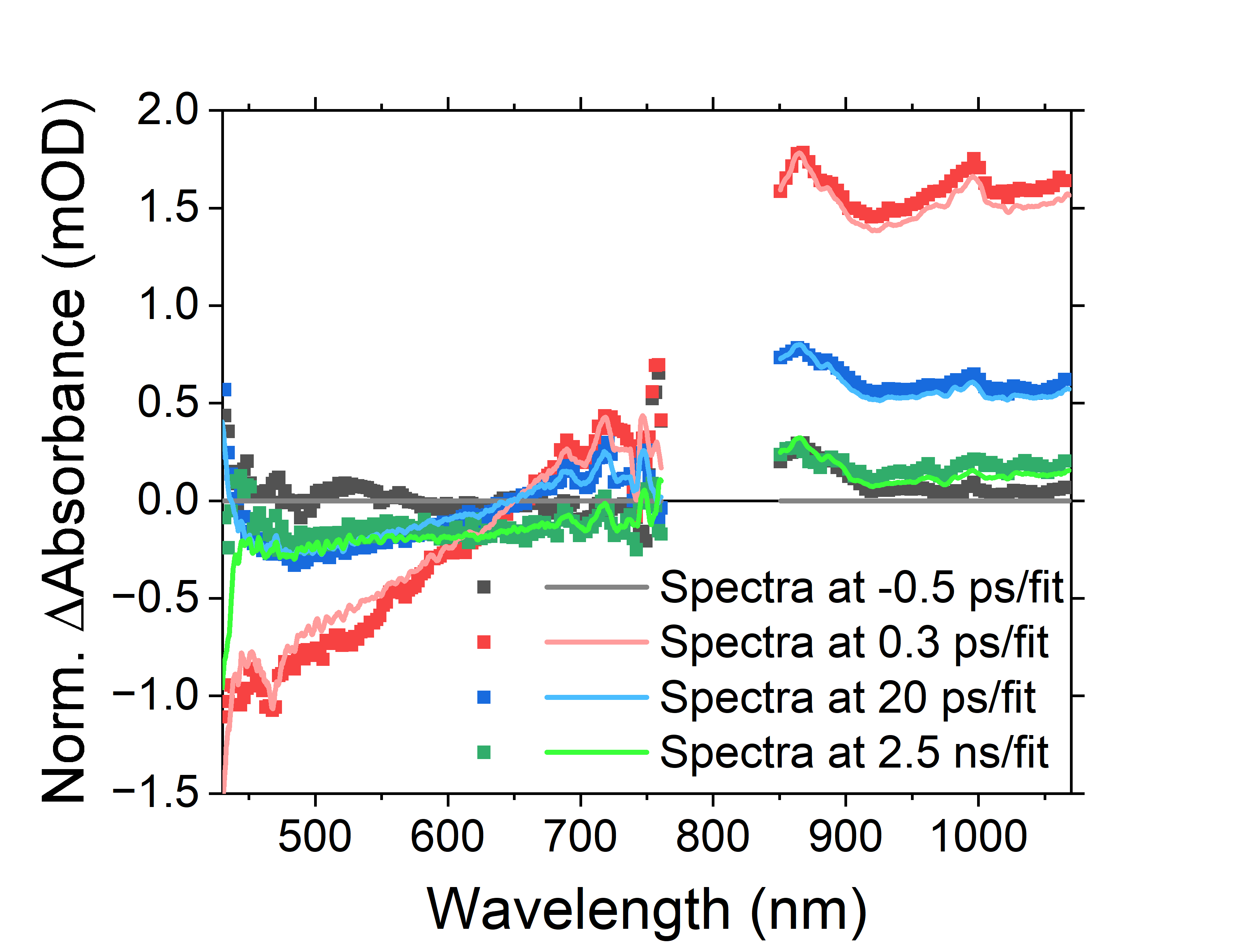


**d**

**c**

**Figure S31. a)** The fs-TAS decays normalized to absorbances at 400 nm of 2.5% AC (500) and KPHI (500) monitored at 482 nm. The first picosecond is on a linear scale and the rest is on a logarithmic scale. The fs-TAS spectra at different time delays for **b)** 2.5% AC (500) + 10 % Glycerin, **c)** KPHI (500) and, **d)** KPHI (500) + 10 % Glycerin. The spectra are cut off around 800 nm due to the fundamental laser pulse. Measurements were made with 400 nm, 0.1 mJ/cm^2^ excitation.


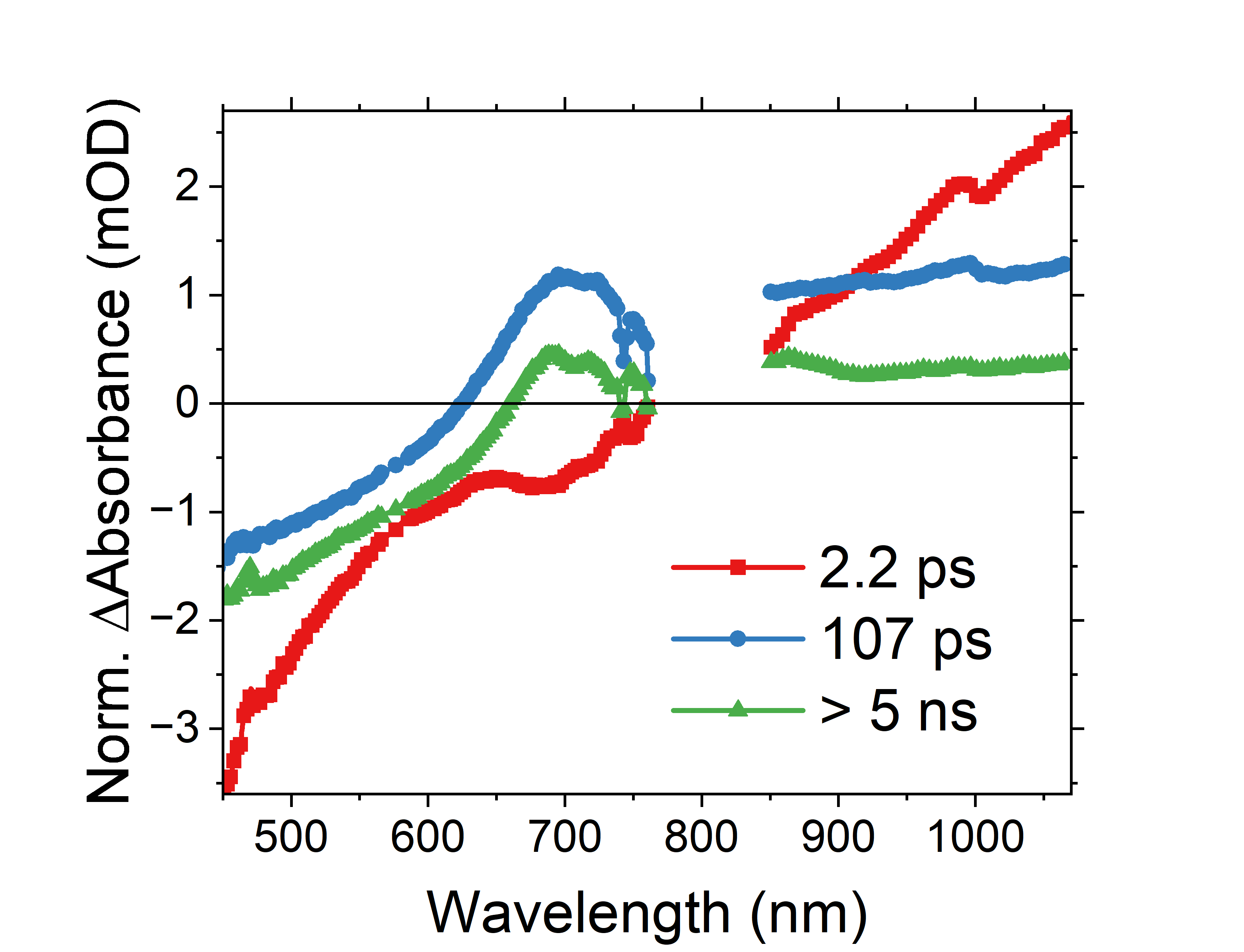

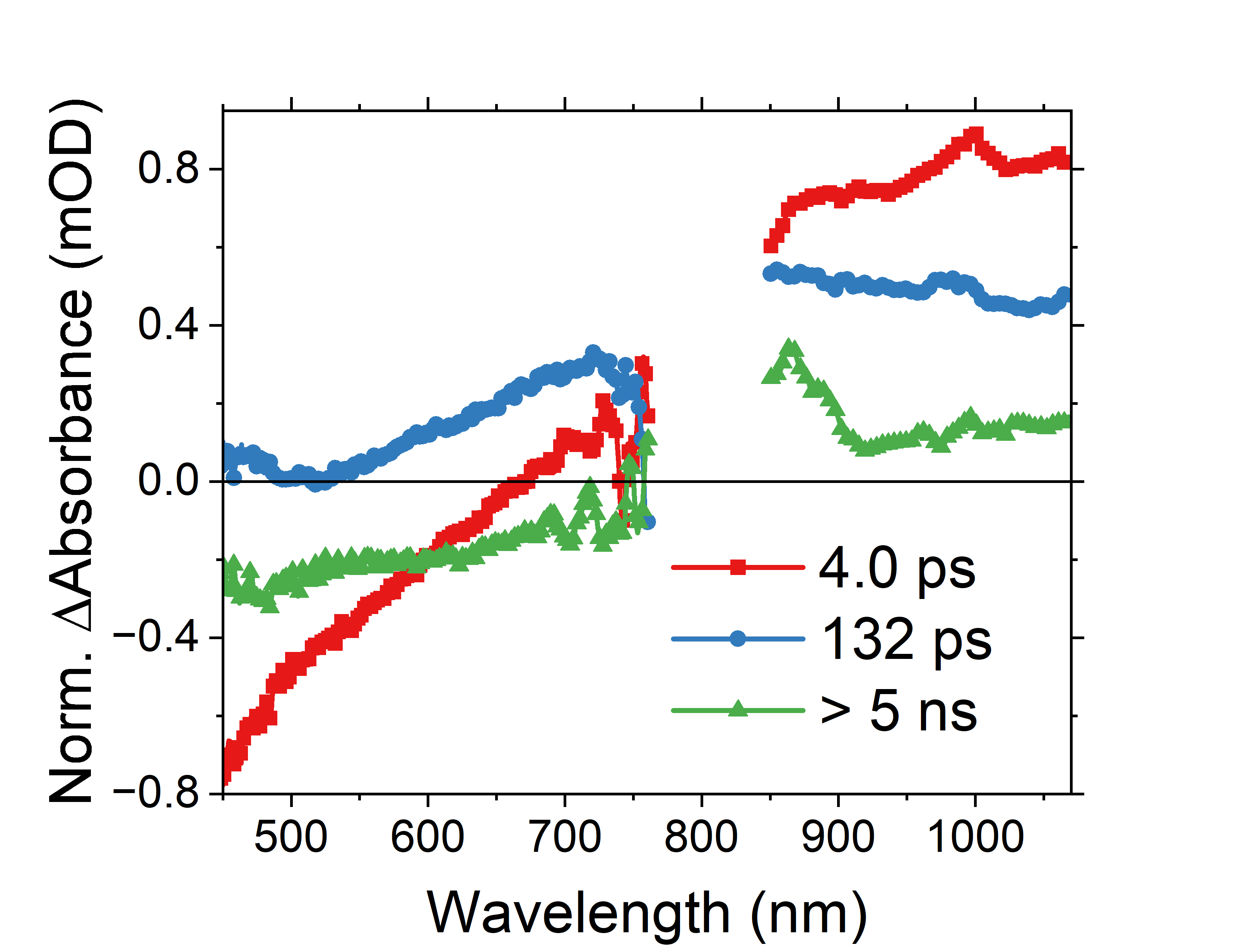

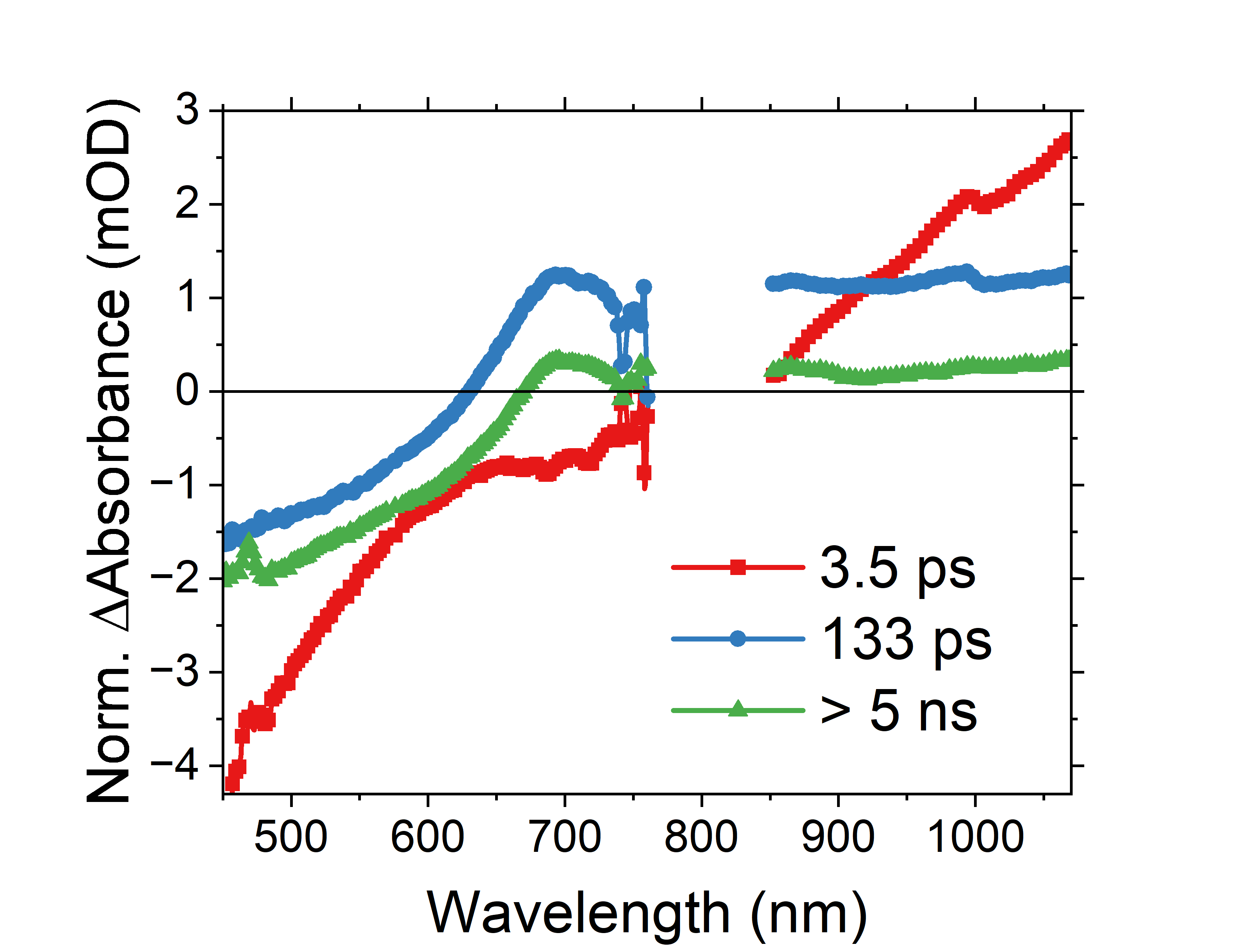

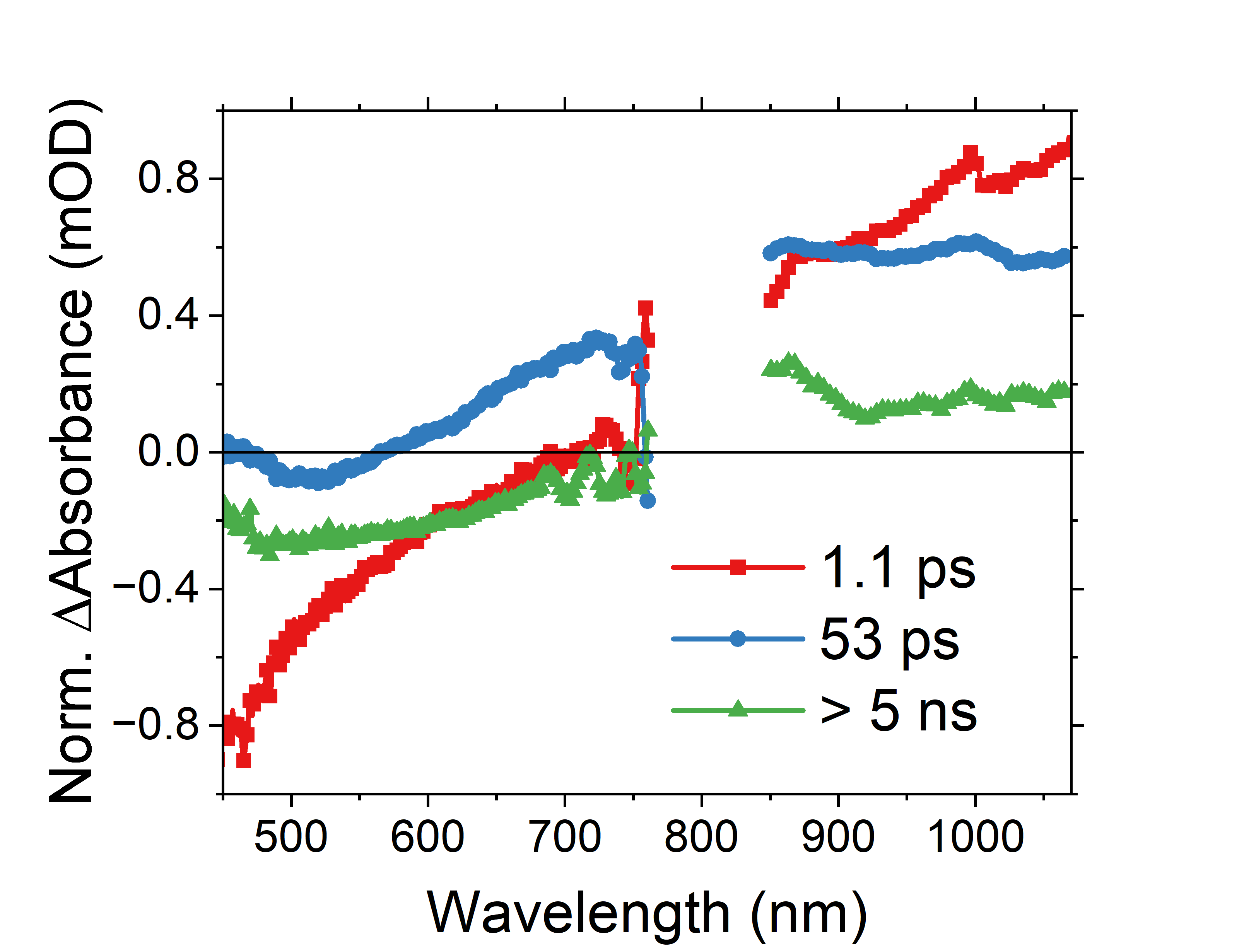


**d**

**b**

**c**

**a**

**Figure S32.** The decay associated spectra of **a)** KPHI (500), **b)** KPHI (500) + 10 % Glycerin, **c)** 2.5% AC (500) and, **d)** 2.5% AC (500) + 10 % Glycerin derived from the multiexponential fitting.

**c**

**a**

**b**


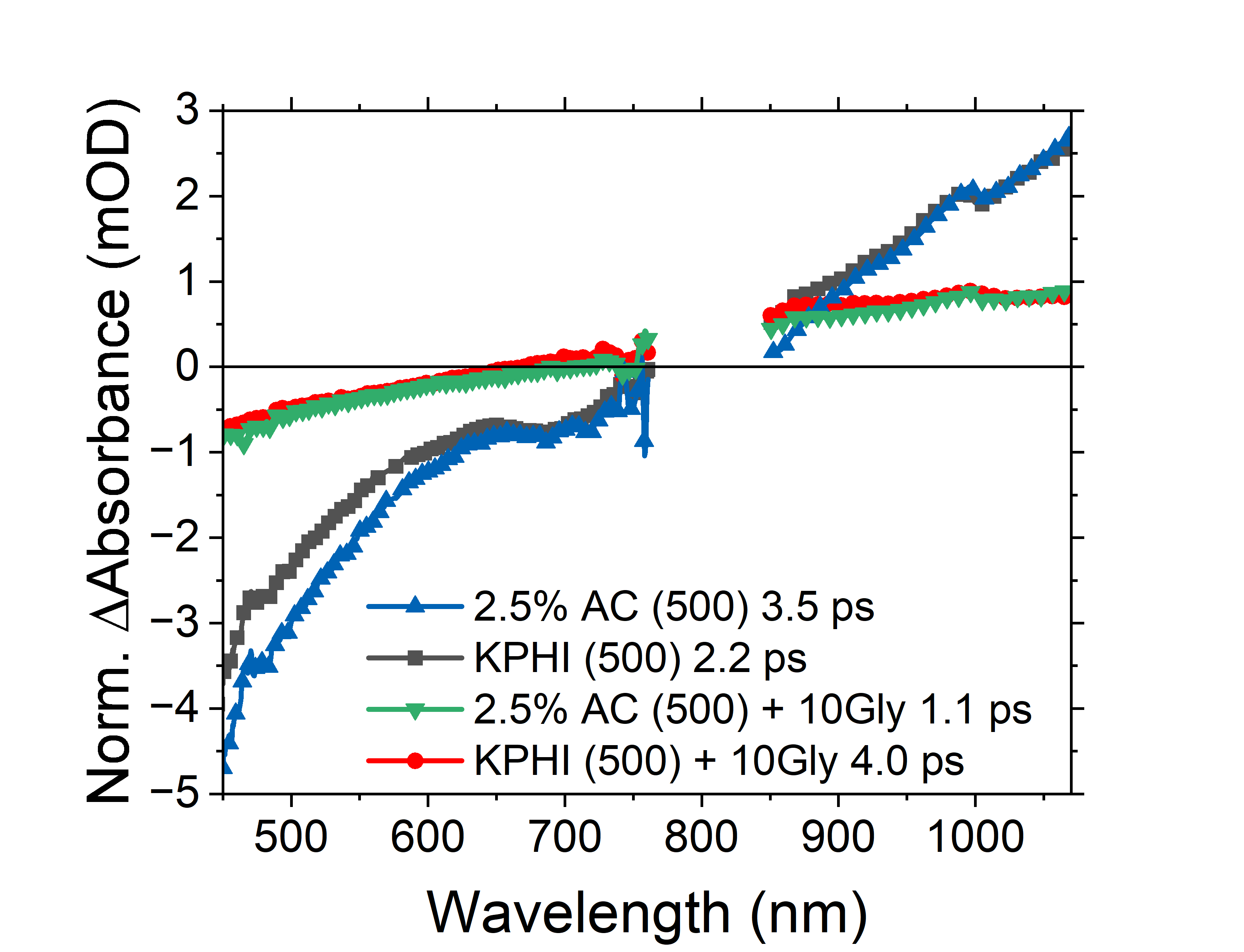

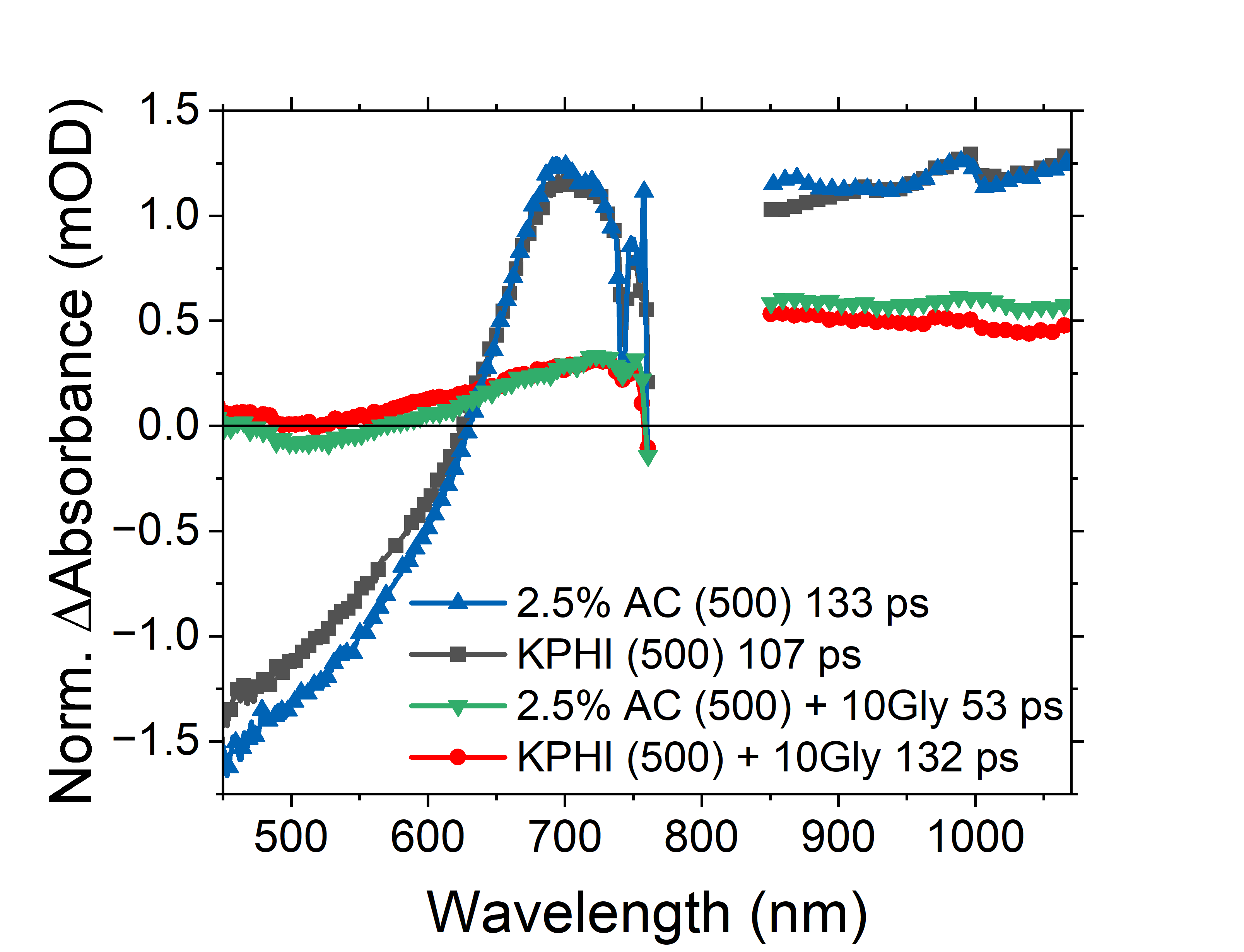

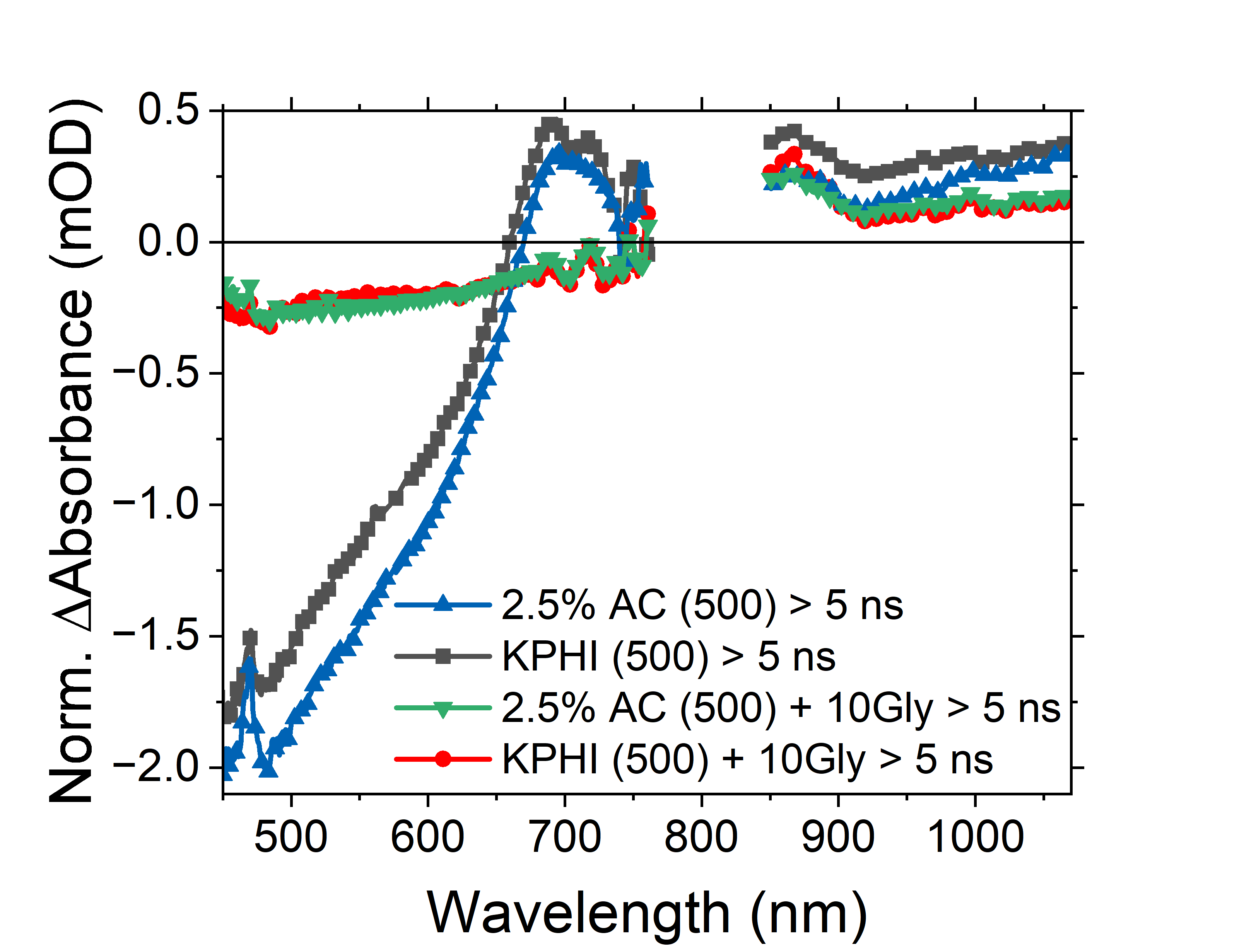


**Figure S33.** **a)** The decay associated spectra of 2.5% AC (500) and KPHI (500) of **a)** first **b)** second and, **c)** third non-scattering exponents derived from the multiexponential fitting.


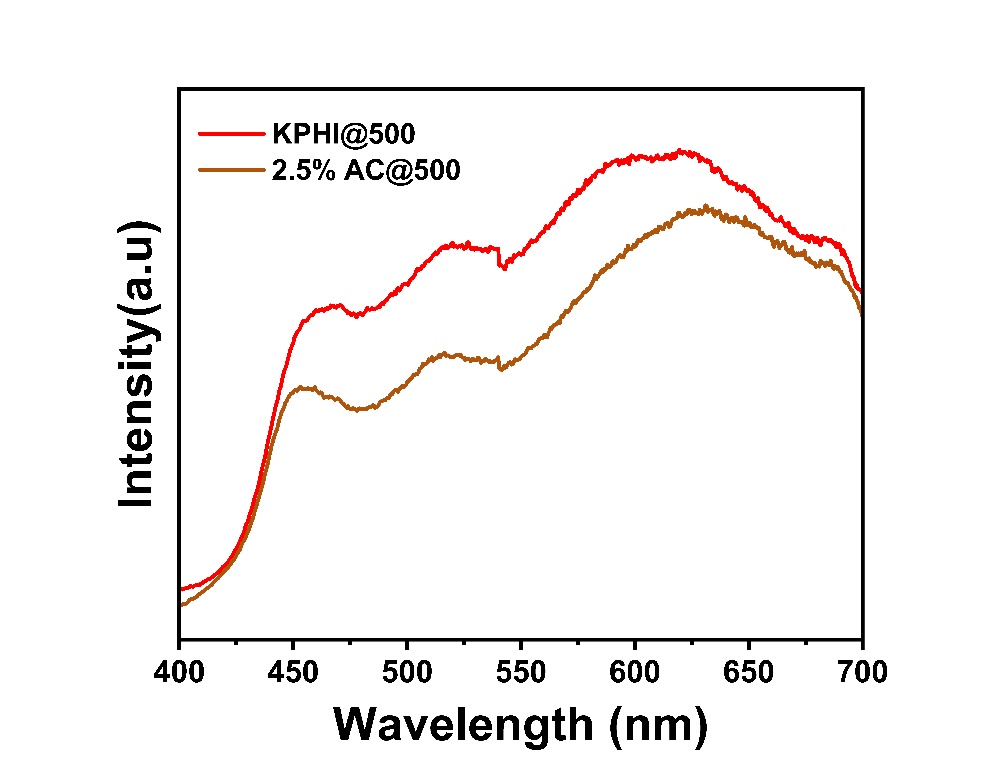


**Figure S34.** Room temperature steady-state PL emission spectra of KPHI@500 and 2.5% AC@500


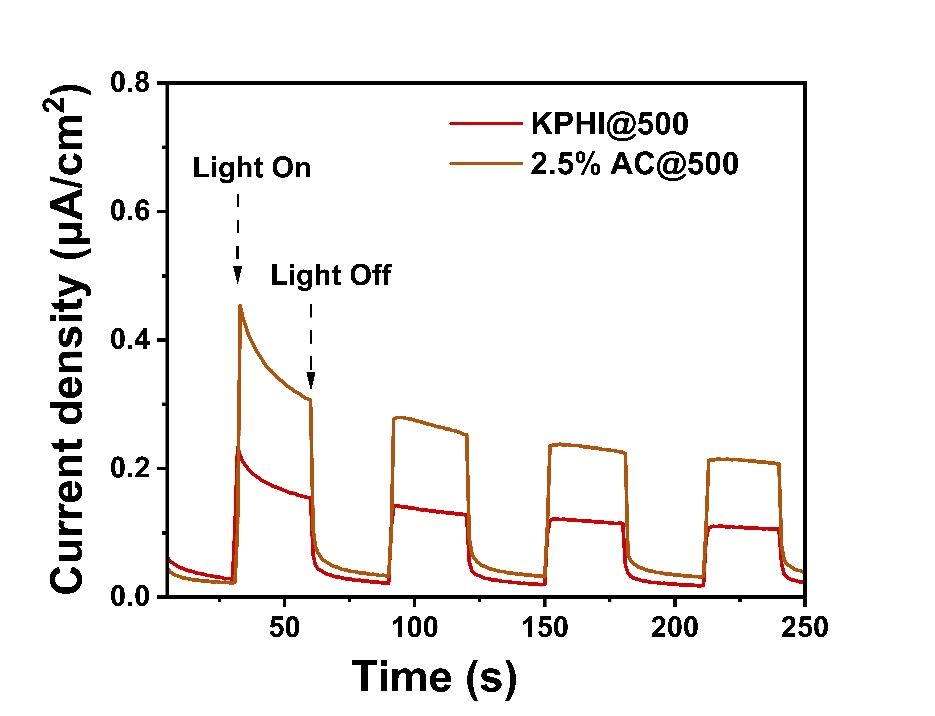


**Figure S35.** Transient photocurrent (λ = 410 nm) for KPHI@500 and 2.5%AC@500 in 0.2 m Na_2_SO_4_ aqueous solution.

**References:**

1. Grimme, S.; Hansen, A.; Ehlert, S.; Mewes, J.-M., r2SCAN-3c: A “Swiss army knife” composite electronic-structure method. The Journal of Chemical Physics 2021, 154 (6).

2. Neese, F.; Wennmohs, F.; Becker, U.; Riplinger, C., The ORCA quantum chemistry program package. The Journal of chemical physics 2020, 152 (22).

3. Lu, T.; Chen, F., Multiwfn: A multifunctional wavefunction analyzer. Journal of computational chemistry 2012, 33 (5), 580-592.

4. Momma, K.; Izumi, F., VESTA: a three-dimensional visualization system for electronic and structural analysis. Applied Crystallography 2008, 41 (3), 653-658.

5. Chang, X.; Yang, J.; Han, D.; Zhang, B.; Xiang, X.; He, J. Enhancing Light-Driven Production of Hydrogen Peroxide by Anchoring Au onto C3N4 Catalysts. *Catalysts* **2018**, *8* (4), 147.

6. Bai, Q.; Huang, Y.; Chen, Z.; Pan, Y.; Zhang, X.; Long, Q.; Yang, Q.; Wu, T.; Xie, T.-Z.; Wang, M.; Luo, H.; Hu, C.; Wang, P.; Zhang, Z. Terpyridine-Based Metallo-Cuboctahedron Nanomaterials for Efficient Photocatalytic Degradation of Persistent Organic Pollutants. *Nano Res.* **2024**, *17* (8), 6833–6840.

7 Shiraishi, Y.; Kanazawa, S.; Sugano, Y.; Sakamoto, H.; Ichikawa, S.; Hirai, T. Highly Selective Production of Hydrogen Peroxide on Graphitic Carbon Nitride (g-C₃N₄) Photocatalyst Activated by Visible Light. ACS Catalysis **2014**, 3 (9), 2222–2227.

8. Wang, W.; Gu, W.; Li, G.; Xie, H.; Keung Wong, P.; An, T. Few-Layered Tungsten Selenide as a Co-Catalyst for Visible-Light-Driven Photocatalytic Production of Hydrogen Peroxide for Bacterial Inactivation. *Environ. Sci. Nano* **2020**, *7* (12), 3877–3887.

9. Zhang, X.; Su, H.; Cui, P.; Cao, Y.; Teng, Z.; Zhang, Q.; Wang, Y.; Feng, Y.; Feng, R.; Hou, J.; Zhou, X.; Ma, P.; Hu, H.; Wang, K.; Wang, C.; Gan, L.; Zhao, Y.; Liu, Q.; Zhang, T.; Zheng, K. Developing Ni Single-Atom Sites in Carbon Nitride for Efficient Photocatalytic H2O2 Production. *Nat. Commun.* **2023**, *14* (1), 7115. h

10. Shi, L.; Yang, L.; Zhou, W.; Liu, Y.; Yin, L.; Hai, X.; Song, H.; Ye, J. Photoassisted Construction of Holey Defective G-C3N4 Photocatalysts for Efficient Visible-Light-Driven H2O2 Production. *Small* **2018**, *14* (9), 1703142.

11. Zhao, C.; Shi, C.; Li, Q.; Wang, X.; Zeng, G.; Ye, S.; Jiang, B.; Liu, J. Nitrogen Vacancy-Rich Porous Carbon Nitride Nanosheets for Efficient Photocatalytic H2O2 Production. *Mater. Today Energy* **2022**, *24*, 100926.

12. Teng, Z.; Zhang, Q.; Yang, H.; Kato, K.; Yang, W.; Lu, Y.-R.; Liu, S.; Wang, C.; Yamakata, A.; Su, C.; Liu, B.; Ohno, T. Atomically Dispersed Antimony on Carbon Nitride for the Artificial Photosynthesis of Hydrogen Peroxide. *Nat. Catal.* **2021**, *4* (5), 374–384.

13. Zhang, X.; Ma, P.; Wang, C.; Gan, L.; Chen, X.; Zhang, P.; Wang, Y.; Li, H.; Wang, L.; Zhou, X.; Zheng, K. Molecular Heptazine–Triazine Junction over Carbon Nitride Frameworks for Artificial Photosynthesis of Hydrogen Peroxide. Advanced Materials **2023**, 35 (47), 2306831.

14. Zhang, X.; Ma, P.; Wang, C.; Gan, L.; Chen, X.; Zhang, P.; Wang, Y.; Li, H.; Wang, L.; Zhou, X.; Zheng, K. Unraveling the Dual Defect Sites in Graphite Carbon Nitride for Ultra-High Photocatalytic H 2 O 2 Evolution. *Energy Environ. Sci.* **2022**, *15* (2), 830–842.

15. Zhao, Y.; Zhang, P.; Yang, Z.; Li, L.; Gao, J.; Chen, S.; Xie, T.; Diao, C.; Xi, S.; Xiao, B.; Hu, C.; Choi, W. Mechanistic Analysis of Multiple Processes Controlling Solar-Driven H2O2 Synthesis Using Engineered Polymeric Carbon Nitride. *Nat. Commun.* **2021**, *12*, 3701.

16. Hu, J.; Chen, C.; Yang, H.; Yang, F.; Qu, J.; Yang, X.; Sun, W.; Dai, L.; Li, C. M. Tailoring Well-Ordered, Highly Crystalline Carbon Nitride Nanoarrays *via* Molecular Engineering for Efficient Photosynthesis of H2O2. *Appl. Catal. B Environ.* **2022**, *317*, 121723.

17. Tong, H.; Odutola, J.; Song, J.; Peng, L.; Tkachenko, N.; Antonietti, M.; Pelicano, C. M. Boosting the Quantum Efficiency of Ionic Carbon Nitrides in Photocatalytic H2O2 Evolution via Controllable n → Π* Electronic Transition Activation. *Adv. Mater.* **2024**, *36* (49), 2412753.

18. Sharma, P.; Slater, T. J. A.; Sharma, M.; Bowker, M.; Catlow, C. R. A. Enhanced H2O2 Production via Photocatalytic O2 Reduction over Structurally-Modified Poly(Heptazine Imide). *Chem. Mater.* **2022**, *34* (12), 5511–5521.
